# Decoding and reprogramming of the biosynthetic networks of mushroom-derived bioactive type II ganoderic acids in yeast

**DOI:** 10.1038/s41421-025-00812-1

**Published:** 2025-07-08

**Authors:** Qin Wang, Ye Li, Shunhan Zhang, Wei Yuan, Zeqian Du, Ting Shi, Zhao Chang, Xingye Zhai, Yinhua Lu, Meng Wang, Juan Guo, Jian-Jiang Zhong, Han Xiao

**Affiliations:** 1 State Key Laboratory of Microbial Metabolism, Joint International Research Laboratory of Metabolic & Developmental Sciences, School of Life Sciences and Biotechnology, Shanghai Jiao Tong University, Shanghai, China; 2 Key Laboratory of Engineering Biology for Low-Carbon Manufacturing, Tianjin, China; 3 College of Life Sciences, Shanghai Normal University, Shanghai, China; 4 State Key Laboratory for Quality Ensurance and Sustainable Use of Dao-di Herbs, National Resource Center for Chinese Materia Medica, China Academy of Chinese Medical Science, Beijing, China

**Keywords:** Molecular biology, Biological techniques

## Abstract

Mushroom’s specialized secondary metabolites possess important pharmacological activities, but their biosynthetic pathway elucidation is extremely challenging, not to mention reprogramming of their biosynthetic networks to target metabolites. By taking *Ganoderma lucidum*, a famous traditional medicinal mushroom, as a lead example, here we decoded the biosynthetic networks of type II ganoderic acids (TIIGAs), a group of its main bioactive metabolites by studying the coordinated gene expression in *G. lucidum*, identifying endogenous or heterologous enzymes capable of C22 hydroxylation, configuration conversion of C3 hydroxyl group, and acetylation on C3, C15 and C22 hydroxyl groups. Notably, we revealed the catalytic mechanism of the C22 hydroxylase CYP512W6, and an unexpected bifunctional acetyltransferase GlAT that is required to transfer acetyl groups to C15 and C22. Using a fluorescence-guided integration method, we achieved efficient biosynthesis of significant TIIGAs applicable to industrial fermentation. After introducing all the identified enzymes to baker’s yeast, we observed that biosynthesis of downstream TIIGAs was severely impeded, and dredged the metabolic block by temporally regulating the expression of acetyltransferases. By reprogramming of the biosynthetic networks of TIIGAs, we were able to produce over 30 TIIGAs, exhibiting 1–4 orders of magnitude higher titers or efficiencies than those from farmed mushrooms. The work enables the access to valuable TIIGAs, facilitates their widespread application, and sheds light on research of other mushroom products.

## Introduction

Mushrooms are unique: used as a medicine for the ill, as a tonic (dietary supplement) for those in a sub-health state as well as both healthy and ill individuals, and as a food for health people worldwide. Medicinal mushrooms and their products are credited with enhancing the overall health and well-being of humankind, which have been used to treat and prevent human diseases^1^. Possessing with specified metabolites, they exhibit a wide range of therapeutic properties including anti-cancer, hepatoprotective, anti-diabetic and cholesterol-lowering activities as well as protection against atherosclerosis, cardiovascular, chronic inflammatory, autoimmune and neurodegenerative diseases^2^. Mushrooms represent an abundant yet largely untapped source of medically relevant natural products^3–5^. In contrast to the vast global market of the mushroom industry and the importance of mushroom-derived compounds, the related basic research is overlooked and greatly lags far behind that of plants and microbes^6,7^.

One remarkable example of mushrooms is the traditional Chinese medicinal *Ganoderma lucidum*. It has over 2000 years of folk medicine history in China and its product market occupies around 5 billion US$ in 2024 with a compound annual growth rate of 9.0%^8^. Its significant pharmacological properties are due to a group of its specific secondary metabolites, lanostane-type triterpenoid ganoderic acids (GAs). According to the number of double bonds on the skeleton, GAs can be divided into type I GAs, with one double bond at C8 and C9, and type II GAs (TIIGAs), with conjugated double bonds at C7, C8 and C9, C11^9^. TIIGAs significantly inhibit the proliferation of more than twenty kinds of cancer cells, among which GA-Me and GA-T can also inhibit the migration of cancer cells (Supplementary Table S1)^10–12^. In addition, TIIGAs have abundant biological activities such as hypotensive, hepatoprotective and anti-inflammatory activities (Supplementary Table S1). Although their entire bioactivity working mechanisms remain obscure, they are thought to result from the unique conjugated double bond on the tetracyclic ring, multi-oxidation on C3, C15, C22, and C26, and multi-acetylation on C3, C15, and C22^13–18^. Such selective oxidation cannot be achieved through conventional chemical synthesis, nor can the subsequent post-modifications. In addition, the immature genetic manipulation, the long growth cycle, and the susceptibility to environmental factors (e.g., land, climate, and pests) collectively render the *G. lucidum* farming-based supply chain for producing TIIGAs problematic (Supplementary Table S2).

**Fig. 1:**
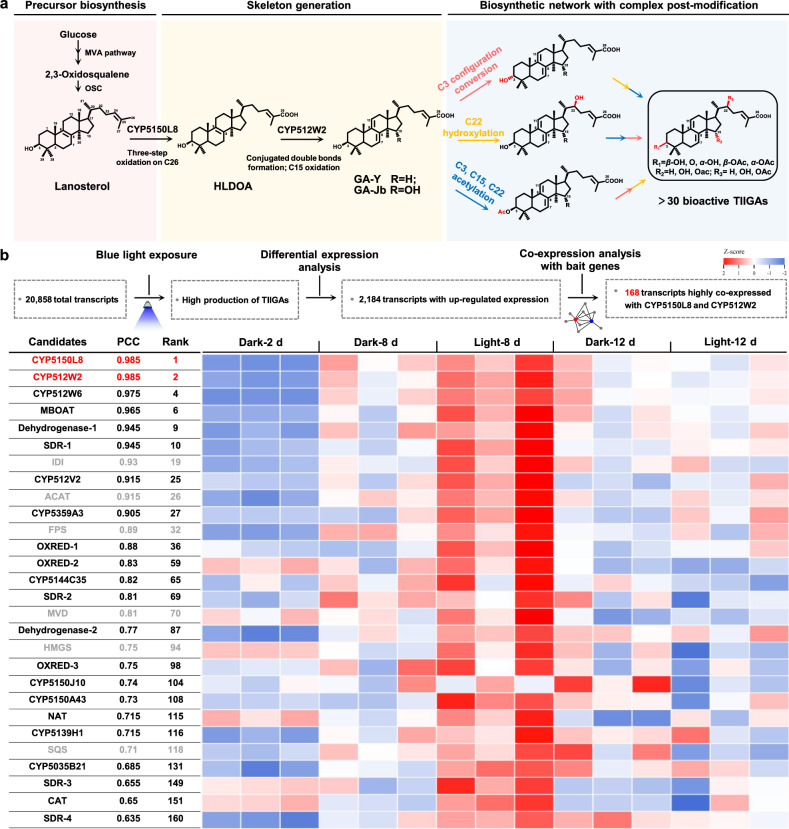
Analysis of the biosynthetic network of TIIGAs in *G. lucidum.* **a** A series of unknown post-modification enzymes and their possible working sequences. Early steps in the biosynthesis of TIIGAs lead to the generation of the precursor lanosterol via the mevalonate (MVA) pathway (highlighted in light pink). Two previously characterized CYPs from *G. lucidum* facilitate the conversion of lanosterol to HLDOA, and to GA-Y and GA-Jb (highlighted in light yellow). A series of complex post-modifications, including C3 configuration conversion (highlighted in red arrow), C22 hydroxylation (highlighted in yellow arrow), and C3, C15, C22 acetylation (highlighted in blue arrow), are necessary for the formation of the end TIIGAs (highlighted in light blue). OSC, oxidosqualene cyclase. **b** Transcriptomic analysis of *G. lucidum* for identifying enzyme candidates. Co-expression analysis of *G. lucidum* RNA samples under dark and blue light exposure (Supplementary Table S3) using *cyp5150l8* and *cyp512w2* as bait genes. Linear regression analysis is employed to screen genes with PCC > 0.6 relative to the baits. PCC values are averages calculated using two baits. Enzymes highlighted in red refer to the baits used for the analysis, enzymes highlighted in black refer to the functional enzymes associated with unknown steps in biosynthesis of TIIGAs, and enzymes highlighted in gray indicate enzymes in the MVA pathway. For the remaining candidates, see Supplementary Table S4. The heatmap displays the *Z*-score calculated from log_2_-normalized expression, with three replicates for each sample. The heatmap is drawn using https://www.chiplot.online/.

The radioactive isotope labeling study suggested that GAs were biosynthesized from lanosterol via the mevalonate pathway in *G. lucidum*^19^. Although enzymes that catalyze the conversion of acetyl-CoA to form lanosterol are conserved in eukaryotic cells, those responsible for extensive post-modifications on the lanosterol skeleton remain elusive. Using the baker’s yeast *Saccharomyces cerevisiae* as the synthetic biology chassis, and through long-term efforts, we have partially elucidated the biosynthesis of TIIGAs, including the three-step successive oxidations on C26 by CYP5150L8^20^, and the formation of the signature structure of TIIGAs, the conjugated double bonds at C7, C8 and C9, C11, and the subsequent oxidation on C15 by a promiscuous CYP512W2^21^. To decode the biosynthetic network of TIIGAs, we must find enzymes that catalyze the configuration conversion of the C3 hydroxyl group, C22 hydroxylation, and acetylation on the C3, C15, and C22 hydroxyl group (Fig. 1a). The configuration conversion of the hydroxyl group can be catalyzed by a pair of oxidase and reductase^22–24^, or by one epimerase instead^25,26^. The remaining steps involve cytochrome P450 (CYP) and acetyltransferase (AT). Due to the diversity of these post-modification enzymes and the uncertainty regarding the catalytic order, there are multiple possibilities for the biosynthesis of terminal TIIGA, which makes decoding of the TIIGAs’ biosynthetic network an incredibly challenging task (Fig. 1a).

With the aid of an automated system, we screened 158 CYPs derived from *G. lucidum* in the GA-Jb producing yeast, but were not able to identify its C22 hydroxylase^21^. In this work, we attempted to identify this set of missing enzymes by skillfully prioritizing the candidates through systematic transcriptome, phylogenetic, and homologous analysis. Along with the elucidation of the catalytic mechanisms of key enzymes and the adoption of gene expression regulation system in baker’s yeast, we successfully reconstituted the biosynthetic routes of over 30 clinical candidate TIIGAs to support their widespread application.

## Results

### Narrowing down the post-modification gene candidate

To identify the enzymes involved in the C3 hydroxyl configuration conversion, crude protein lysates from *G. lucidum* mycelia were employed for an in vitro activity assay using GA-Jb as the substrate. Liquid chromatography-mass spectrometry (LC-MS) analysis showed that compound **1** with a primary *m/z* at 451 was generated, which is likely corresponding to a dehydrogenation product of hydroxyl group on the triterpenoid backbone (Supplementary Fig. S1). To determine the structure, we expanded the reaction system and isolated 3 mg of compound **1**. Nuclear magnetic resonance (NMR) analyses indicated that the C3 hydroxyl of GA-Jb was transformed into a ketone group (Supplementary Table S8, Figs. S2–S7 and Note S1), and the chemical structure of compound **1** was GA-TR. This result suggests that the configuration conversion of the C3 hydroxyl group may be accomplished through a two-step reaction, namely the oxidation of the C3 hydroxyl group followed by the reduction of the C3 keto group.

TIIGAs predominantly accumulate in the mycelia, which is the early developmental stage of *G. lucidum*, and their production decreases with growth under unknown regulation mechanisms^27–30^. To decode their biosynthetic pathway, we employed a blue light exposure strategy (Supplementary Fig. S8) to specifically stimulate the production of TIIGAs in mycelia, thereby making the co-expression pattern with the biosynthetic pathway genes more evident. After 12 days of cultivation, the content of TIIGAs GA-Mk, GA-T, GA-S, and GA-R was 2.8 mg/g, 6.3 mg/g, 7.3 mg/g, and 2.7 mg/g dry weight (DW), respectively, which was increased by 2.9-, 3.7-, 3.7-, and 3.2-fold, respectively, compared with those cultured under dark conditions (Supplementary Fig. S8c). The mycelia on day 2, day 8, and day 12, representing almost no production, rapid production, and stable production stages of TIIGAs, respectively, were selected for RNA sequencing. Compared with the transcriptional expression profiles under dark conditions, 2184 transcripts were upregulated for expression under blue light exposure. We used CYP5150L8 and CYP512W2, which are key enzymes in the biosynthesis of TIIGAs^20,21,31^, as bait genes for co-expression analysis, and a total of 168 transcripts, including 8 CYPs, 3 ATs, and 9 dehydrogenases/reductases, were highly correlated (Pearson’s correlation coefficient (PCC) > 0.6) with their expression (Fig. 1b; Supplementary Table S4).

Based on the substrate structural similarity to TIIGAs, we also systematically sorted out possible heterologous candidates (Supplementary Table S5). A total of 12 CYPs, 8 ATs, and 11 dehydrogenases/reductases were thus selected.

### Identification of heterologous oxidase and reductase for efficient configuration conversion of C3 hydroxyl group

**Fig. 2:**
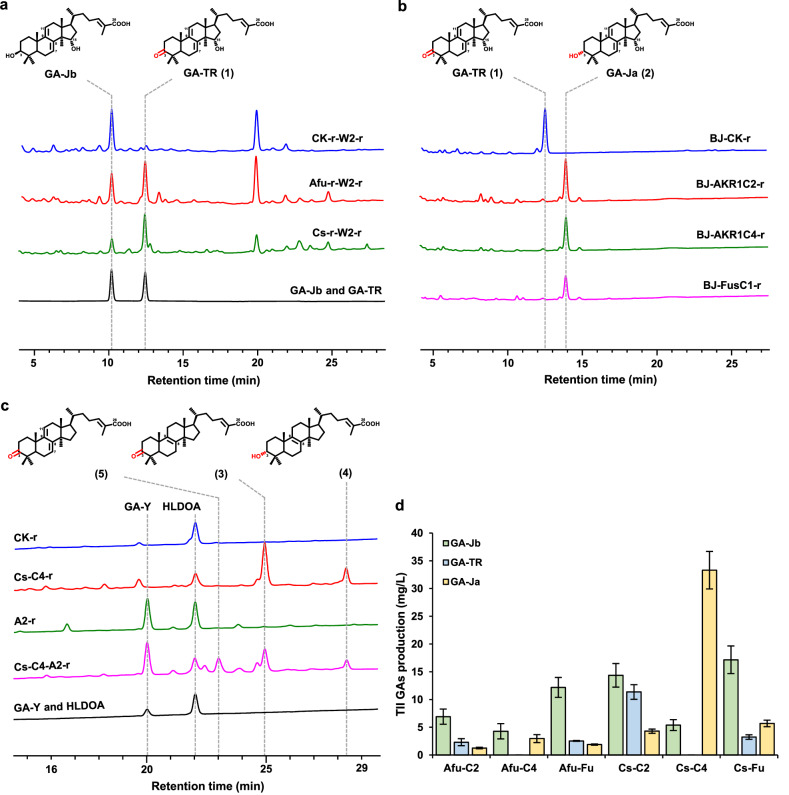
Functional characterization of C3 oxidase and reductase. **a** HPLC analysis of fermentation extracts of strains SC62-CK-r-CYP512W2-r (CK-r-CYP512W2-r), SC62-AfuSDR-r-CYP512W2-r (Afu-r-W2-r), and SC62-CsSDR-r-CYP512W2-r (Cs-r-W2-r). **b** HPLC analysis of fermentation extracts of engineered *S. cerevisiae* strains BJ5464-CK-r (BJ-CK-r), BJ5464-AKR1C2-r (BJ-AKR1C2-r), BJ5464-AKR1C4-r (BJ-AKR1C4-r), and BJ5464-FusC1-r (BJ-FusC1-r) after incubation with GA-TR for 48 h. **c** HPLC analysis of the fermentation extracts of strains SC62-CsSDR-AKR1C4-r (Cs-C4-r), SC62-CK-r (CK-r), SC62-CsSDR-AKR1C4-CYP512A2-r (Cs-C4-A2-r), and SC62-CYP512A2-r (A2-r). **d** Production of GA-Jb, GA-TR, and GA-Ja by strains Afu-C2, Afu-C4, Afu-Fu, Cs-C2, Cs-C4, and Cs-Fu after 120 h fermentation. All data represent the mean of three independent samples, and the error bars indicate the standard deviation.

From the very beginning, we attempted to in situ isolate C3 oxidase from the crude protein lysates of *G. lucidum* by tracking the C3 oxidation activity of GA-Jb, but we failed. Then, we turned to test heterologous alternatives. To identify the enzymes responsible for the configuration conversion of the C3 hydroxyl group, heterologous dehydrogenase candidates were introduced into the GA-Jb producing *S. cerevisiae* strain SC62-CYP512W2-r. Compare to the control strain SC62-CK-r-CYP512W2-r, we observed that short-chain dehydrogenase/reductases from *Aspergillus fumigatus*^32^ (AfuSDR) and *Citrus sinensis*^33^ (CsSDR) were able to catalyze the conversion of GA-Jb to generate compound **1** (Fig. 2a). The retention time (12.3 min) and mass spectrometry information (with a primary *m/z* at 451) of compound **1** matched those of GA-TR (Fig. 2a; Supplementary Fig. S9), indicating that the C3*β*-hydroxyl group was oxidized to a ketone group. After confirming that AfuSDR and CsSDR were the C3 oxidases, we identified JG8773.T2 and JG4122.T1, which exhibited the highest amino acid homology with AfuSDR (21.01%) and CsSDR (24.81%) across the genome of *G. lucidum*, respectively. After several attempts under different PCR conditions, we failed to obtain the coding regions of *jg8773.t2*. We cloned *jg4122.t1*, and heterologously expressed it in the GA-Jb producing yeast, but did not detect C3-oxidized GA-TR from the yeast fermentation extracts.

To screen the subsequent C3 keto reductase of GA-TR, we first confirmed that *S. cerevisiae* BJ5464 was the optimal screening host for whole-cell catalysis among BJ5464, INVSc1 and BY4741, due to its superior permeability towards TIIGA^34^ (Supplementary Fig. S10). When reductases were individually introduced into *S. cerevisiae* BJ5464 and incubated with ~50 μM GA-TR for 48 h, compared with the strain containing the void plasmid, AKR1C2 and AKR1C4 from *Homo sapiens*^35^, and FusC1 from *Acremonium fusidioides*^36^ were able to completely convert GA-TR into compound **2**, with a primary *m/z* at 453, indicating that a reduction product was generated (Fig. 2b; Supplementary Fig. S9).

To obtain a large amount of compound **2** for chemical structure confirmation, we randomly grouped two oxidases (CsSDR and AfuSDR) and three reductases (AKR1C2, AKR1C4, and FusC1) into six oxidase-reductase pairs, and introduced them together with CYP512W2 into the HLDOA-producing yeast SC62 (Supplementary Tables S5–S7). The co-expression of CsSDR and AKR1C4 produced 33.3 mg/L compound **2** and accumulated 5.4 mg/L GA-Jb, with no detectable GA-TR (Fig. 2d; Supplementary Fig S11). In contrast, the yield of compound **2** in other strains was only 1.3 mg/L, 3.0 mg/L, 1.9 mg/L, 4.3 mg/L, and 5.7 mg/L, accompanied by accumulated GA-Jb and unconverted GA-TR (Fig. 2d; Supplementary Fig. S11). Fermentation of 5 L strain SC62-CsSDR-AKR1C4-CYP512W2-r was subsequently performed, and 13.2 mg of compound **2** was obtained. The NMR spectra revealed that the chemical structure of compound **2** was 3*α*,15*α*-dihydroxylanosta-7,9(11),24-trien-26-oic acid (GA-Ja) (Supplementary Table S8, Figs. S12–S18 and Note S1).

To investigate whether the oxidation-reduction-mediated configuration conversion of the C3 hydroxyl group can be applied to other GAs, we further introduced CYP512A2, CsSDR, and AKR1C4 into HLDOA-producing yeast SC62. Compared to the control strain SC62-CK-r, the fermentation extracts of SC62-CsSDR-AKR1C4-r showed two new peaks, which might respectively correspond to the C3 hydroxyl oxidation product (peak **3**, *m/z* 437) and the configuration conversion product (peak **4**, *m/z* 439) of HLDOA (Fig. 2c; Supplementary Fig. S9). Peaks 3 and 4 were also detected in SC62-CsSDR-AKR1C4-CYP512A2-r compared to the control strain SC62-CYP512A2-r (Fig. 2c). As for another new peak with the primary *m/z* at 453, we speculated that peak **5** was the product of the C3 keto group of GA-Y (Fig. 2c; Supplementary Fig. S9). The conversion of the C3 hydroxyl group of HLDOA and GA-Y by CsSDR and AKR1C4 was not as efficient as that of GA-Jb.

### Identifying CYP512W6 as a C22 hydroxylase and elucidating its catalytic mechanism

To rapidly determine whether the suspicious compound is the C22-hydroxylated product, we prepared a series of C22-hydroxylated GAs as standards to guide the enzyme identification. Triple acetylated GA-T and double acetylated GA-R were isolated from dried *G. lucidum* mycelia as reaction substrates for alkaline hydrolysis to remove the acetyl group of C22. Peaks **6** and **7**, with primary *m/z* at 469 and 451, respectively, were the main hydrolysis products of GA-T. Peaks **8** and **9**, both with primary *m/z* at 453, were the main hydrolysis products of GA-R (Supplementary Fig. S19). We ultimately obtained 6.0 mg, 1.1 mg, 2.0 mg, and 2.5 mg corresponding to peaks **6**, **7**, **8** and **9**, respectively, for their chemical structure determination. They were identified by NMR analysis as 3*α*,15*α*,22*β*-trihydroxy-lanosta-7,9(11),24-trien-26-oic acid (TLTOA), 3*α*-acetoxy-15*α*,22*β*-dihydroxy-lanosta-7,9(11),24-trien26-oic-acid (GA-T2), 3*α*,22*β*-dihydroxylanosta-7,9(11),24-trien-26-oic acid (DLTOA) and 3*α*-acetoxy-22*β*-hydroxy-lanosta-7, 9(11),24- trien-26-oic acid (3*α*-acetoxy-22*β*-HLTOA), respectively (Supplementary Table S8, Figs. S20–S43 and Note S1).

**Fig. 3:**
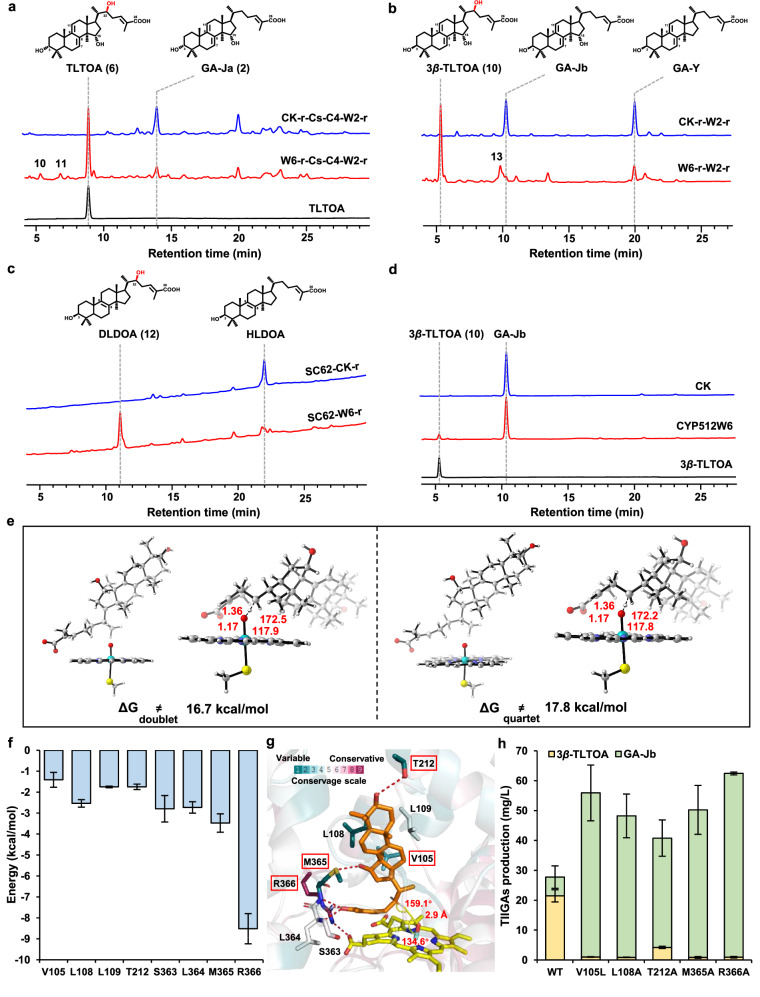
Discovery of CYP512W6 as a C22 hydroxylase. **a** HPLC analysis of the fermentation extracts of strains SC62-CK-r-CsSDR-AKR1C4-CYP512W2-r (CK-r-Cs-C4-W2-r), SC62-CYP512W6-r-CsSDR-AKR1C4-CYP512W2-r (W6-r-Cs-C4-W2-r). **b** HPLC analysis of the fermentation extracts of strains SC62-CYP512W6-r-CYP512W2-r (W6-r-W2-r) and SC62-CK-r-W2-r (CK-r-W2-r). **c** HPLC analyses of the fermentation extracts of strains SC62-CK-r, and SC62-CYP512W6-r (SC62-W6-r). **d** HPLC analysis of the in vitro enzymatic extracts by incubating CYP512W6-containing microsomes (prepared from strain CYP512W6-r-iGLCPR-r) with GA-Jb. CK indicates microsomes prepared from the control strain CK-r-iGLCPR-r. **e** The optimized structures and transition states with their respective energy barriers for C22 hydroxylation of spin 2 and spin 4. **f** Eight residues contributed more than 1.4 kcal/mol in binding affinity calculated by MM/GBSA. **g** The residues with binding energies more than 1.4 kcal/mol and their conservativeness. Hydrogen bond and sulfur–hydrogen bond are shown as red dots. Residues with high variability are selected for experimental verification (circled by red rectangle, including conservative R366). The conservation is calculated by the ConSurf website. **h** Production of 3*β*-TLTOA and GA-Jb by strains SC62-CYP512W6-r-CYP512W2-r (WT), SC62-V105L-r-CYP512W2-r (V105L), SC62-L108A-r-CYP512W2-r (L108A), SC62-T212A-r-CYP512W2-r (T212A), SC62-M365A-r-CYP512W2-r (M365A), and SC62-R366A-r-CYP512W2-r (R366A) after 120 h of fermentation.

Candidate CYPs were individually introduced into the GA-Ja production yeast. Only the expression of CYP512W6 could generate three products, with primary *m/z* values at 469 (peak **6**), 469 (peak **10**) and 467 (peak **11**), in the LC-MS analysis of 120 h fermentation extracts (Fig. 3a; Supplementary Fig. S44). The retention time and mass spectra information of product **6** were identical to those of TLTOA, the C22-hydroxylated product of GA-Ja (Fig. 3a; Supplementary Fig. S44). For products **10** and **11**, with an increased *m/z* of 16 compared to those of GA-Jb and GA-TR, we speculated that they might be the C22-hydroxylated products of them (Supplementary Fig. S44). To determine whether CYP512W6 can catalyze C22 hydroxylation on other GAs, we also introduced it into the HLDOA-producing strain SC62, and the GA-Y and GA-Jb producing strain SC62-CYP512W2-r. Compared with the control strains, the strain SC62-CYP512W6-r generated one new peak, while the strain SC62-CYP512W6-r-CYP512W2-r generated two after 120 h fermentation (Fig. 3b, c). Their primary *m/z* values were 455 (peak **12**), 469 (peak **10**), and 453 (peak **13**), which might correspond to the hydroxylation products of HLDOA, GA-Jb, and GA-Y, respectively (Supplementary Fig. S44). We further performed a 5 L fermentation of CYP512W6 expression strains, obtained 27.9 mg of peak **12**, 18.0 mg of peak **10**, and 10.1 mg of peak **6**, and then confirmed their structures by NMR analyses. The NMR spectra of peak **6** were identical to those of TLTOA. The chemical structures of peaks **10** and **12** are 3*β*,15*α*,22*β*-trihydroxylanosta-7,9(11),24-trien-26-oic acid (3*β*-TLTOA) and 3*β*,22*β*-dihydroxylanosta-8,24-dien-26-oic acid (DLDOA), the C22 hydroxylation products of GA-Jb and HLDOA, respectively (Supplementary Table S8, Figs. S45–S56 and Note S1).

To understand the substrate preference and the reaction sequence, we prepared CYP512W6-containing yeast microsomes for an in vitro reaction with the corresponding compounds. Surprisingly, CYP512W6 could only catalyze C22 hydroxylation on GA-Jb, but not on other GAs (Fig. 3d; Supplementary Fig. S57). These results suggested that GA-Jb was the most suitable substrate for CYP512W6 among the tested substrates. More specifically, 3*β*-TLTOA was generated preferentially by CYP512W6, and then underwent configuration conversion of the C3 hydroxyl group to generate TLTOA. As for the inconsistency between the in vivo and in vitro characterization of the C22 hydroxylation activity of CYP512W6 on HLDOA and GA-Y, we speculated that these GAs might not be favored by CYP512W6.

We compared the sequences among 12 heterologous CYPs capable of C22 post-modification (hydroxylation, oxidation, and dehydrogenation) of GA-like substrates (Supplementary Table S5) and CYP512W6. Interestingly, the sequence of CYP512W6 was quite different from the others. The highly conserved residues, such as P105 and H365, were changed to V and M at the corresponding positions in CYP512W6, suggesting the uniqueness of CYP512W6 (Supplementary Fig. S58). We further synthesized all these hydroxylases and introduced them into the GA-Jb producing yeast. No new LC peak was generated after 120 h fermentation of the resultant strains compared to that of the control strain (Supplementary Fig. S59). Consistent with our speculation, these heterologous enzymes were not able to hydroxylate C22 of GA-Jb.

To investigate the catalytic mechanism of CYP512W6-mediated C22 hydroxylation on GA-Jb, the structure of CYP512W6 was modeled by AlphaFold2 (AF2) (Supplementary Fig. S60). Apart from variations in residues around the catalytic pocket, the overall structures of CYP512W6 and CYP512W2 are similar^37^. The position of heme in CYP512W6 was determined using CYP512W2 as a reference. GA-Jb was docked into the pocket of CYP512W6, with C22 oriented towards the reactive oxygen of O-heme of the reactive Fe (IV)-oxo radical cation (Cpd I) (Supplementary Fig. S60). In a truncated model, the energy required to hydroxylate C22 of GA-Jb was calculated using the quantum mechanics (QM) method and found to be no more than 17.8 kcal/mol (16.7 kcal/mol at spin 2 and 17.8 kcal/mol at spin 4, respectively), suggesting the feasibility of hydroxylation (Fig. 3e). To assess the stability and reactivity of GA-Jb in the pocket of CYP512W6, three independent 100 ns molecular dynamics (MD) simulations were conducted, using the docked structure as the starting point. Conformations with appropriate attacking distances (O–H distance < 3.5 Å) and angles (Fe–O–H angle ranging from 100° to 140° and C22–H–O angle ranging from 140° to 180°) were designated as pre-reaction states (PRSs), and they were observed for 2.6% of all 300 ns MD stimulations, indicating the feasibility of C22 hydroxylation (Supplementary Fig. S61). QM calculations and MD simulations collectively demonstrated that GA-Jb could be hydroxylated by CYP512W6.

Based on the MD simulations, we identified 8 residues (V105, L108, L109, T212, S363, L364, M365, R366) that contributed more than 1.4 kcal/mol in binding affinity through MMPBSA binding free energy decomposition (Fig. 3f). Among these, V105, L108, T212, and M365 showed variability within the CYP families. Structural analyses revealed that the side chain of T212 and the main chain of M365 could form hydrogen bonds with the C3 hydroxyl and C26 carboxyl groups of GA-Jb, respectively, while V105, L108, and M365 participated in hydrophobic interactions with the tetracyclic ring of GA-Jb. Additionally, S363 formed a sulfur–hydrogen bond with the C15 hydroxyl group of GA-Jb, and R366 formed a salt bridge with the carboxyl group of heme (Fig. 3g). To identify the importance of these key residues, we designed five mutants (V105L, L108A, T212A, M365A, and R366A) to detect their C22 hydroxylation activity on GA-Jb. As expected, all mutants exhibited significantly reduced activities toward GA-Jb. The yields of 3*β*-TLTOA in the mutant strains significantly decreased to 1.0 mg/L, 0.9 mg/L, 4.2 mg/L, 0.9 mg/L, and 0.9 mg/L, respectively, compared with that of the control strain SC62-WT-r-CYP512W2-r after 120 h fermentation. This was accompanied by the massive accumulation of GA-Jb, with yields of 54.9 mg/L, 47.3 mg/L, 36.6 mg/L, 49.3 mg/L, and 61.5 mg/L, respectively (Fig. 3h). These results confirmed our predictions regarding the critical roles of these residues in stabilizing GA-Jb for C22 hydroxylation.

### Identification of GlAT as a bifunctional acetyltransferase on C15 and C22 hydroxyl groups and interpretation of its catalytic mechanism

**Fig. 4:**
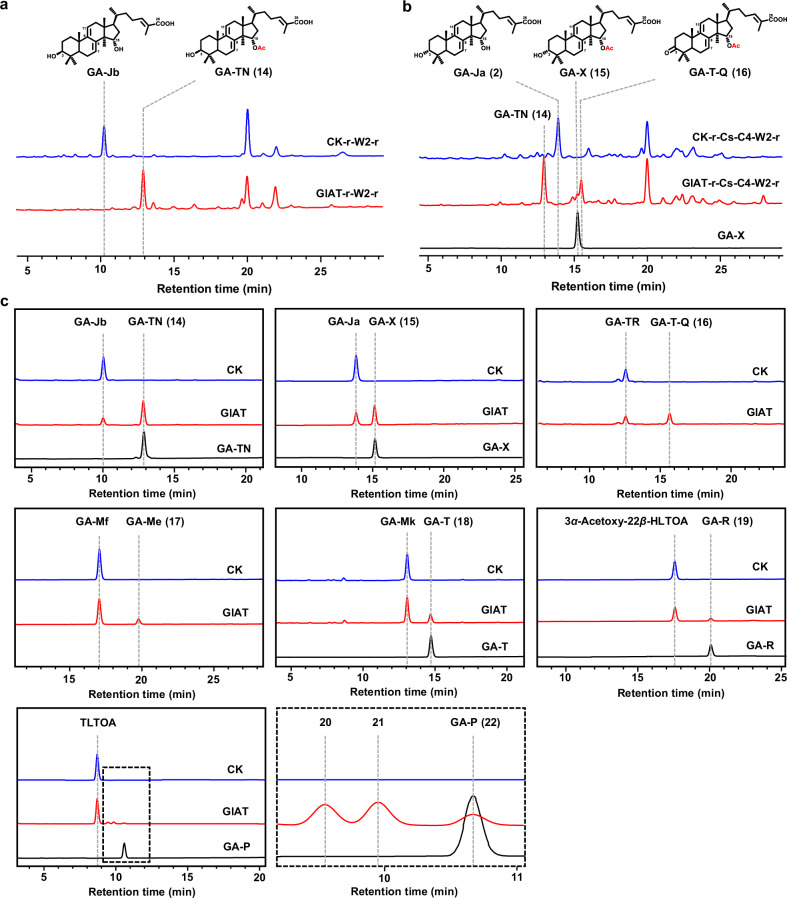
Functional characterization of GlAT. **a** HPLC analysis of the fermentation extracts of strains SC62-GlAT-r-CYP512W2-r (GlAT-r-W2-r) and SC62-CK-r-CYP512W2-r (CK-r-W2-r). **b** HPLC analysis of the fermentation extracts of strains SC62-GlAT-r-CsSDR-AKR1C4-CYP512W2-r (GlAT-r-Cs-C4-W2-r) and SC62-CK-r-CsSDR-AKR1C4-CYP512W2-r (CK-r-Cs-C4-W2-r). **c** HPLC analysis of the in vitro enzymatic extracts by incubating the GlAT-containing microsomes (prepared from the strain YL-T3-GlAT-r) with different substrates. CK indicates the microsomes prepared from the control strain YL-T3-CK-r.

To identify the missing acetyltransferases required for acetylation on C3, C15, and C22, we first introduced endogenous candidate genes (Supplementary Tables S6, S7 and Fig. S62) into the GA-Jb producing yeast. The GlAT-expressing yeast was found to generate a new product with a detected *m/z* at 495 after 120 h fermentation (Fig. 4a; Supplementary Fig. S63). An increased *m/z* of 42 compared to GA-Jb suggests that it was generated by an acetylation reaction. By scaling up the fermentation, peak **14** was further isolated and structurally characterized by NMR, confirming the presence of C15 acetyloxy and identified as GA-TN (Supplementary Table S8, Figs. S64–S69 and Note S1). When introducing GlAT into the GA-Ja production strain, the major product remained GA-TN, and two minor products with the primary *m/z* at 453 (peak **15**) and 451 (peak **16**) were detected (Fig. 4b; Supplementary Fig. S63). Peak **15** was identified as the known GA-X, the C15-acetylated product of GA-Ja, by comparison of the retention times and mass spectra with an authentic standard. As for peak **16**, it might correspond to the C15-acetylated product of GA-TR, which was identified as GA-T-Q.

**Fig. 5:**
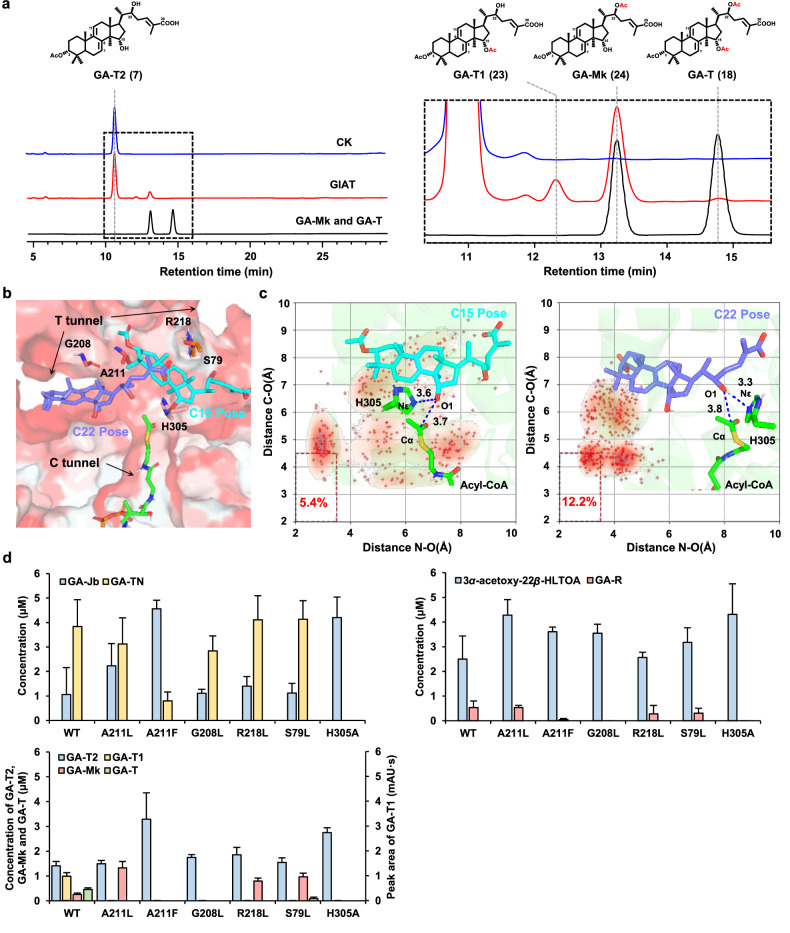
Discovery of GlAT as a bifunctional acetyltransferase on C15 and C22. **a** The extracts from the in vitro enzymatic assay of GlAT-containing microsomes with GA-T2 (left), and the enlarged view as highlighted in the black dotted line frame in the middle (right). CK indicates microsomes prepared from the control strain YL-T3-CK-r. **b** GA-T2 is docked into the T-tunnel of GlAT (shown by surface) at the C22 pose (slate) and C15 pose (cyan), respectively. **c** The percentage and represented structure at PRSs for the C15 and C22 poses, respectively. H305 and Acyl-CoA are represented by green sticks. GA-T2 in the C15 pose and C22 pose is represented by cyan and slate sticks, respectively. **d** The concentration (indicated as mg/L or mAU·s) of different substrates (indicated as light blue column) and products after 3 h incubation of strains BJ5464-GlAT-r (WT), BJ5464-A211L-r (A211L), BJ5464-A211F-r (A211F), BJ5464-G208L-r (G208L), BJ5464-R218L-r (R218L), BJ5464-S79L-r (S79L), and BJ5464-H305A-r (H305A) with respective substrates.

GlAT belongs to the membrane-bound *O*-acyl transferase (MBOAT) family, a group of membrane-anchored acetyltransferase. We prepared the GlAT-containing yeast microsomes and incubated them with different substrates. We found that it could catalyze the acetylation of the C15 hydroxyl group of a variety of substrates (Figs. 4c, 5a). When GA-Mk was used as a substrate, an LC peak with the same retention time (14.8 min) and the same primary *m/z* at 493 as GA-T was observed, indicating that an acetyl group was added to the C15 hydroxyl group (Fig. 4c; Supplementary Fig. S63). Surprisingly, when GlAT was incubated with TLTOA, three products (peaks **20**, **21**, and **22**) were observed. The MS information showed that peaks **20** and **21** corresponded to products with one acetyl group added to TLTOA (Supplementary Fig. S63). The retention time and mass spectra of peak **22** were consistent with that of GA-P, suggesting that two acetyl groups were added to TLTOA. When GA-T2 was adopted as substrate, three LC peaks (**23**, **24**, and **18**) were detected. By comparison with authentic standards, peak **24** was identified as GA-Mk (the C22-acetylated product of GA-T2), and peak **18** was identified as GA-T (the C15- and C22-acetylated product of GA-T2) (Fig. 5a; Supplementary Fig. S63). For peak **23**, with the same mass spectra as peak **24**, a later retention time as compared to GA-T2, and an earlier retention time as compared to GA-T, we inferred that it represented GA-T1, the C15-acetylated product of GA-T2. Further, 3*α*-acetoxy-22*β*-HLTOA, with one C22 hydroxyl group, was individually incubated with the GlAT-containing microsome. An LC peak with the same retention time (20.1 min) and primary *m/z* at 495 as GA-R was observed, indicating that an acetyl group was added to the C22 hydroxyl group (Fig. 4c; Supplementary Fig. S63). Taken together, these results showed that GlAT was a bifunctional acetyltransferase capable of acetylation on C15 and C22.

To test the substrate preference of GlAT, 2 mg GlAT-containing yeast microsome was incubated with 500 µM of GA-Jb, GA-Ja, TLTOA, and GA-Mf, with substrate conversion rates of 71.5%, 29.9%, 3.0%, and 15.2%, respectively (Supplementary Fig. S70a). Among all the tested substrates, GA-Jb was the most preferred, while TLTOA was the least favored. Conversion of C3*β-*hydroxyl group into the *α*-hydroxyl group (GA-Ja vs GA-Jb), oxidation at C22 (TLTOA vs GA-Ja), and C3 acetylation (GA-Mf vs GA-Ja) all exhibited reduced activities (Supplementary Fig. S70a).

To understand the catalytic mechanism of GlAT, we employed AF2 to predict its structure. ACAT1 (PDB: 6P2J), a member of the MBOAT enzyme family, shared structural similarities with GlAT, as found in the PDB database (Supplementary Fig. S71a). ACAT1 and GlAT had T- and C-tunnels for the entry of GA-Jb and acyl-CoA, respectively (Supplementary Fig. S71b, c). In addition, they possessed a catalytic histidine residue located at the intersection of these tunnels (H305 in GlAT, H460 in ACAT1). Docking results indicated that the distances between the O1 of GA-Jb and the C*α* of acyl-CoA, as well as between the Nε of H305 and O1 of GA-Jb, were both 3.1 Å, being suitable for acylation (Supplementary Fig. S71d).

To explore why GIAT could transfer acetyl groups to both C15 and C22, GA-T2 was further docked into GlAT. There are two major poses, referred to as C15- (C15 positioned near H305) and C22- (C22 positioned near H305) poses, respectively (Fig. 5b; Supplementary Fig. S71e, f). To test the stability of these two poses, three independent 100 ns MD simulations were conducted, starting from the docking structures of the C15- and C22-poses, respectively. Approximately 5.4% conformations for the C15-pose were presented as PRSs (defined as Nε-O1 and Cα-O1 less than 3.5 Å and 4.5 Å, respectively) along all 300 ns trajectories, and 12.2% for the C22-pose (Fig. 5c). These results demonstrated the capability of GlAT to transfer acyl groups at both C15 and C22 positions. Moreover, G208 and A211 around the T-tunnel were observed to provide space for GA-T2 entrance based on MD simulation results. Besides, the interaction of S79 and R218 with the tail of GA-T2 affected the acetylation position (Fig. 5b; Supplementary Fig. S71e, f). Therefore, S79L, G208L, A211L, A211F, and R218L were designed to modify the T-tunnel size or disrupt the interaction to alter the acylation site for experimental validation.

Our preliminary test demonstrated that extracellular GA-Jb can be successfully converted into GA-TN within 3 h incubation with the GlAT-overexpressing yeast strain BJ5464-GlAT-r (Supplementary Fig. S72). According to this test, the whole-cell biocatalyst-based assay of the GlAT mutants is feasible. Meanwhile, 3 h of incubation may be the appropriate reaction time for observing the conversion process, because other substrates would not be fully converted during that period due to the substrate preference. To evaluate whether the above-mentioned residues play roles in determining the substrate specificity and enzyme catalytic activity, GlAT and its mutant expression plasmids were individually introduced into the *S. cerevisiae* strain BJ5464 to generate a series of strains (Supplementary Table S7). Then, GA-Jb, 3*α*-acetoxy-22*β*-HLTOA, and GA-T2 were incubated with the corresponding strains, respectively. Consistent with our expectations, H305A mutant showed no activity towards any of the tested substrates, suggesting its crucial role in maintaining the acetylation activity on both C15 and C22 (Fig. 5d). When GA-Jb was used, a significant decrease in the production of GA-TN and accumulation of GA-Jb was observed in A211F, compared to those in the control strain BJ5464-GlAT-r, while A211L, G208L, R218L, and S79L did not show apparent differences (Fig. 5d). When 3*α*-acetoxy-22*β*-HLTOA was tested as a substrate, G208L was not able to convert it to GA-R. This outcome demonstrated that the C22 acetylation activity of G208L had been lost. Regarding A211F, it displayed reduced activity for converting 3*α*-acetoxy-22*β*-HLTOA into GA-R (Fig. 5d). Given the distinct impacts on C15 and C22 acetylation, we speculated that A211 may be another important residue for the bifunctional acetylation process, while G208 may be responsible for maintaining the C22 acetylation activity. When GA-T2 was adopted, in addition to H305A, no reaction products were observed in A211F and G208L. Due to the ambiguity in the sequence of C15 and C22 acetylation, there exist two possible pathways for the conversion of GA-T2 to GA-T by GlAT. As for G208L, it had lost its ability to produce GA-Mk, as well as to produce GA-T1 and GA-T (Fig. 5d). These results potentially suggested that when GA-T2 was used as a substrate, GlAT preferred to convert GA-T2 to form GA-Mk first, and then convert GA-Mk into GA-T. Once the C22 acetylation activity was inhibited, the favored route from C22 acetylation to C15 acetylation was blocked accordingly, leading to no detection of GA-Mk and GA-T. We also observed that G208L maintained a C15 acetylation level similar to that of the WT when reacting with GA-Jb. However, when GA-T2 was used as the substrate, G208L lost its C15 acetylation ability compared to WT, as evidenced by no detection of GA-T1 (Fig. 5d). A possible explanation is that the leucine at position 208 generates greater steric hindrance than the glycine at the same position. This difference in steric hindrance affects substrates with distinct structural features differently, resulting in different selectivities at the same chemical position of different substrates. The plenty of accumulated GA-Mk detected in A211L and R218L suggested that their C15 acetylation activity was blocked, which might be a bit contradictory to the results that they were able to convert GA-Jb into C15-acetylated GA-TN (Fig. 5d). We reasoned that the bifunctional acetylation activity may be affected by the comprehensive modifications on C3, C15, and/or C22. For residue A211, when it was mutated to leucine, the corresponding acetylation activities were detectable, but were severely reduced or diminished when it was mutated to phenylalanine (Fig. 5d). We proposed that the A211F mutation may completely block the T-tunnel, preventing substrates from accessing the catalytic site, while the A211L mutation, although it also reduces the T-tunnel space, still leaves enough room for C22 acetylation of GA-T2 when it approaches the catalytic site. However, the remaining space is insufficient for further inward movement necessary for C15 acetylation (Supplementary Fig. S73).

### Identification of heterologous BsAT as a C3 hydroxyl group acetyltransferase

**Fig. 6:**
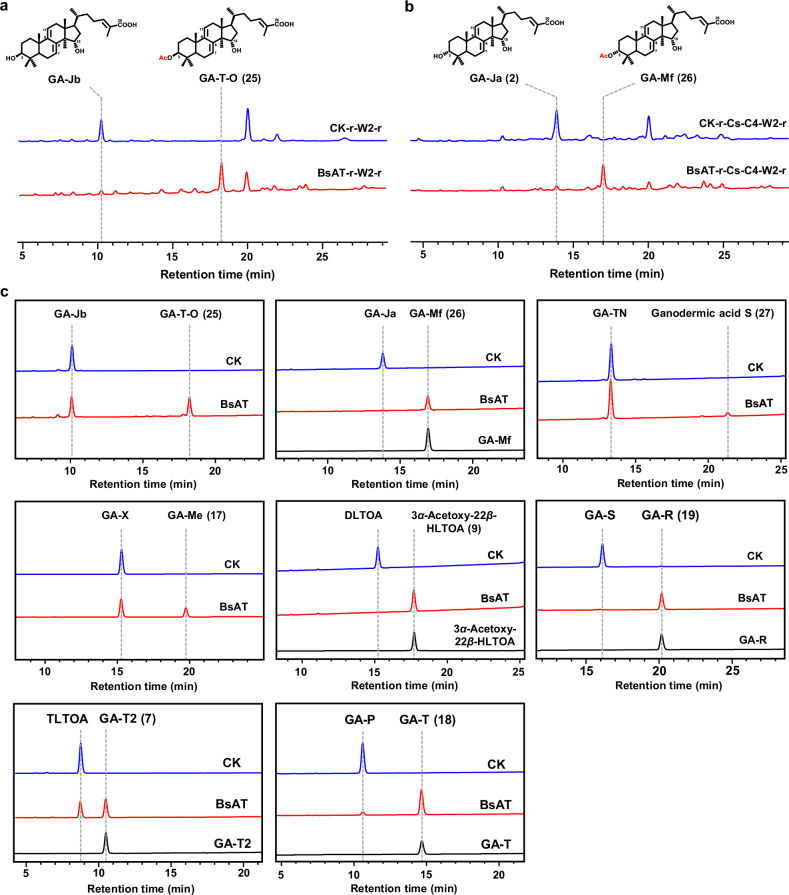
Functional characterization of BsAT. **a** HPLC analysis of the fermentation extracts of strains SC62-BsAT-r-CYP512W2-r (BsAT-r-W2-r), SC62-CK-r-CYP52W2-r (CK-r-W2-r). **b** HPLC analysis of the fermentation extracts of strains SC62-BsAT-r-CsSDR-AKR1C4-CYP512W2-r (BsAT-r-Cs-C4-W2-r) and SC62-CK-r-CsSDR-AKR1C4-CYP512W2-r (CK-r-Cs-C4-W2-r). **c** LC-MS analysis of the in vitro enzymatic reaction extracts by incubating BsAT-containing crude enzyme (prepared from the strain BL21-BsAT-r) with different substrates. CK indicates the crude enzyme prepared from the control strain BL21-CK-r.

Given the fact that no endogenous candidates were able to transfer an acetyl group to C3, we sought to test heterologous alternatives. A new compound with a detected *m/z* at 495 was found after 120 h fermentation following the introduction of BsAT, an acetyltransferase from the BAHD family in *Boswellia*^38^ (Fig. 6a; Supplementary Fig. S74). Peak **25** has a similar mass spectral pattern but different retention times compared to GA-TN, suggesting that acetylation occurs at another hydroxyl group (C3*β*-hydroxyl). To test whether BsAT can acetylate the C3*α*-hydroxyl group, it was introduced into GA-Ja producing yeast, generating a new peak with *m/z* at 435, 453 and 495 compared to the control strain (Fig. 6b; Supplementary Fig. S74). Isolation and NMR analysis of peak **26** indicated that an acetyl group was introduced into GA-Ja at C3-OH, with the chemical structure being GA-Mf (Supplementary Table S8, Figs. S75–S80 and Note S1). Therefore, we speculated that BsAT was able to acetylate different C3 hydroxyl configurations of TIIGAs.

To test the substrate broadness of BsAT, the crude enzyme of the BsAT-overexpressing *E. coli* strain BL21-BsAT-r was incubated with various substrates (Fig. 6c). When GA-Jb and GA-Ja were tested, similar to the fermentation results, BsAT catalyzed the substrates to produce peaks **25** and **26**, corresponding to GA-T-O and GA-Mf, respectively (Fig. 6a–c; Supplementary Fig. S74). When GA-TN and GA-X were tested, BsAT catalyzed the substrates to produce peaks **27** and **17**, with detected *m/z* values of 435 and 495, which may correspond to [M-2HOAc+H]^+^ and [M-HOAc+H]^+^ (Supplementary Fig. S74). These results suggested that the C3 hydroxyl group of these substrates underwent acetylation. Moreover, BsAT was catalytically active on other TIIGAs (DLTOA, GA-S, TLTOA, GA-P) regardless of whether post-modification (hydroxylation or acetylation) occurred on C15 and/or C22 positions. To further test the substrate preference of BsAT, an equivalent BsAT crude enzyme was incubated with 500 µM of GA-Jb, GA-Ja, GA-TN, GA-X, TLTOA, and GA-S, respectively, and the substrate conversion was 0.1%, 10.2%, 5.2%, 20.8%, 27.3%, and 100%, respectively (Supplementary Fig. S70b). Of all the tested substrates, GA-S was the most favored, with complete conversion. Compared to the substrates with C3*β*-hydroxyl group, BsAT had a relatively higher catalytic activity towards those with C3*α*-hydroxyl group (GA-Ja vs GA-Jb, GA-X vs GA-TN) and exhibited a higher conversion efficiency towards C15-acetylated TIIGAs and C22-hydroxylated TIIGA (GA-TN vs GA-Jb, GA-X vs GA-Ja, TLTOA vs GA-Ja).

### Reprogramming the biosynthetic network of TIIGAs

Hydroxylation is the most critical modification during GAs’ biosynthesis. The biosynthesis of GAs with more hydroxyl groups, which are a typical representative of active groups, can not only facilitate the pharmaceutical investigation on the corresponding active GAs (Supplementary Table S1) but also benefit the creation of new and promising natural products combined with other subsequent modifications, such as glycosylation and acetylation. To facilitate their commercial production, we attempted to engineer *S. cerevisiae* to stably produce these TIIGAs without adding antibiotics for enhancing the key gene expression, which was required by the plasmid-based tunable expression strategy. In our initial trial, the expression cassette of CYP512W2 was individually integrated with a single copy at H1 and H2 sites, the previously reported integration sites with enhanced target gene expression^39^. The resultant strains were able to produce GA-Jb at 2.0 mg/L (for H1 site integration) and 1.7 mg/L (for H2 site integration), while their accumulated HLDOA reached 65.1 mg/L and 38.7 mg/L, respectively (Supplementary Fig. S81).

**Fig. 7:**
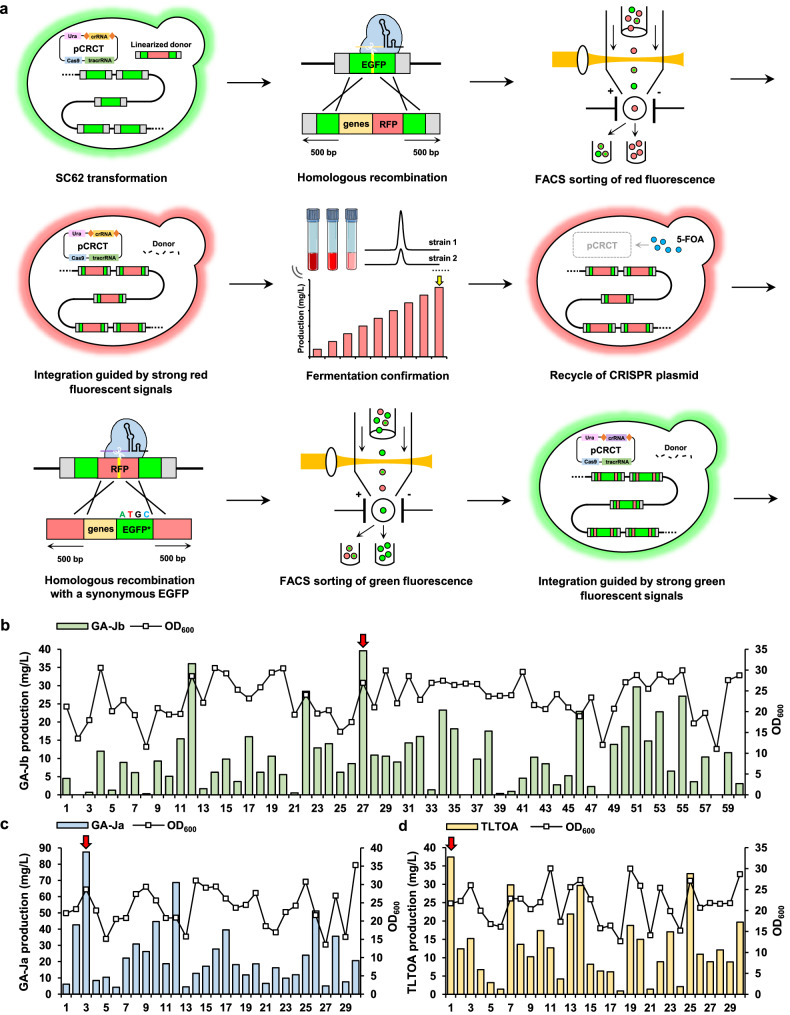
Integration of key enzyme expression cassettes leads to efficient production of significant TIIGAs. **a** The principle of integration. RFP and key enzyme expression cassettes, flanked by 500 bp homologous recombination arms, are used as donors for integration at the EGFP locus of strain SC62. Recombinant clones with high RFP but no GFP fluorescent signal are collected by FACS. Test tube fermentation is performed to screen strains with high production of TIIGAs. Then, the CRISPR plasmid is recycled by culturing in 5-FOA containing medium. The resultant strain is used for integration of other enzyme expression cassettes. For this round of integration, a synonymous mutant EGFP is designed in the donor to avoid its own homologous recombination with EGFP locus of SC62. **b**–**d** Production of GA-Jb (**b**), GA-Ja (**c**), and TLTOA (**d**) by selected clones after 120 h fermentation. The colonies with the highest TIIGA production are indicated by the red arrows.

**Fig. 8:**
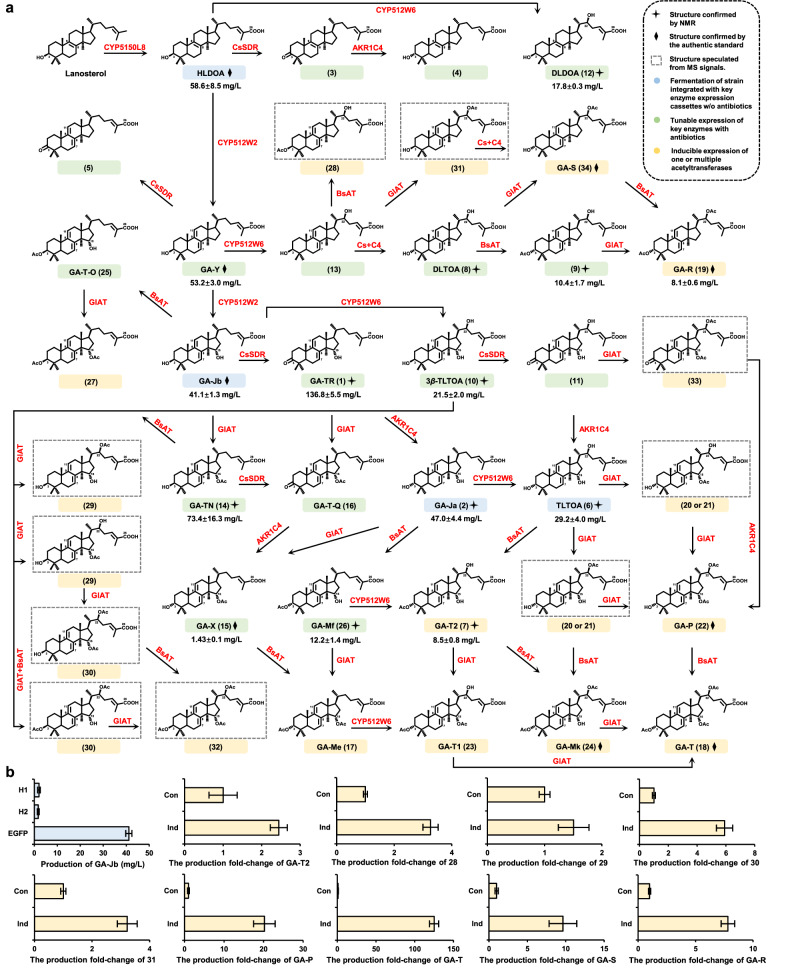
Reprogramming of the biosynthetic network to target TIIGAs. **a** Biosynthesis of target TIIGAs can be achieved by tunable expression of key enzymes either via changing the concentration of antibiotics (green), integration of key enzyme expression cassettes without antibiotics (blue), or inducible expression of acetyltransferases (yellow). For speculating the chemical structures corresponding to peaks **20**, **21**, **28**–**33**, see Supplementary Figs. S84–S86 and Note S2. **b** Production of TIIGAs by constitutive and inducible expression of acetyltransferases, and by integration of key enzyme expression cassettes at different loci (H1, H2, EGFP and RFP). Cs+C4, CsSDR and AKR1C4; Con, constitutive; Ind, inducible expression. All data represent the mean of three independent samples, and the error bars indicate the standard deviation.

Inspired by our previous work^21^, we continuously integrated the expression cassette of CYP512W2^I108A^ mutant^37^, with enhanced C15 selectivity for generating GA-Jb, together with a red fluorescent protein (RFP) expression cassette, at the EGFP multicopy loci. We selected two guide sequences targeting 122 bp and 309 bp downstream of the EGFP start codon for integration and screened them by fluorescence-activated cell sorting (FACS) (Fig. 7a). The percentage of EGFP-positive cells was 3.6% and 1.5%, respectively. In contrast, the EGFP-positive cells in the initial strain SC62 were 99.1% (Supplementary Fig. S82). The significantly reduced EGFP signals demonstrated that the EGFP was efficiently disrupted when adopting different spacers. However, the percentage of RFP-positive cells was only ~0.1% in both cases. We attributed this to the insensitive detection for RFP, since the RFP-overexpressing cells only exhibited 15.0% positive signal (Supplementary Fig. S82). For integrating at different loci of EGFP, we collected ~8000 cells with the strongest red fluorescence intensity and no green fluorescence. Strain SC27 was able to produce the highest amount of GA-Jb at 39.6 mg/L without adding antibiotics (Fig. 7b). It was further subjected to cultivation in YPD medium containing 1 g/L 5-FOA to recycle the CRISPR plasmid. The final resultant strain SC27* was able to produce GA-Jb at 41.1 mg/L after 120 h fermentation (Fig. 8).

Then, the GA-Jb producing strain SC27* was engineered to produce GA-Ja and TLTOA. The expression cassettes of CsSDR and AKRC14 (responsible for the configuration conversion of the C3 hydroxyl group), or CsSDR, AKRC4 and CYP512W6 (responsible for the configuration conversion of the C3 hydroxyl group and C22 hydroxylation), together with a synonymous mutated EGFP for avoiding partial homologous recombination with the integrated EGFP cassettes, were designed to be integrated into the RFP loci of SC27*. We selected a guide sequence targeting 191 bp downstream of the RFP start codon for donor integration. The percentages of EGFP-positive cells were restored to 63.4% and 57.2%, suggesting the efficient integration of these donors (Supplementary Fig. S82). We collected ~10,000 cells with the strongest green fluorescence intensity and no red fluorescence. Strains SC3 and SC1 were able to produce the highest amounts of GA-Ja (87.5 mg/L) and TLTOA (37.4 mg/L) after 120 h fermentation, respectively, without antibiotic addition (Figs. 7c, d and 8). After plasmid recycle, the final resultant strains SC3* and SC1* were able to produce 47.0 mg/L of GA-Ja and 29.2 mg/L of TLTOA after 120 h of fermentation, respectively.

To achieve heterologous biosynthesis of TIIGAs with more complicated post-modifications, the genes of *cyp512w6*, *glat*, and *bsat*, driven by constitutive promoters (P_*HXT7*_, P_*PYK1*_, P_*TPI1*_), were introduced into the GA-Jb and GA-Ja producing strains. However, once GlAT was introduced, GA-TN became the overwhelming metabolite in all the engineered strains (Supplementary Fig. S83). With a strong substrate preference, GlAT competed with other enzymes for GA-Jb to generate GA-TN, which may lead to the arrest of biosynthetic networks. Even if GlAT was excluded, the strain co-expressing CYP512W2, CsSDR, AKR1C4, CYP512W6 and BsAT was not able to generate the final product GA-T2 efficiently (Supplementary Fig. S83). Accompanying the large accumulation of upstream products, we detected 22.9 mg/L of TLTOA, 9.8 mg/L of GA-Mf, and 10.4 mg/L of 3*α*-acetoxy-22*β*-HLTOA. The fermentation results indicated that the post-modification enzymes consumed substrates in a competitive manner and their substrate preference for upstream GAs of both GlAT and BsAT led to the inefficient production of downstream GAs.

Considering the complexity of the enzymes in TIIGA synthetic network, we sought to control the order of post-modification reactions by using inducible promoters to drive the expression of BsAT and/or GlAT^40^. Firstly, we attempted to temporally regulate BsAT expression to unblock metabolism from TLTOA and GA-Mf to GA-T2 in SC1*. Galactose-inducible promoter P_*GAL1*_ was selected to drive the gene expression. After 72 h fermentation in YPD24 medium and 96 h induction in YPG24 medium, the strain was able to produce 3.1 times more GA-T2 and accumulate 45.3% less TLTOA and 28.3% less GA-Mf compared to those of the constitutively expressed strain after 168 h fermentation in YPD24 medium (Fig. 8b; Supplementary Fig. S84 and Table S2). In addition, compound **28** was accumulated after induction, with a 3.3-fold increase compared to the control strain. These results demonstrated the successful rewiring of metabolic flux towards GA-T2.

*S. cerevisiae* produces ethanol in aerobic condition, and then consumes it as carbon source to promote cell growth and acetic acid production. Therefore, we adopted P_*ADH2*_ (ethanol-inducible promoter) and P_*GAL1*_ to drive the expression of two acetyltransferases in SC27*. For the production of C3 configuration unconverted TIIGAs, the different induction sequence of GlAT and BsAT led to alternations in the production patterns of TIIGAs (Supplementary Fig. S85). In the strain SC27-P_*ADH2*_BsAT-CYP512W6-P_*GAL1*_GlAT-r, the accumulation of GA-TN was reduced by 81.2%. After 168 h of fermentation, it was able to produce 1.5- and 3.6-fold more of compounds **29** and **30**, respectively. Notably, compared to the control strain, not only was there an increased production of acetylated TIIGAs, but peak **32** was also observed after induction. Meanwhile, in the strain SC27-P_*ADH2*_GlAT-CYP512W6-P_*GAL1*_BsAT-r, the accumulation of GA-TN was decreased by 68.8%. This strain could produce 5.9- and 3.2-fold more of compounds **30** and **31**, respectively (Supplementary Table S2). For production of C3 configuration converted TIIGAs, the dual inducible expression of GlAT and BsAT expedited the metabolic flux towards downstream TIIGAs. With 91.8% reduced GA-TN accumulation, the strain SC27-P_*ADH2*_BsAT-CYP512W6-P_*GAL1*_GlAT-r-CsSDR-AKR1C4-r was able to produce 7.8- and 125.1-fold more of GA-R and GA-T, respectively, compared to those from the control strain adopting the constitutive promoter for the expression of GlAT and BsAT after 168 h fermentation (Fig. 8b; Supplementary Fig. S86). Notably, GA-Mk, Ganodermic acid S (GMA-S) and compound **33**, which were not detected in the control strains, were also observed after induction (Supplementary Fig. S86 and Table S2). Meanwhile, the induced strain also accumulated a certain amount of TLTOA and GA-Mf. With 47.3% reduced GA-TN accumulation, the strain SC27-P_*ADH2*_GlAT-CYP512W6-P_*GAL1*_BsAT-r-CsSDR-AKR1C4-r was able to produce 9.6- and 20.2-fold more of GA-S and GA-P, respectively, compared to the control strain after induction. In addition, the strain also produced GA-T1, compound **33** and the C3 configuration unconverted TIIGA **30**, which were not detected in the control strain (Fig. 8b; Supplementary Fig. S86 and Table S2).

By harnessing the power of synthetic biology, we have successfully achieved the production of several upstream GAs (e.g., HLDOA, GA-Y, and GA-Jb) by engineered yeasts with titers around 2–150 mg/L, the increased production of which heavily relies on the addition of antibiotics in fermentation cultures^21,37,41,42^. According to previous reports as summarized in Supplementary Table S2, the downstream TIIGAs were predominately obtained by liquid fermentation of *G. lucidum* mycelia or from the planted fruiting bodies during 1–4 months, with yields at 3.38 × 10^–4^–16.17 mg/g DW or 0.10–206.40 mg/L and productivity at 6.27 × 10^–5^–0.60 mg/g-DW/d or 1.62 × 10^–3^–6.07 mg/L/d. In our study, the production efficiencies or yields of over 30 TIIGAs by the engineered yeast strains were 1–4 orders of magnitude higher than those from farmed mushrooms (Fig. 8; Supplementary Table S2). In addition to specific target TIIGAs, the yeast cell can be reprogrammed to produce multiple TIIGAs with comparable yields, and more interestingly, to biosynthesize TIIGAs that cannot be obtained from their native host (Fig. 8; Supplementary Table S2).

## Discussion

As an edible and medicinal mushroom, *G. lucidum* can meet various human nutritional and healthcare needs. This is attributed to the dosage and composition of specific active metabolites, TIIGAs. Although TIIGAs show significant accumulation during the early developmental stage (mycelia) of *G. lucidum*, the expression level of the key missing C22 hydroxylase CYP512W6 (GL23335) remains relatively constant across different developmental stages (mycelia, primordia, and fruiting bodies)^43^. This suggests that prioritizing enzyme candidates based on expression profiles across different developmental stages and tissues, a commonly used enzyme discovery method in many other studies^44,45^, might not be applicable in this case. Therefore, we employed multiple strategies to enhance TIIGA production during the mycelia stage. Without these strategies, the key missing enzymes could not have been identified. Additionally, a series of C22-hydroxylated GAs cannot be chemically synthesized as standards to guide enzyme discovery due to the low yield of the template TIIGAs obtained from mushroom cultivation.

We discovered the C22 hydroxylase CYP512W6 and elucidated its catalytic mechanism. It is worth noting that in our previous work, we also attempted to express CYP512W6, yet no new product was detected. Using primer pairs with high sequence similarity to those previously reported, we obtained two “CYP512W6s” with entirely different coding sequences by using different cDNA templates under shake-static cultivation and blue light stimulation. These results clearly demonstrated that the gene encoding the key missing C22 hydroxylase can be captured under more sophisticated culture conditions. In fact, several studies have reported that CYPs catalyze post-modifications (hydroxylation, oxidation, and dehydrogenation) at the C22 site of GA-similar substrates in plants and fungi. Among them, most C22 hydroxylases belong to the CYP90 family^46–49^. However, none of these heterologous candidates were able to catalyze hydroxylation at the C22 site of the GA backbone, highlighting the uniqueness and irreplaceability of CYP512W6 in GA hydroxylation. The mutation assay has validated that the non-conserved residues V105 and M365 may be crucial for maintaining the catalytic activity of CYP512W6.

The successful identification of GlAT as a bifunctional acetyltransferase is a significant discovery. To our knowledge, characterized triterpenoid or steroid acetyltransferases are only capable of transferring acetyl groups on C3, C4, C6, C9, C16, C21, or C24^50^ (Supplementary Table S5). Neither a C15 nor a C22 acetyltransferase of terpenoids or substrates with similar chemical structures have been identified, let alone such a bifunctional acetyltransferase. Although multi-functional acetyltransferases acting on adjacent carbon atoms on the same glycone have been constantly reported^51^, the ability of a single enzyme to catalyze acetylation at both C15 and C22 of triterpenoid skeleton, with a relatively long distance between the hydroxyl groups being modified, was previously considered improbable.

The biosynthesis of GAs forms a complex network with high flexibility in post-modification sequences. Cross-reactions resulting from the broad substrate specificity of enzymes can lead to low conversion efficiency and the accumulation of by-products. We reprogrammed the biosynthetic network to target promising compounds by inducing the key downstream enzymes at the appropriate time and in the proper sequence. Using the platform established in this work, we achieved de novo biosynthesis of over 30 bioactive TIIGAs, including three unreported compounds, and the crucial skeleton compound (TLTOA) for generating TIIGA derivatives (Supplementary Table S1). More than five TIIGAs showed a 10-fold increase in production titers (Supplementary Table S2). This achievement enables the development of functional foods and the in-depth pharmacological studies of mushroom-specified bioactive ingredients.

In this systematic study, we identified the missing steps in the synthetic network of TIIGAs. We demonstrated that by appropriately combing the identified enzymes and regulating their expression, directed and efficient biosynthesis of targeted TIIGAs can be achieved in engineered yeast strains. Beyond the identified TIIGAs, multiple TIIGAs that may not be isolable in the native host due to rapid conversion can also be biosynthesized through this guided network. Additionally, by combining multiple computation-aided designs with experimental verification, we identified key residues critical for catalytic activity and substrate selectivity, providing insights for subsequent protein engineering. With a deeper understanding of the catalytic mechanisms of these enzymes, systematic protein engineering is expected to be employed in future studies to generate mutants that can directly redirect the metabolic flux towards end-product TIIGAs. Such efforts include, but are not limited to, enhancing the catalytic activity of CYP512W6 on GA-Jb, improving the substrate selectivity of GlAT and BsAT towards TLTOA, and designing novel transferases capable of transferring acetyl groups to C3, C15 and C22. Moreover, the targeted construction of yeast communities can be considered to reduce intracellular substrate admixture through inter-community metabolite exchange, thereby achieving efficient production of target compounds. For example, an upstream yeast community could specialize in the production of TLTOA, while a downstream community could solely express acetyltransferases, avoiding cross-reactivity and flux fragmentation caused by networked synthesis pathways.

In conclusion, this study enables access to the active ingredients TIIGAs from *G. lucidum*. It also provides a solution for accelerating the discovery and engineering of the biosynthesis of active ingredients from medicinal mushrooms.

## Materials and methods

### *G. lucidum* cultivation and metabolite extraction

The *G. lucidum* strain CGMCC (China General Microbiological Culture Collection Center, Beijing, China) 5.616 was maintained on potato-agar-dextrose slant. Pre-cultivation and two-stage shake-static fermentation were performed at 30 °C^52,53^. Shake-flask fermentation was performed in the dark for 2 days, followed by the static-plate fermentation in the dark or with 100 lux blue light exposure for an additional 10 days. Mycelia were collected at 2, 8, and 12 days of fermentation to detect the accumulation of GAs and extract total RNA for RNA sequencing. After being washed three times with distilled water, one-quarter of the mycelia were immediately frozen in liquid nitrogen and stored at –80 °C for RNA extraction. The remaining mycelia were dried to a constant weight at 50 °C in a ventilated oven (Jinghong, Shanghai, China).

The dried mycelia were homogenized into powder using stainless-steel beads in a tissue grinder (Jingxin, Shanghai, China) at 50 Hz for 5 min. Twenty-five mg of mycelium powder were extracted three times with 1 mL of ethyl acetate by 30 min sonication. After centrifugation (25 °C, 12,000× *g*, 10 min), the supernatants were collected, dried under vacuum at 45 °C, resuspended in 500 μL of methanol, filtered through a 0.22 μm PTFE syringe filter (DIKMA, Shanghai, China), and then subjected to HPLC analysis.

### Yeast microsome isolation and in vitro enzymatic assays

Yeast strains CK-r-iGLCPR-r, YL-T3-GlAT-r and control strains CYP512W6-r-iGLCPR-r, YL-T3-CK-r were used for microsome preparation. The strains were grown in the corresponding liquid medium (SC-HLU or SC-HU medium) for 24 h and transferred to 200 mL of YPD24 medium containing 300 mg/L of G418 at an initial OD_600_ of 0.05. After 60 h of fermentation, yeast cells were collected for microsome isolation^20^. For CYP512W6, 2 mg of microsomal proteins, 2 mM of NADPH, and 400–600 μM of GAs were incubated at 30 °C and 140 rpm for 12 h in the in vitro enzymatic reaction. For GlAT, the in vitro enzymatic reactions were performed in 500 μL of 90 mM Tris-HCl (pH 7.5) containing 2 mg of microsomal protein, 2 mM acetyl CoA, and excessive GAs. After incubation at 30 °C and 140 rpm for 24 h, the products were extracted with ethyl acetate and subjected to HPLC and UPLC-MS analyses. For testing the substrate preference of GlAT, 500 μM of GAs and 12 h incubation time were adopted.

### Construction of protein models and MD stimulations

The structures of CYP512W6 and GlAT were predicted using AF2, and their protonation states were determined via the PDB2PQR web server. The structure of CYP512W2 was used to determine the position of the heme group in CYP512W6. The complex structures (CYP512W2 with GA-Jb, GlAT with GA-Jb, and GlAT with GA-T2) were generated using AutoDock. The parameters for GA-Jb and GA-T2 were generated with the Antechamber module. Cpd I and its covalently bonded axial Cys were assigned amber-compatible parameters as developed by Cheatham et al.^54^.

The complexes of GA-Jb with CYP512W2 and GlAT with GA-T2 were immersed in a TIP3P water box with a 12 Å buffer using the Leap module. Three independent MD simulation replicas were performed using pmemd.cuda in Amber18 for each system. Initially, 5000 cycles of minimization were conducted using the conjugate gradient method, allowing solvent and hydrogen atoms to move, followed by another 5000 cycles with no restraints. The systems were then heated from 0 K to 300 K. Subsequently, a 200-ps equilibration simulation was carried out under constant pressure and temperature. Finally, three 100 ns simulations were performed, employing the SHAKE algorithm to constrain the motion of water molecules and the particle-mesh Ewald (PME) method to account for long-range electrostatic interactions.

### QM calculations

The truncated QM model containing Cpd I and GA-Jb was derived from a representative snapshot of the MD simulation. Geometry optimization and transition states were calculated at the B3LYP level with LANL2DZ for iron and 6-31 G(d) for the remaining atoms by Gaussian 16. The structures were drawn using CYLview.

### *E. coli* crude enzyme preparation and in vitro enzymatic assays

Colonies of *E. coli* strain BL21-BsAT-r and control strain BL21-CK-r were picked and incubated overnight at 37 °C and 220 rpm in 12 mL test tubes containing 4 mL of LB liquid medium and 50 mg/L of kanamycin. They were then transferred to 1 L shake flasks containing 200 mL of LB liquid medium and 50 mg/L of kanamycin at 1% v/v for 3–4 h of incubation. When the OD_600_ reached 0.6, 0.2 mM IPTG was added for overnight incubation at 16 °C. After induction, the cells were centrifugate (3000× *g*, 10 min, 4 °C) and washed twice with phosphate-buffered saline (PBS, pH 7.4). After centrifugation (3000× *g*, 10 min, 4 °C) again, the cell precipitate was suspended in 50 mL of purification buffer (10% glycerol, 20 mM Tris-HCl, and 200 mM NaCl, pH 7.4) and ultrasonicated for 15 min at 4 °C (sonication for 15 s with a 10 s interval). The supernatant obtained by centrifugation (10,000× *g*, 10 min, 4 °C) was the crude enzyme of *E. coli*. The in vitro enzymatic reactions were performed in 1 mL of 50 mM Tris-HCl buffer (pH 7.4) containing 2 mg of crude enzyme, 2 mM of acetyl CoA, and moderate amount of GA. After incubation at 30 °C and 140 rpm for 24 h, the products were extracted with ethyl acetate and subjected to HPLC and UPLC-MS analyses. For testing the substrate preference of BsAT, 1 mg of crude enzyme, 500 μM of GAs and 12 h incubation time were adopted.

### Analytical methods

Cell growth was determined by measuring OD_600_ using a BioPhotometer (Eppendorf, Hamburg, Germany). Metabolite concentrations were determined using an Agilent XDB-C18 column (4.6 μm, 5 mm × 250 mm) on Agilent 1200 HPLC system. Mobile phase A was ultrapure water, and mobile phase B was methanol/acetic acid (100:0.1 v/v). The samples were eluted from the column in a linear gradient of 80%–100% B in 30 min at a flow rate of 1 mL/min. LC-MS was performed using an UPLC system (Waters, Wilmslow, UK), connected to a Q-TOF MS in atmospheric pressure chemical ionization (APCI) positive ion mode, with a Waters BEH C18 column (1.7 µm, 2.1 mm × 100 mm)^55^. The purified compounds were dissolved in deuterated methanol (**2**, **6**, **7**, **8**, **9**, **12**, **10**, and **26**) or deuterated chloroform (**1** and **14**) and subjected to NMR analysis on an Avance III 600 MHz Nuclear Magnetic Resonance instrument (Bruker, Karlsruhe, Germany). Chemical shifts (*δ*) were expressed in parts per million (ppm), and coupling constants (*J*) were expressed in hertz (Hz). The NMR data were analyzed using MestReNova (version 14.0.0).

Detailed materials and methods for all experiments described in this study are provided in the Supplementary materials and methods section.

## Supplementary information

Supplementary information

Supplementary Table S3

Supplementary Table S4

Supplementary Table S5

Supplementary Table S6

Supplementary Table S7

Supplementary Table S8
