# Supplementary material for: Decoding and reprogramming of the biosynthetic networks of mushroom-derived bioactive type II ganoderic acids in yeast
Source: Cell Discov. 2025 Jul 8;11:61. doi: 10.1038/s41421-025-00812-1 (PMC12234687; doi:10.1038/s41421-025-00812-1)
Supplement: Supplementary file 1 — Supplementary information [file 41421_2025_812_MOESM1_ESM.pdf]

## Supplementary information

# Decoding and reprogramming of the biosynthetic networks of mushroom derived bioactive type II ganoderic acids in yeast

Qin Wang [1], Ye Li [1], Shunhan Zhang [1], Wei Yuan [2], Zeqian Du [1], Ting Shi [1], Zhao Chang [1, 3], Xingye Zhai [1], Yinhua Lu [3], Meng Wang [2], Juan Guo [4], Jian-Jiang Zhong [1], Han Xiao [1, *]

[1] State Key Laboratory of Microbial Metabolism, Joint International Research Laboratory of Metabolic & Developmental Sciences, School of Life Sciences and Biotechnology, Shanghai Jiao Tong University, 800 Dong-chuan Road, Shanghai, 200240, China

[2] Key Laboratory of Engineering Biology for Low-Carbon Manufacturing, Tianjin 300308, China.

[3] College of Life Sciences, Shanghai Normal University, Shanghai 200234, China

[4] State Key Laboratory for Quality Ensurance and Sustainable Use of Dao-di Herbs, National Resource Center for Chinese Materia Medica, China Academy of Chinese Medical Science, Beijing, 100700, China

[*] Corresponding author: Xiao, H. (smallhan@sjtu.edu.cn)

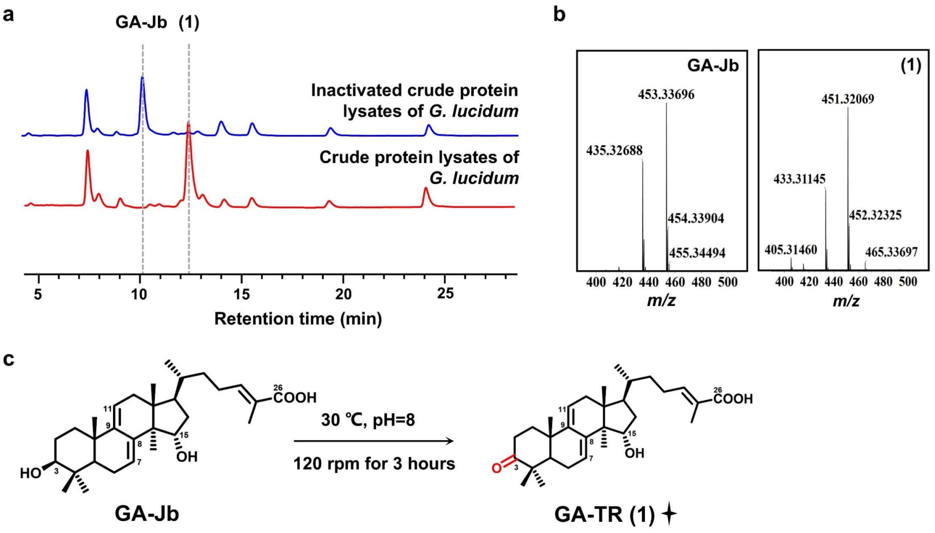


**Supplementary Fig. S1: *In vitro* activity assay demonstrates C3 oxidation**. **a.** HPLC analysis of extracts from the *in vitro* enzymatic reactions. The crude protein lysates of *G. lucidum*, along with the heat-inactivated crude enzyme, were each incubated with GA-Jb, respectively. **b.** Mass spectra of GA-Jb and peak **1**. **c.** Reaction catalyzed by the crude enzyme of *G. lucidum*. The compound marked with a star indicates that its chemical structure has been confirmed by NMR analyses.

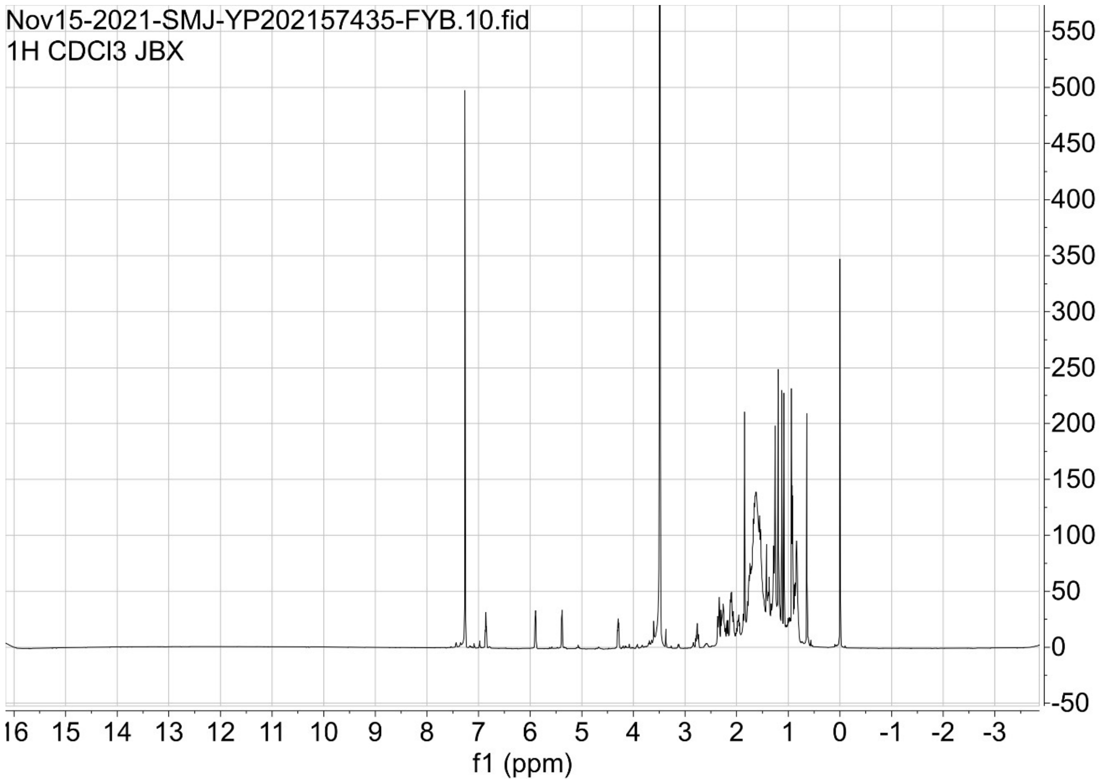


**Supplementary Fig. S2: $^{1}$H NMR spectrum of 1.**

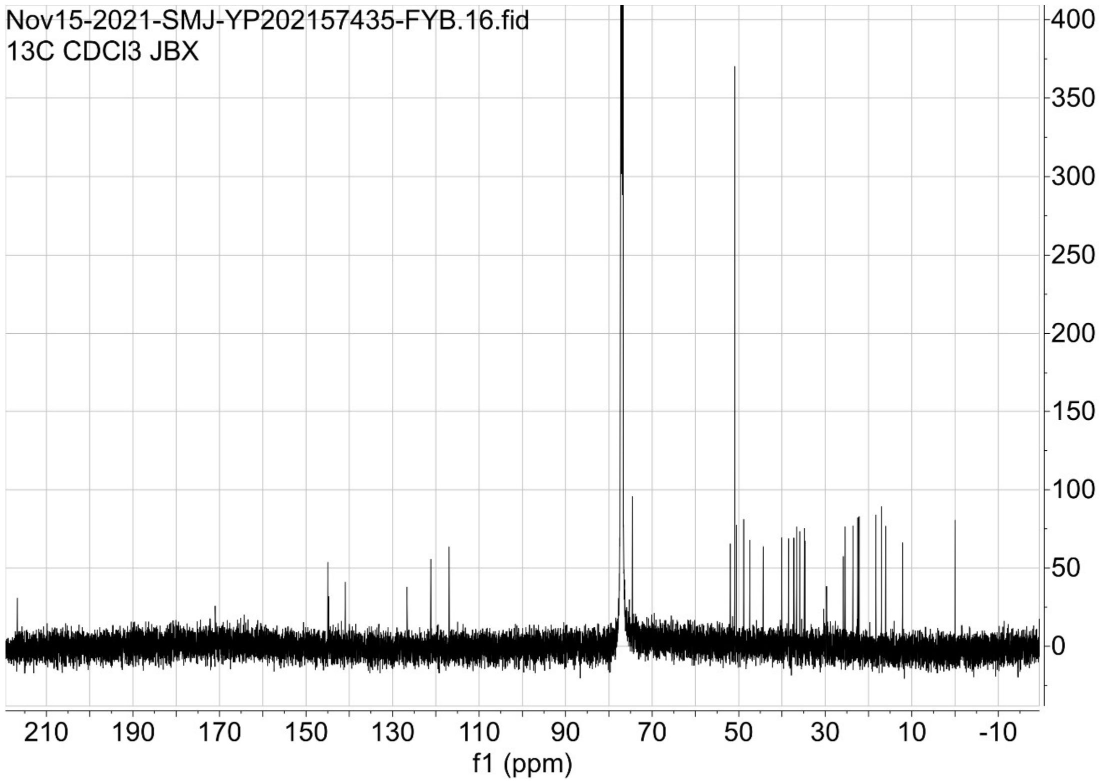


**Supplementary Fig. S3: $^{13}$C NMR spectrum of 1.**

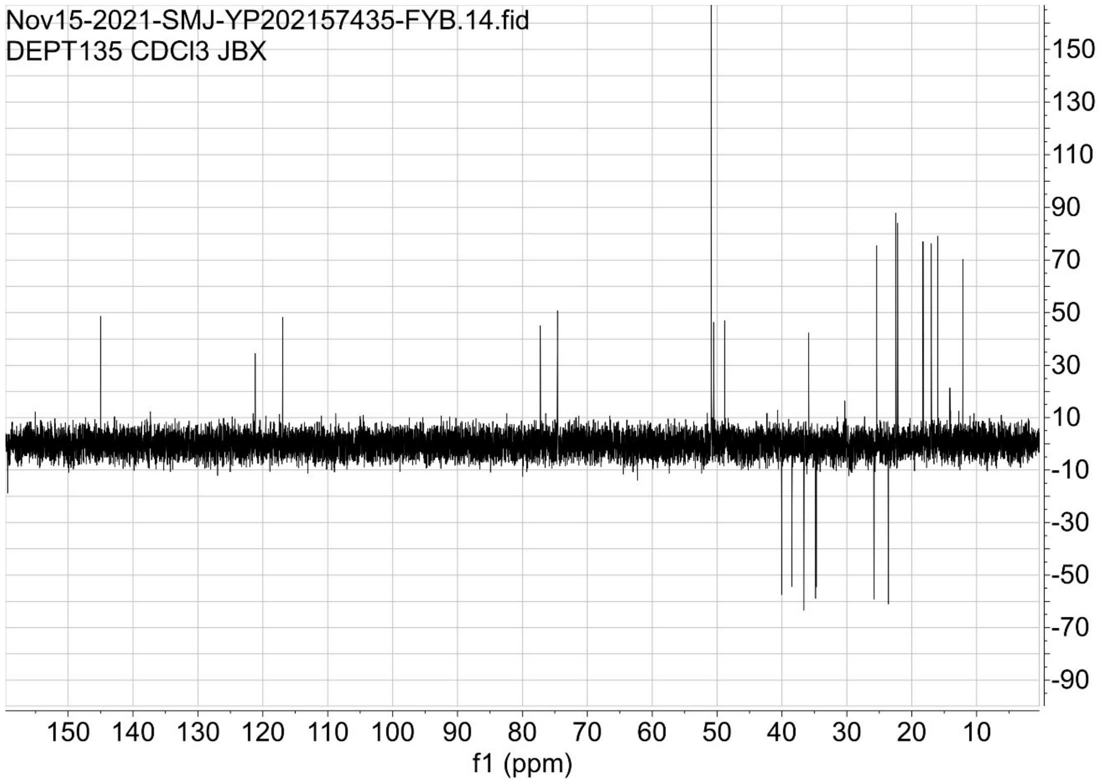


**Supplementary Fig. S4: DEPT-135 spectrum of 1.**

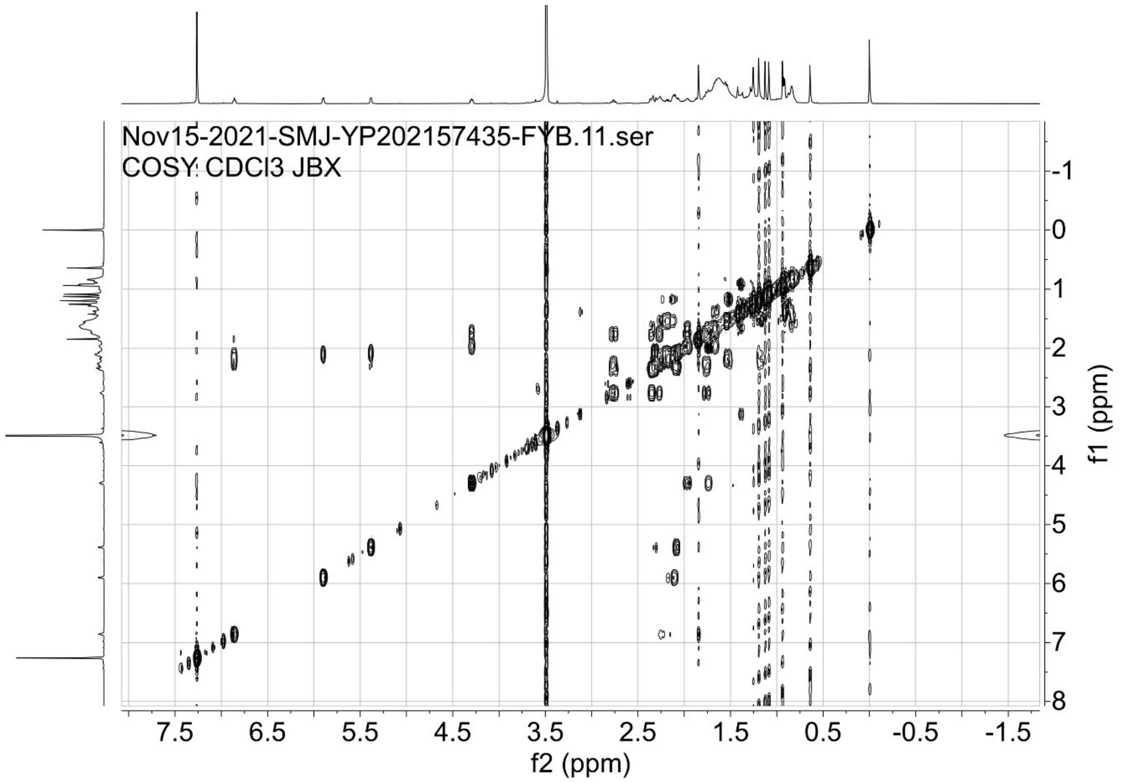


**Supplementary Fig. S5: COSY spectrum of 1.**

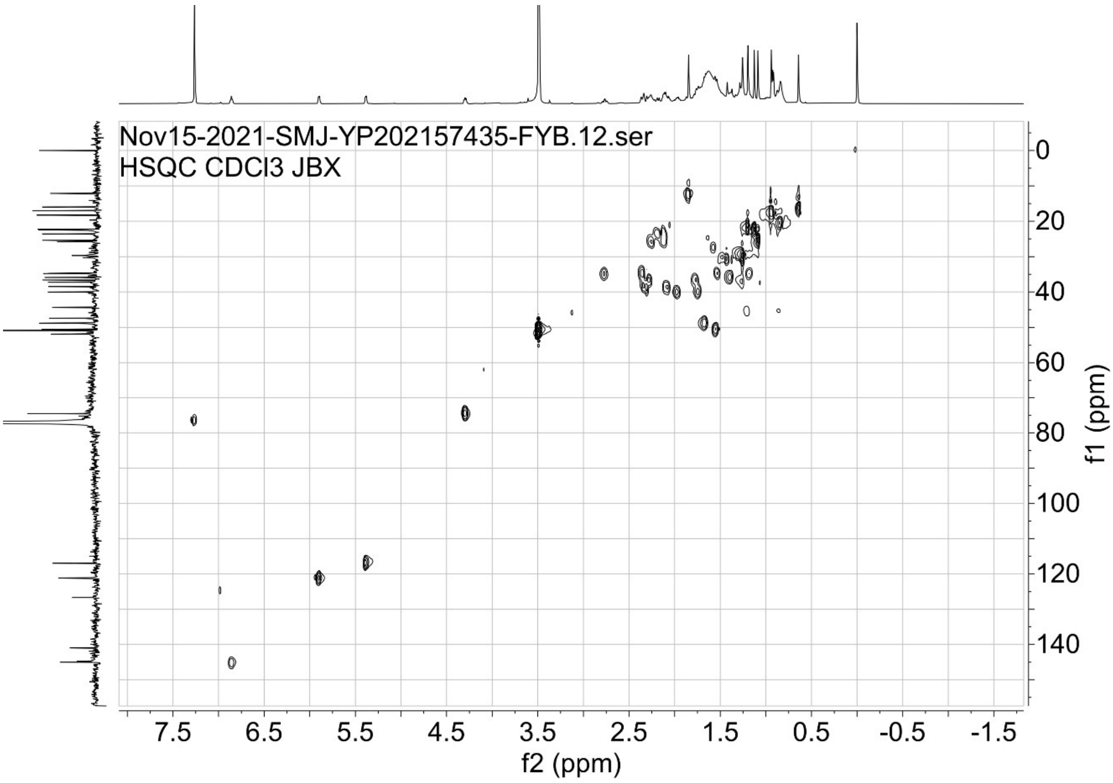


**Supplementary Fig. S6: HSQC spectrum of 1.**

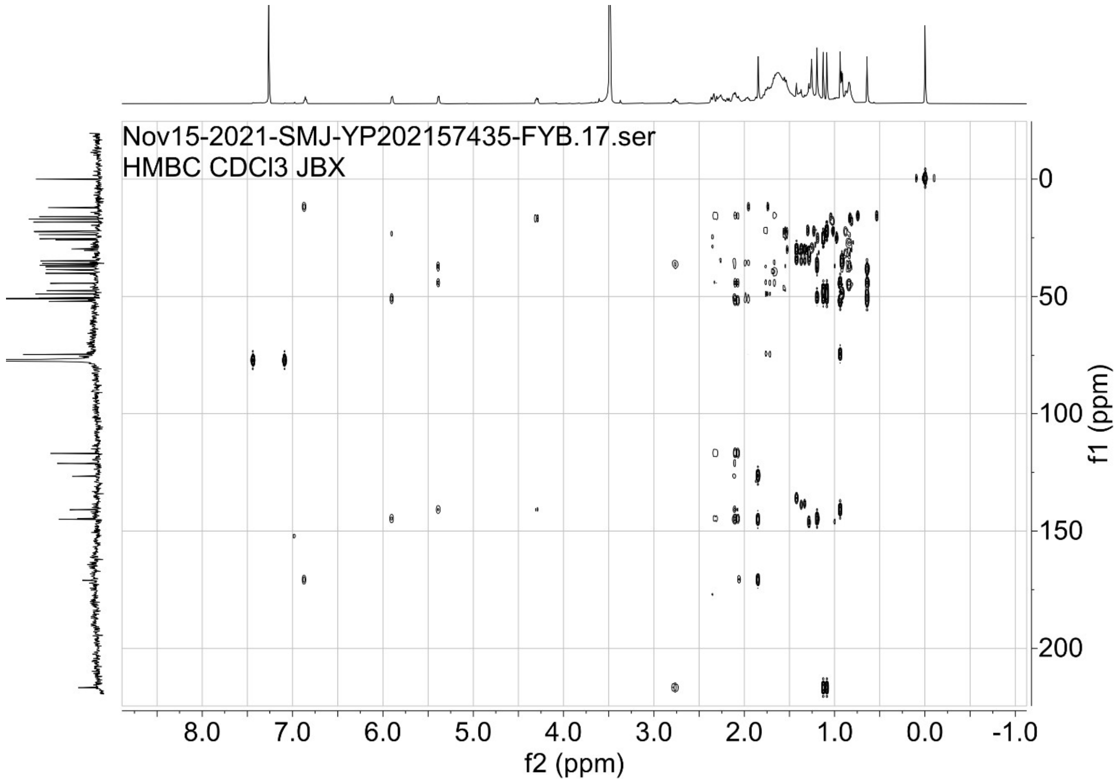


**Supplementary Fig. S7: HMBC spectrum of 1.**

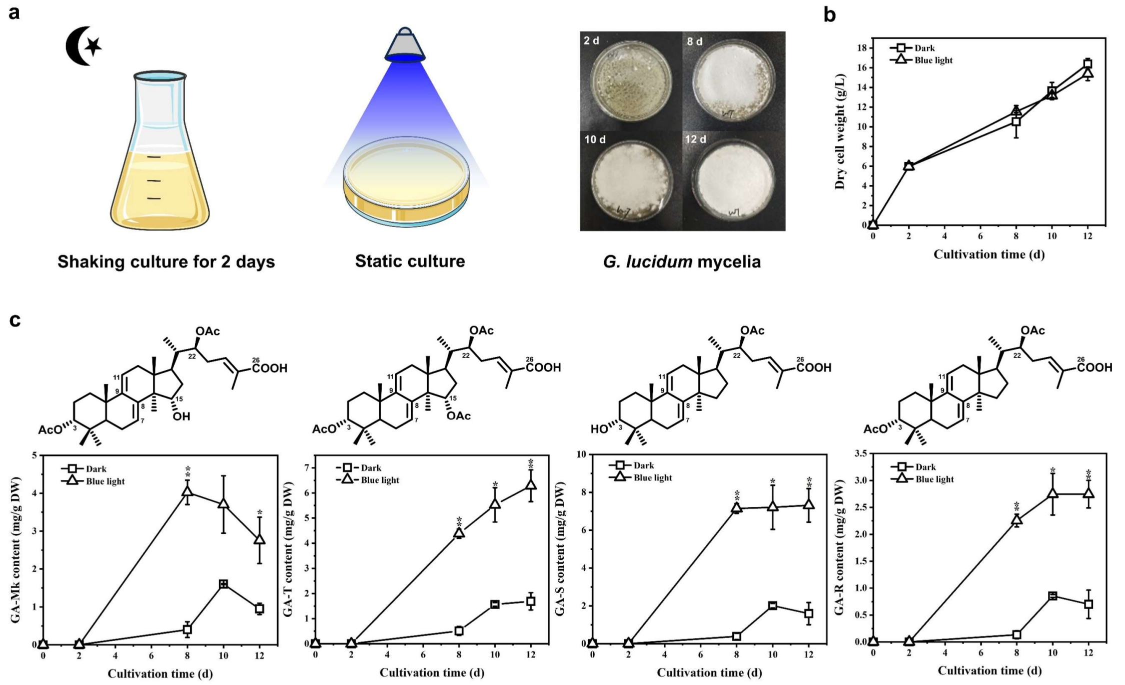


**Supplementary Fig. S8: Blue light exposure promotes the accumulation of multiple TIIGAs in *G. lucidum*. a.** Cultivation of *G. lucidum* under blue light exposure. **b.** Dry cell weight of *G. lucidum*. **c.** Contents of GA-Mk, GA-T, GA-S, and GA-R. All data are represented as the mean of three independent samples. The error bars represent the standard deviation. Statistical comparisons were carried out using a two-tailed Student's t-test, *P<0.05, **P<0.01.

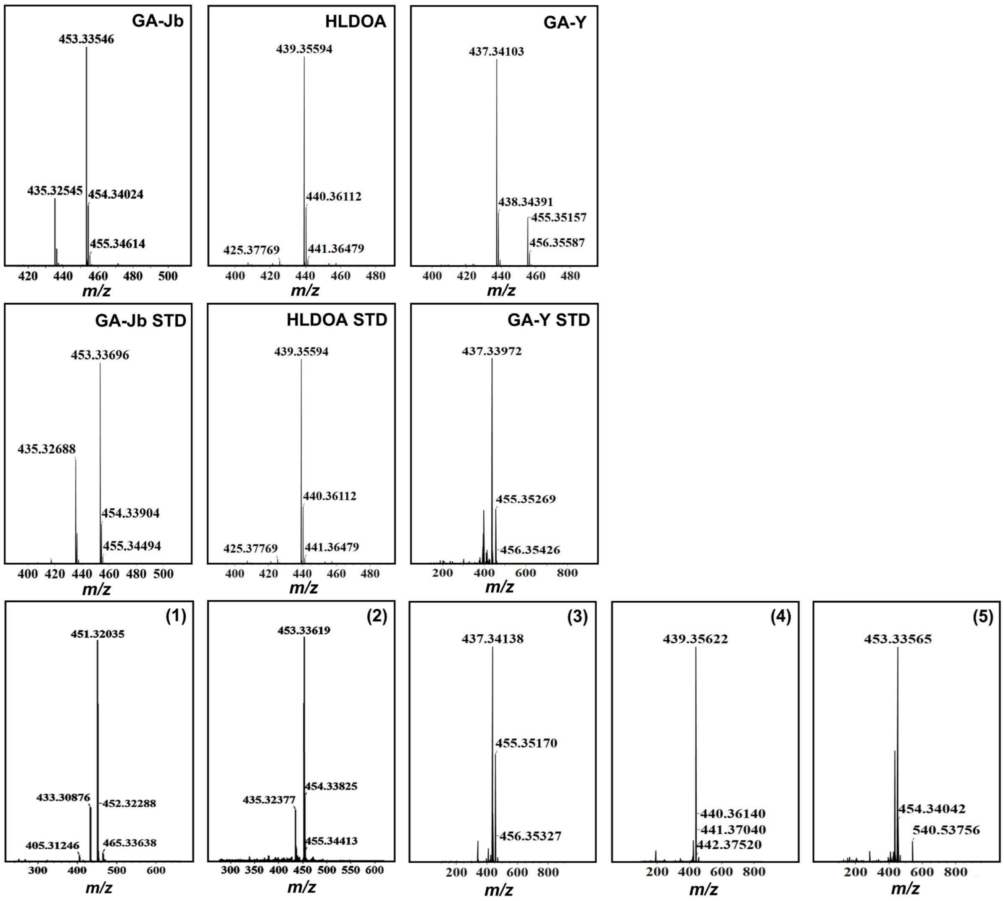


**Supplementary Fig. S9: Mass spectra of GA-Jb, HLDOA, GA-Y, their authentic standards (STD) and peaks 1-5.**

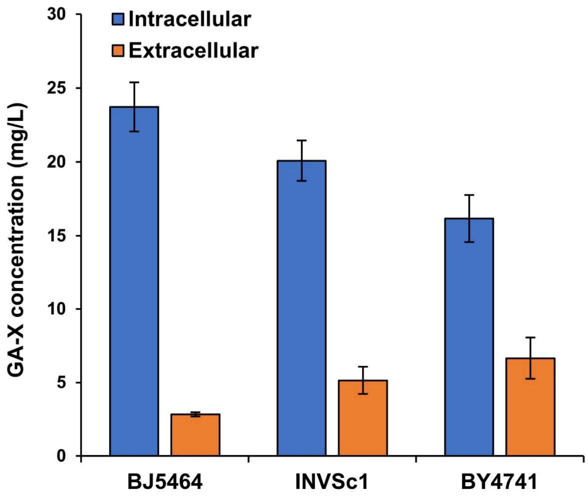


**Supplementary Fig. S10: Permeability of different yeasts to GA-X.** All data represent the average value calculated from three independent samples, and the error bars indicate the standard deviation.

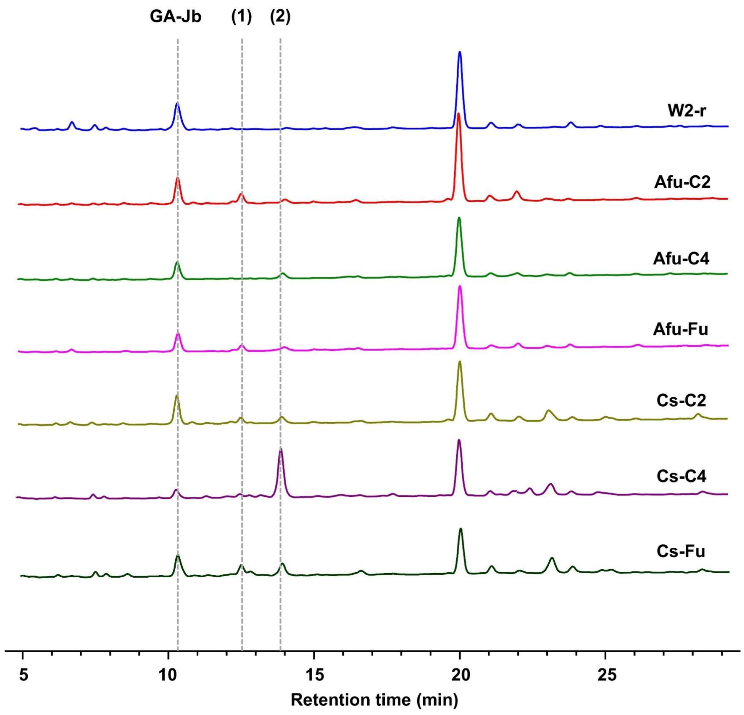


**Supplementary Fig. S11: HPLC analysis of fermentation extracts from yeasts expressing oxidase-reductase.** W2-r (SC62-CYP512W2-r), Afu-C2 (SC62-AfuSDR-AKR1C2-CYP512W2-r), Afu-C4 (SC62-AfuSDR-AKR1C4-CYP512W2-r), Afu-Fu (SC62-AfuSDR-FusC1-CYP512W2-r), Cs-C2 (SC62-CsSDR-AKR1C2-CYP512W2-r), Cs-C4 (SC62-CsSDR-AKR1C4-CYP512W2-r), and Cs-Fu (SC62-CsSDR-FusC1-CYP512W2-r).

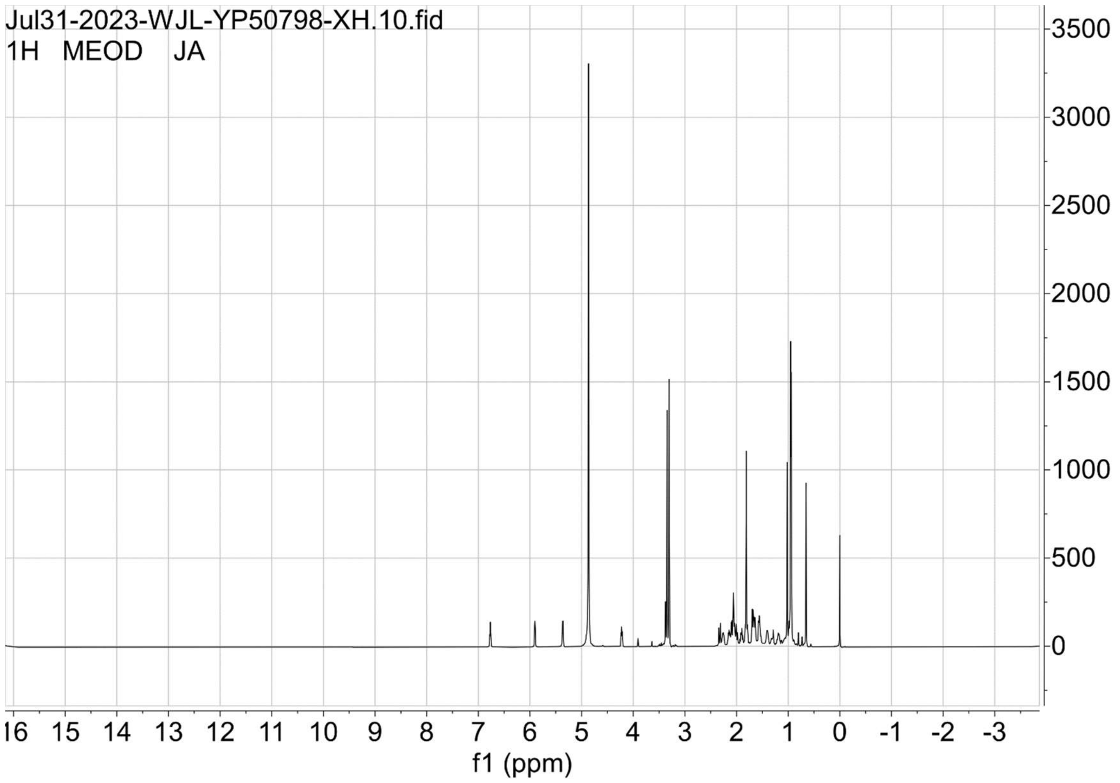


**Supplementary Fig. S12: $^{1}$H NMR spectrum of 2.**

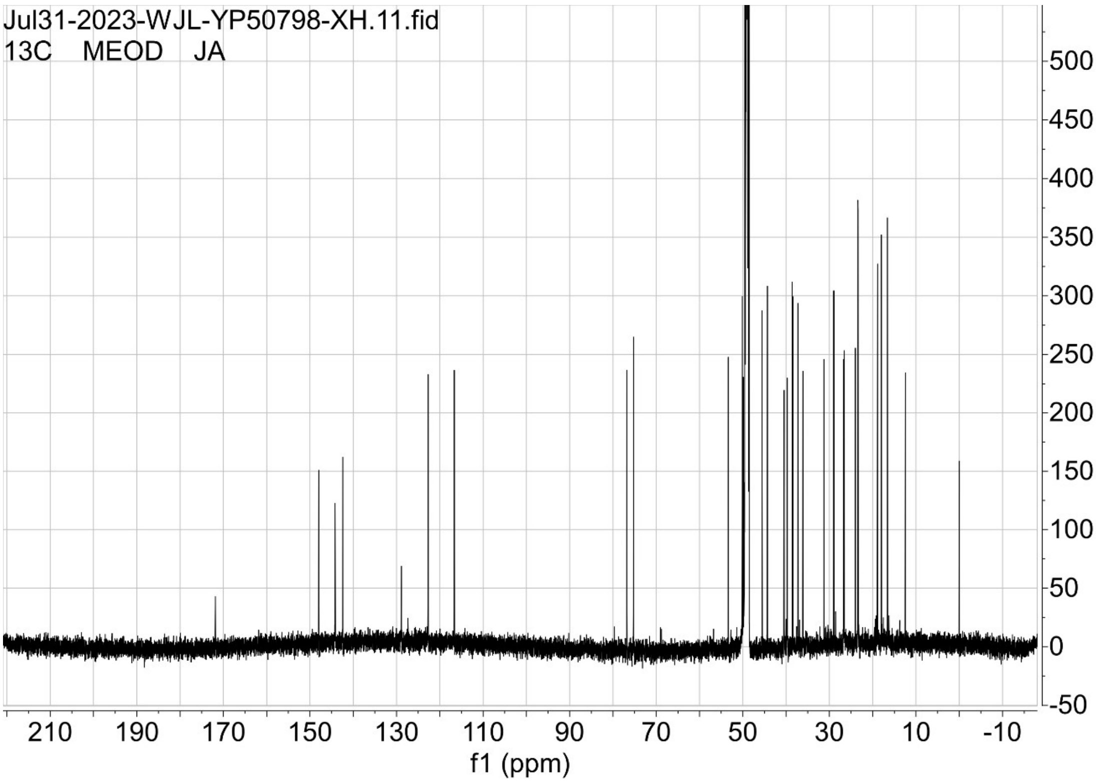


**Supplementary Fig. S13: $^{13}$C NMR spectrum of 2.**

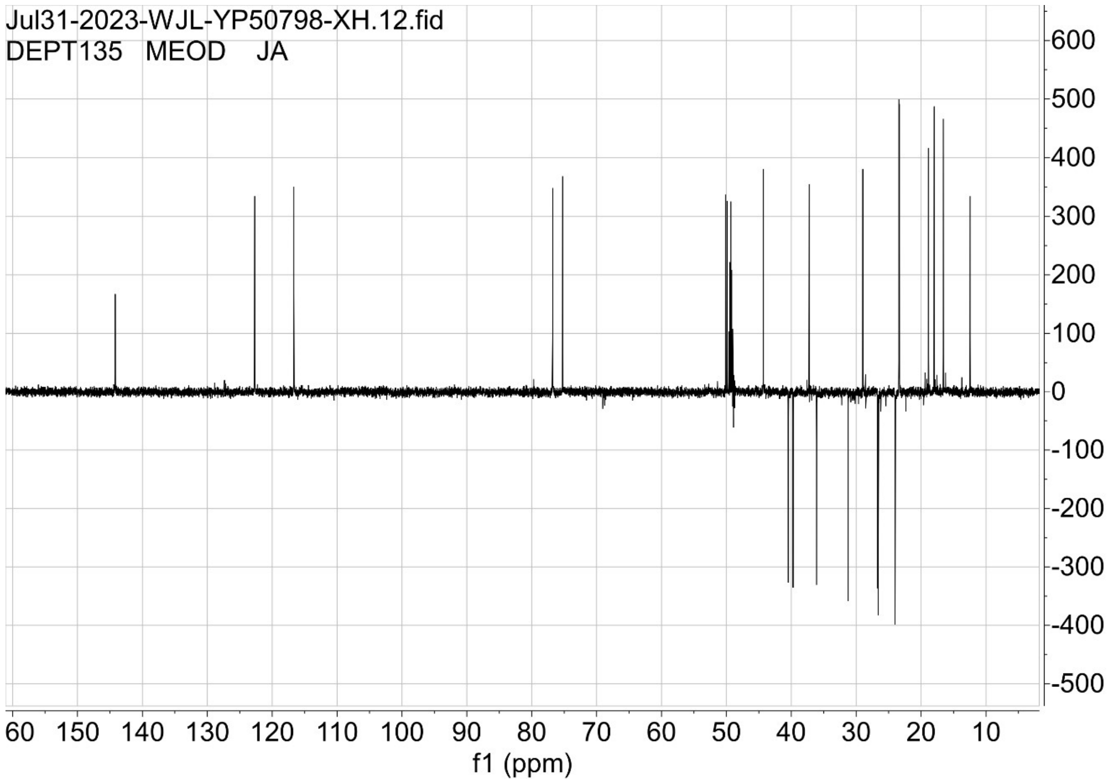


**Supplementary Fig. S14: DEPT-135 spectrum of 2.**

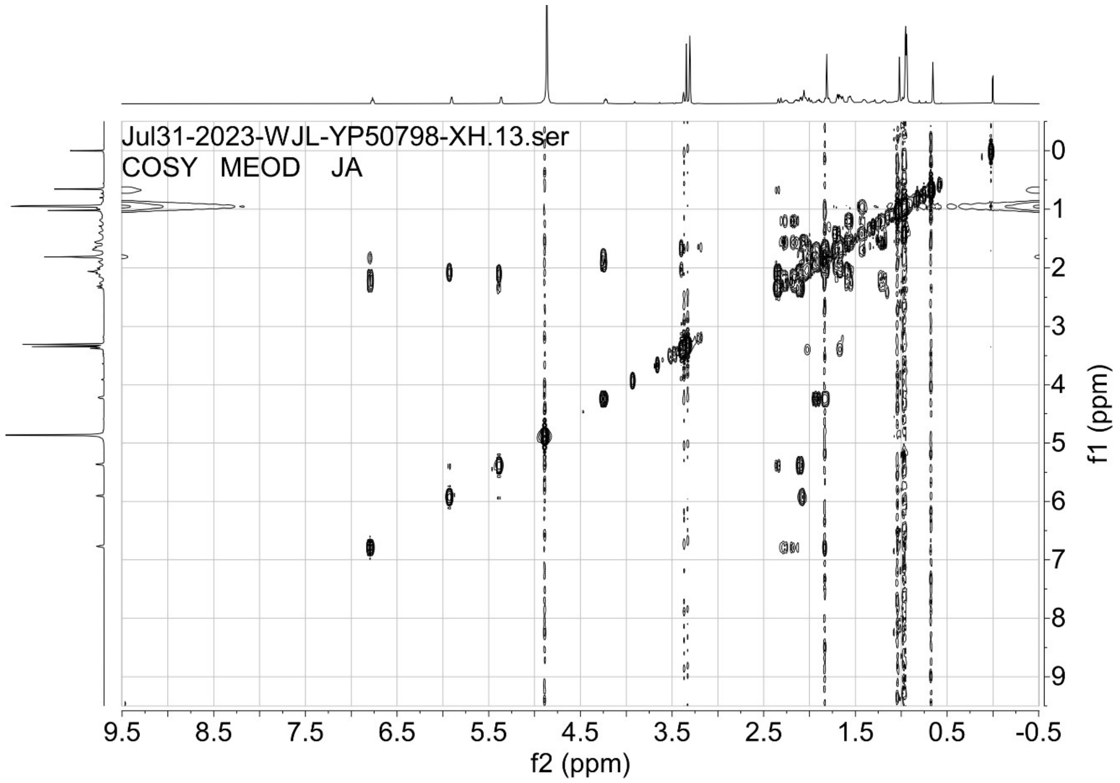


**Supplementary Fig. S15: COSY spectrum of 2.**

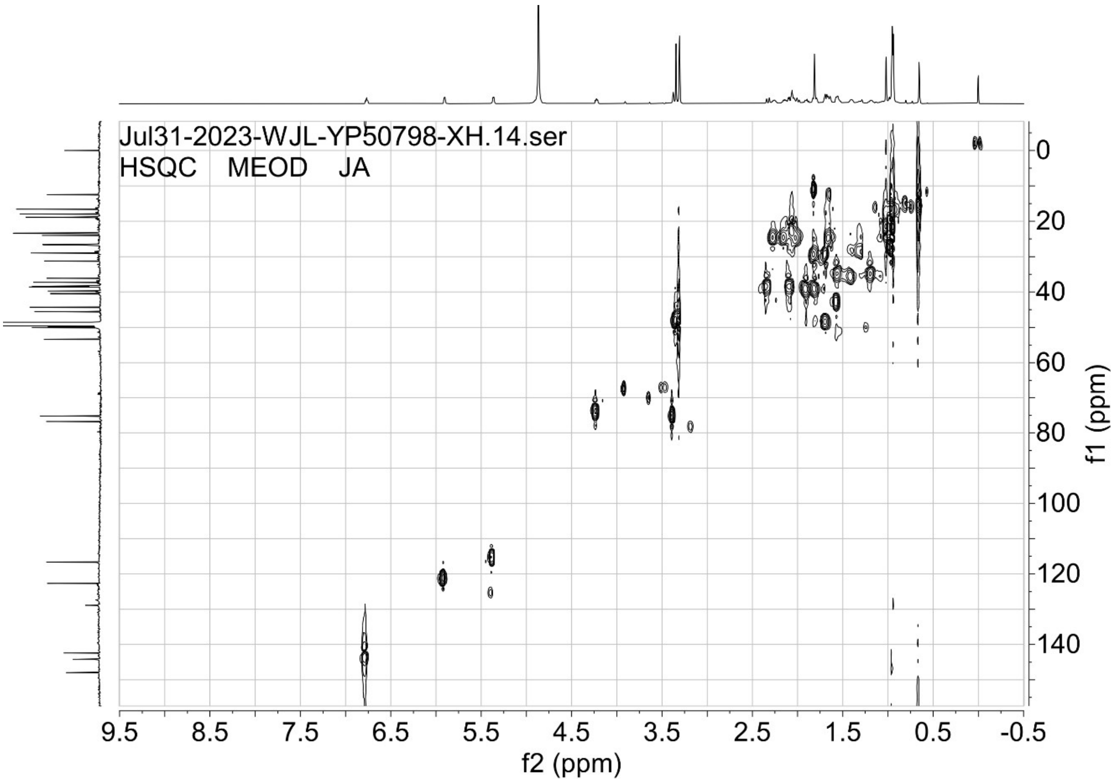


**Supplementary Fig. S16: HSQC spectrum of 2.**

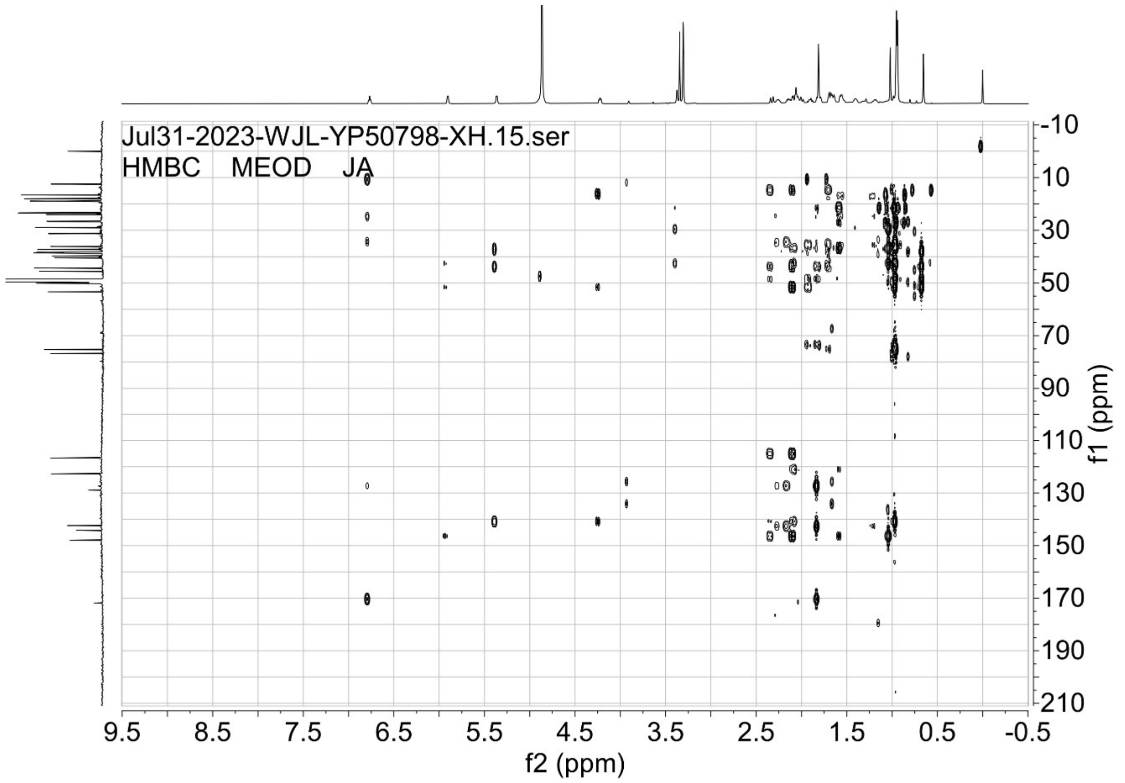


**Supplementary Fig. S17: HMBC spectrum of 2.**

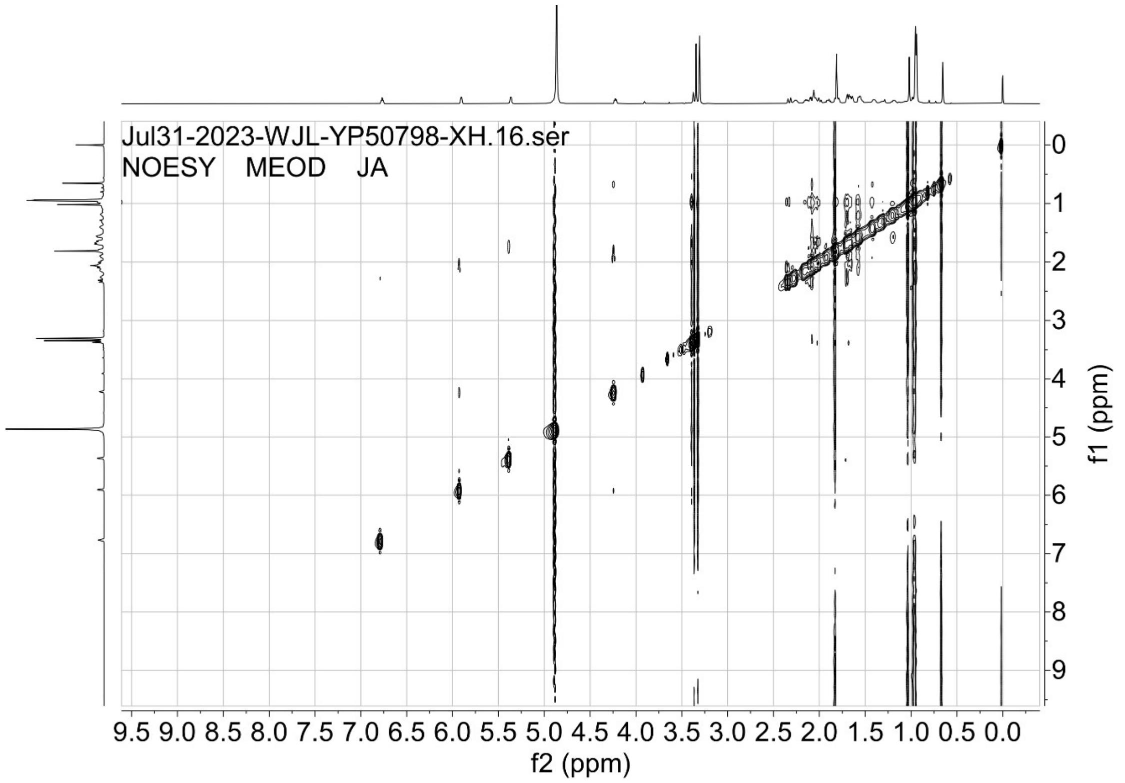


**Supplementary Fig. S18: NOESY spectrum of 2.**

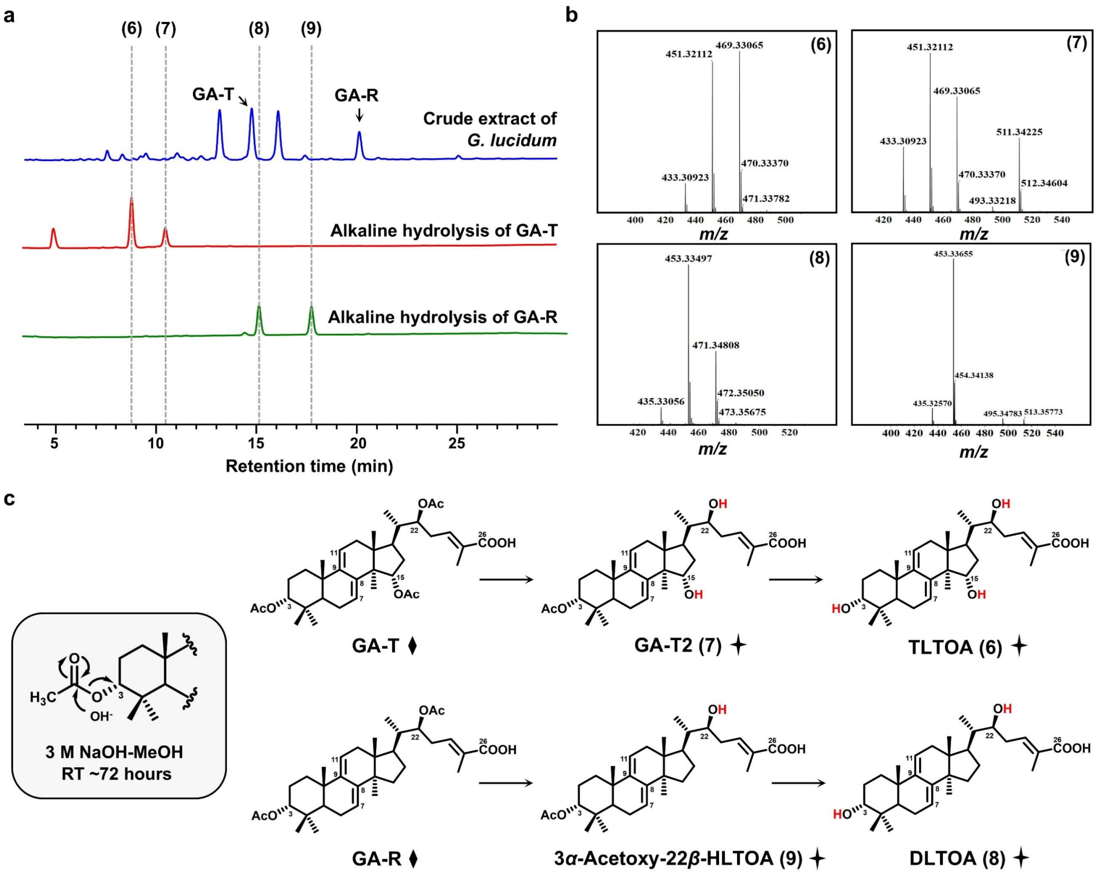


**Supplementary Fig. S19: Alkaline hydrolysis of GA-T and GA-R for the preparation of C22-hydroxylated TIIGAs. a.** HPLC analysis of crude extracts of *G. lucidum*, alkaline hydrolysis extracts of GA-T and GA-R. **b.** Mass spectra of peaks **6-9**. **c.** Proposed deacetylation process by alkaline hydrolysis of GA-T and GA-R. The compound marked with a diamond indicates that its chemical structure is confirmed by comparison with an authentic standard. The compound marked with a star indicates that its chemical structure is confirmed by NMR analyses.

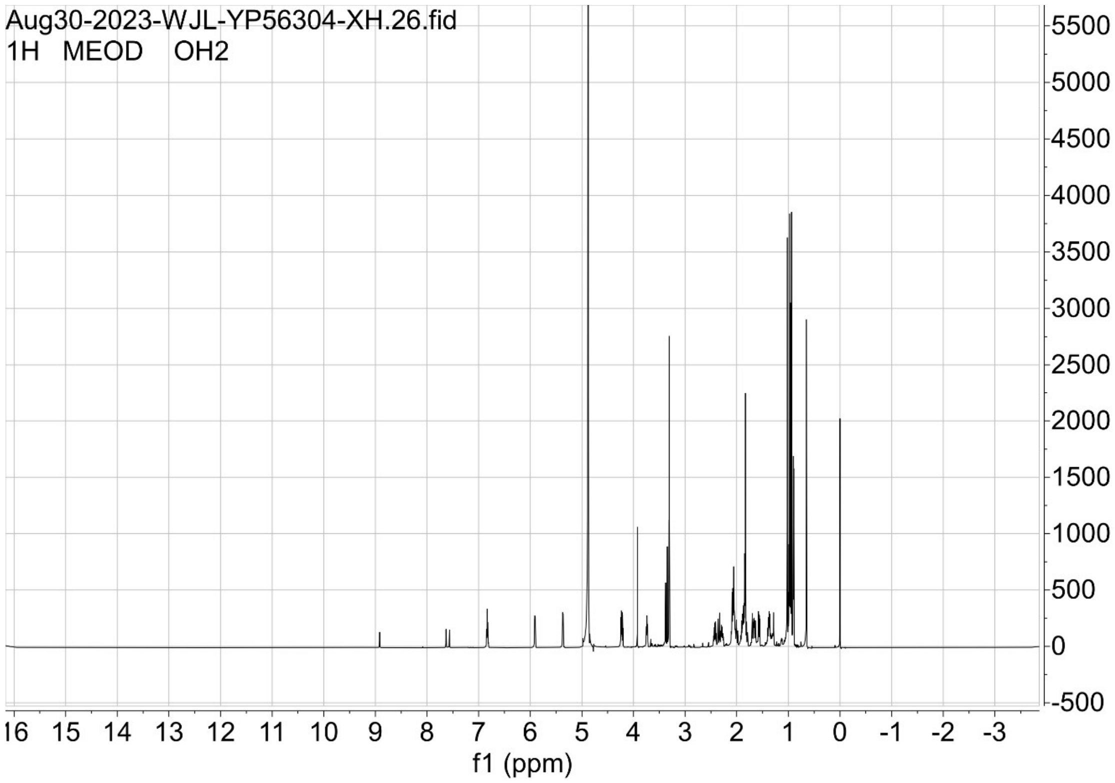


**Supplementary Fig. S20: $^{1}$H NMR spectrum of 6.**

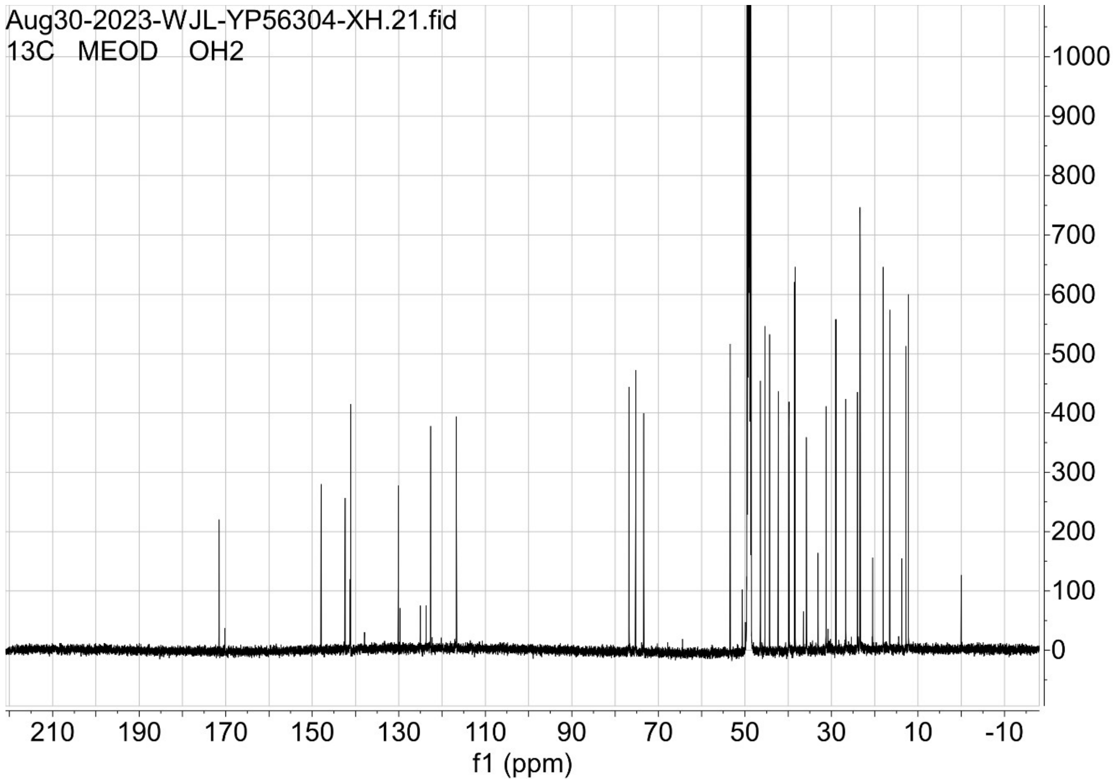


**Supplementary Fig. S21: $^{13}C$ NMR spectrum of 6.**

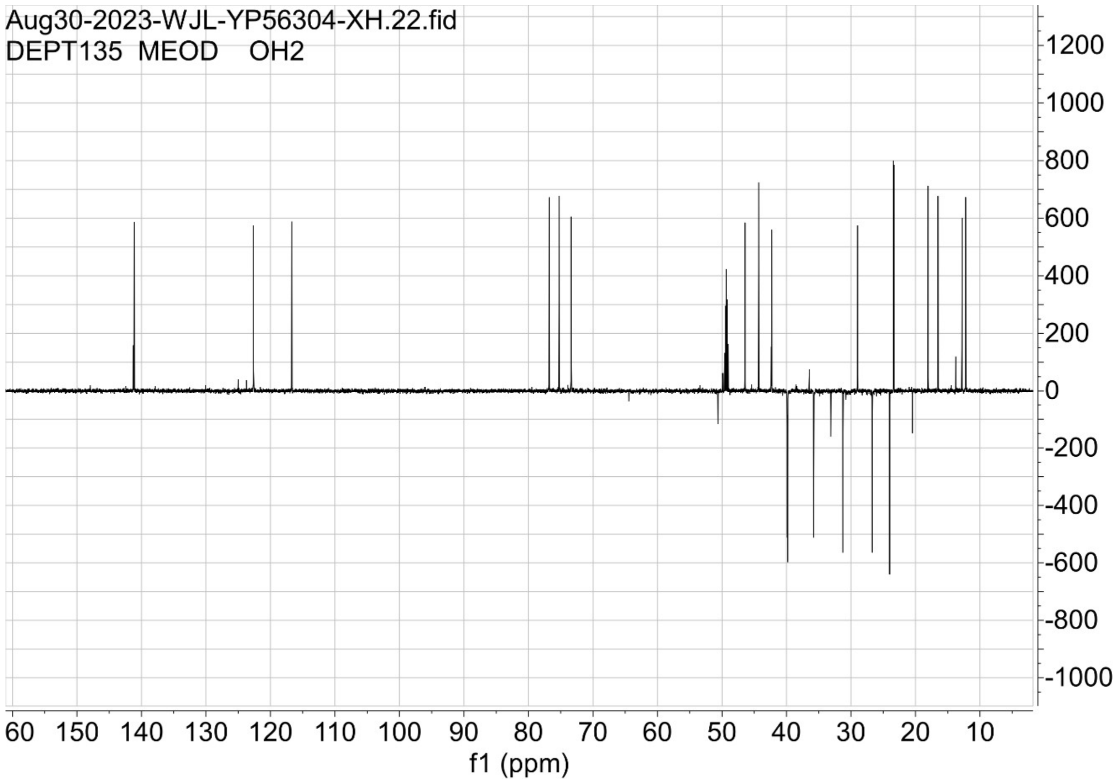


**Supplementary Fig. S22: DEPT-135 spectrum of 6.**

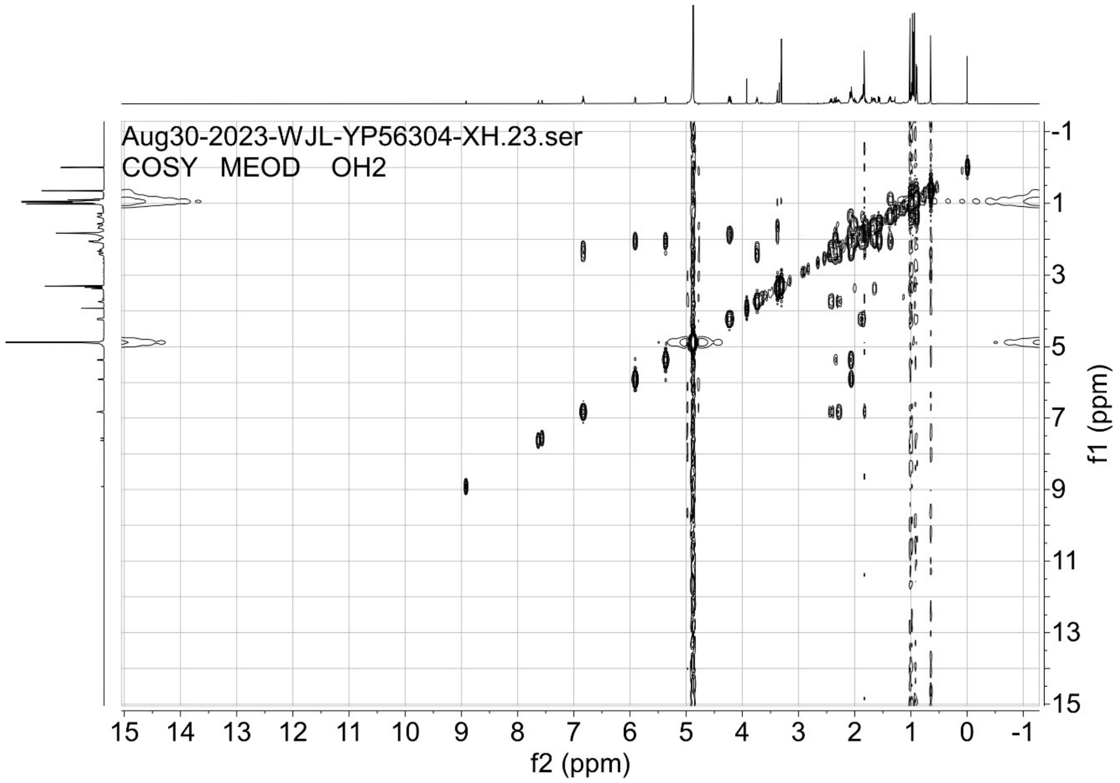


**Supplementary Fig. S23: COSY spectrum of 6.**

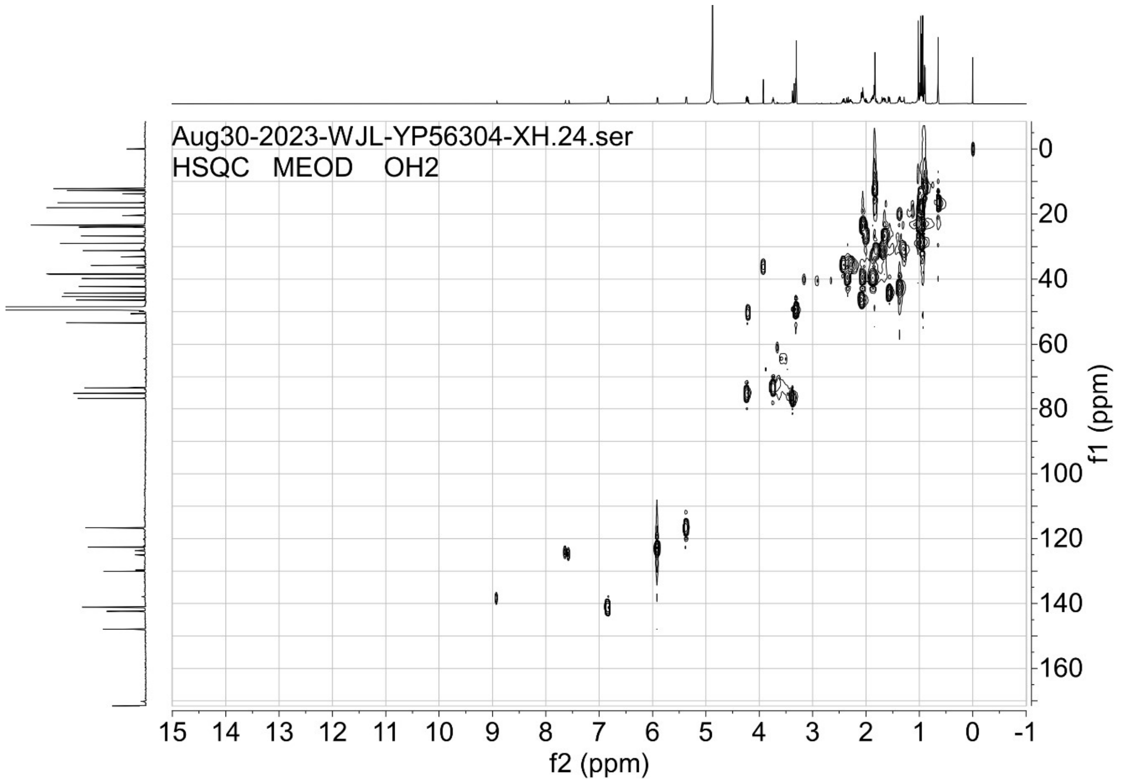


**Supplementary Fig. S24: HSQC spectrum of 6.**

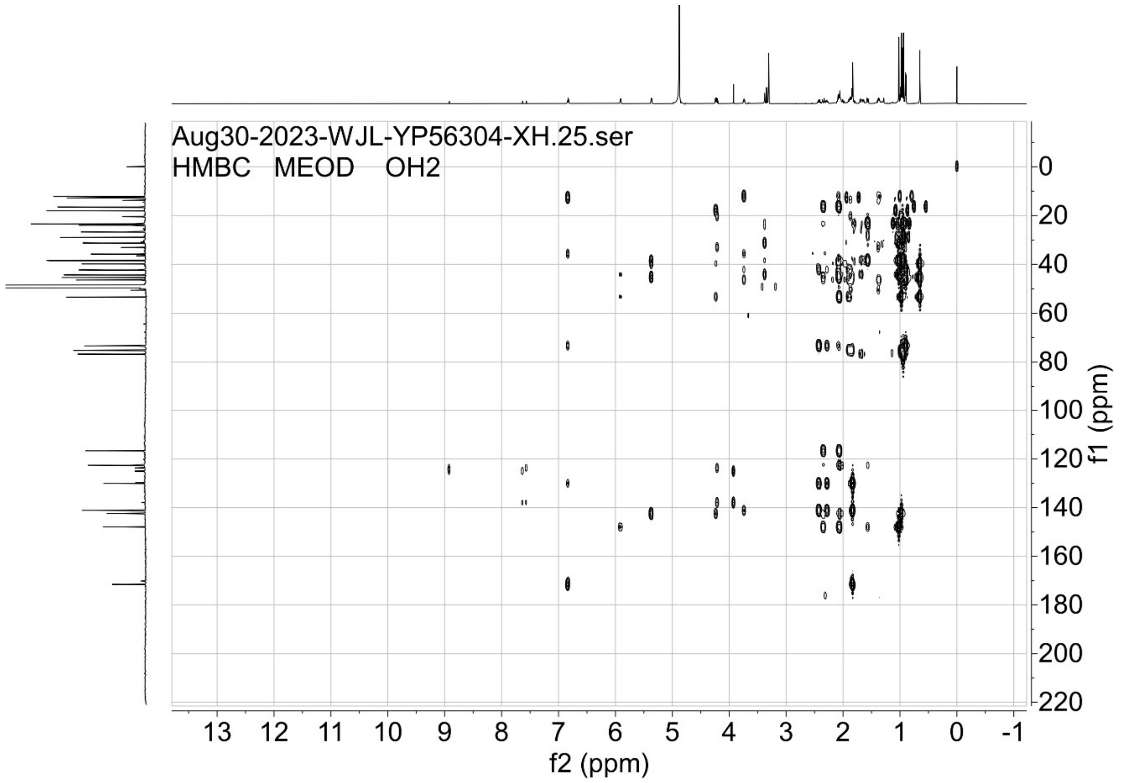


**Supplementary Fig. S25: HMBC spectrum of 6.**

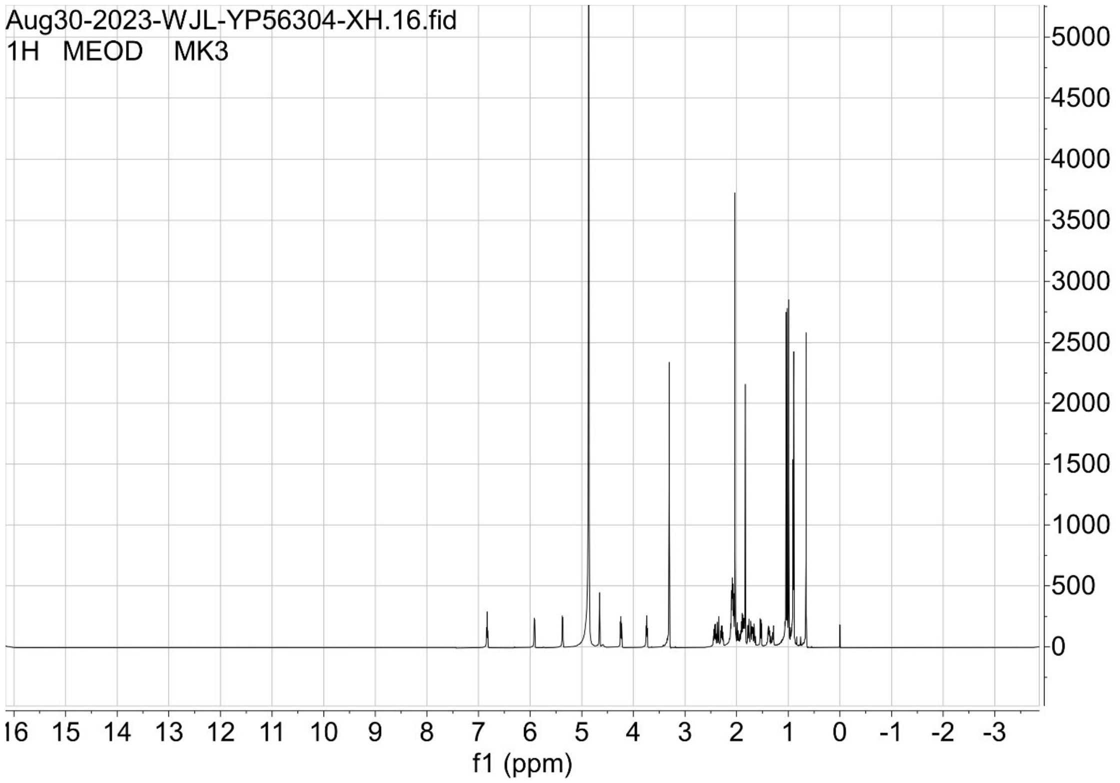


**Supplementary Fig. S26: <sup></sup>¹H NMR spectrum of 7.**

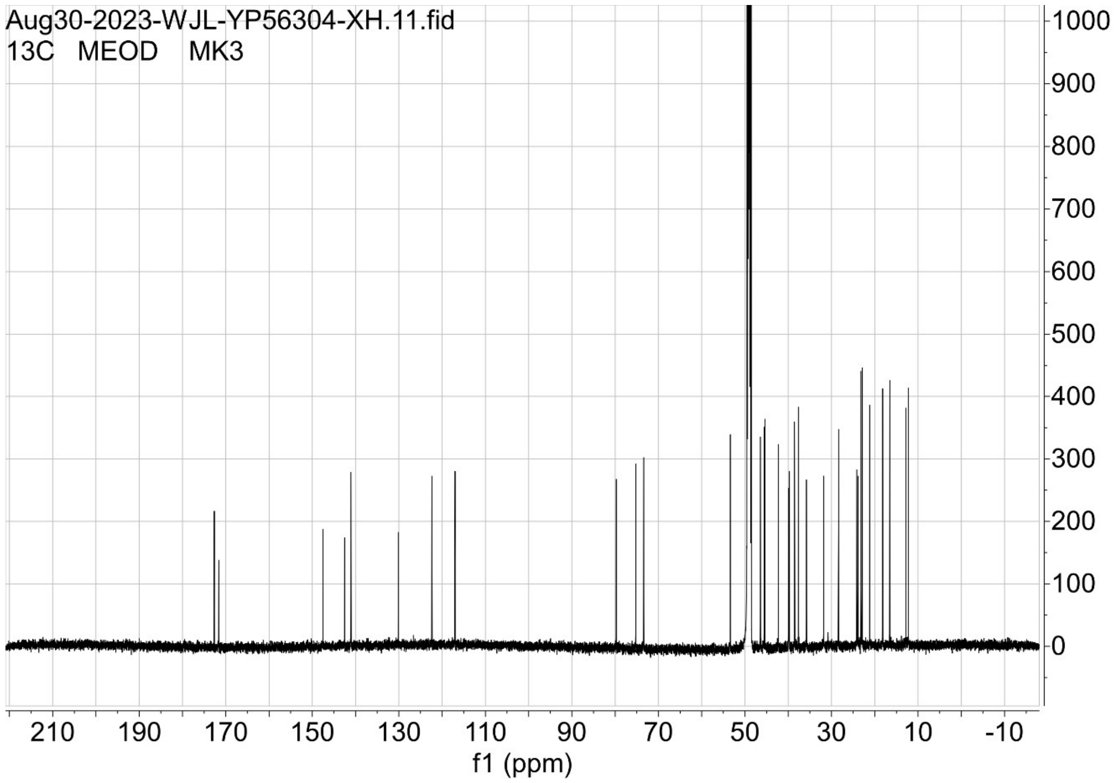


**Supplementary Fig. S27: $^{13}$C NMR spectrum of 7.**

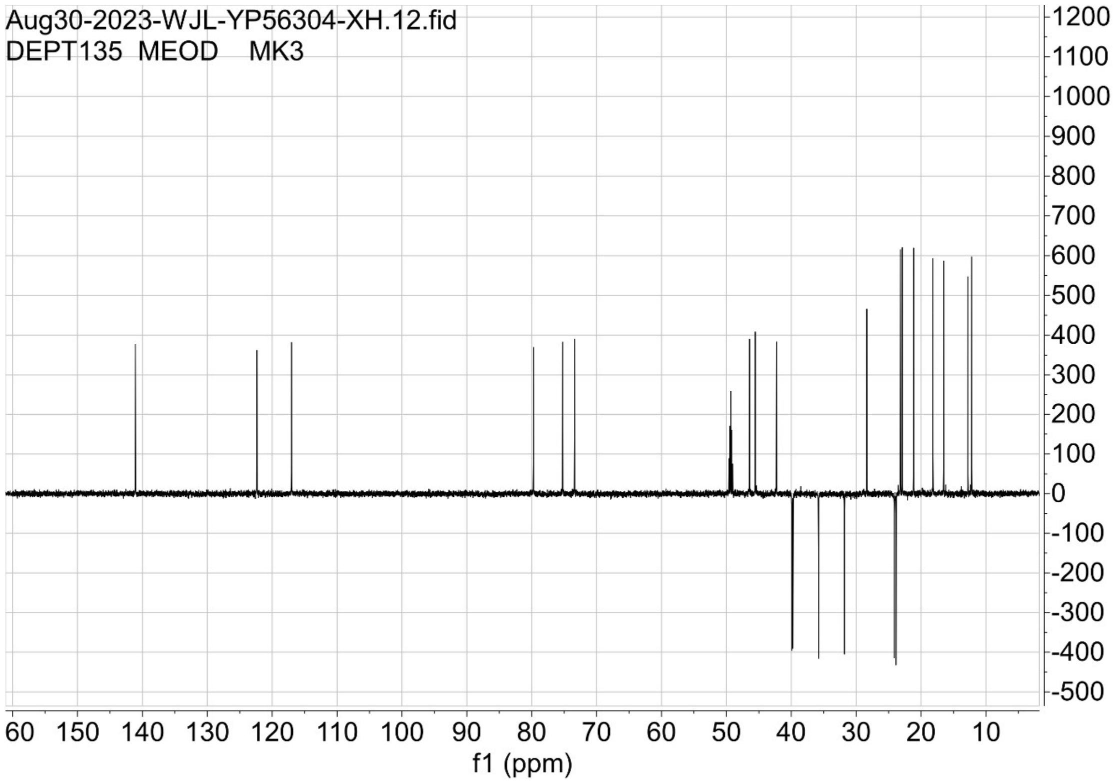


**Supplementary Fig. S28: DEPT-135 spectrum of 7.**

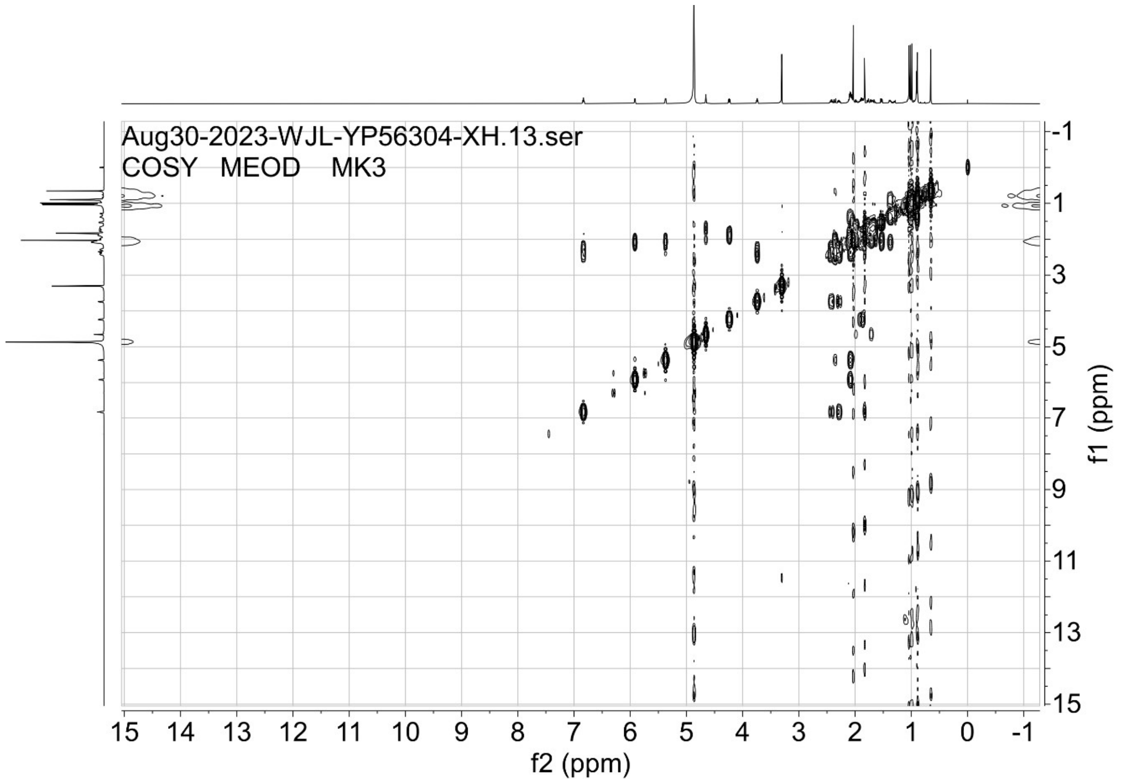


**Supplementary Fig. S29: COSY spectrum of 7.**

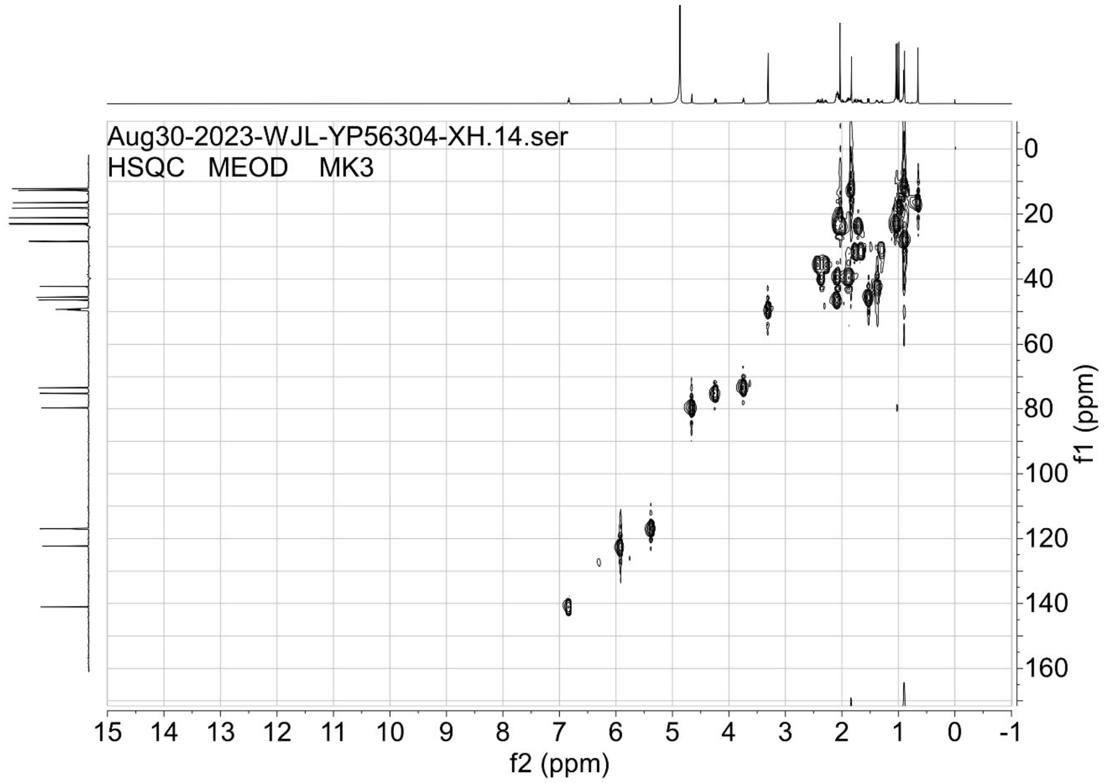


**Supplementary Fig. S30: HSQC spectrum of 7.**

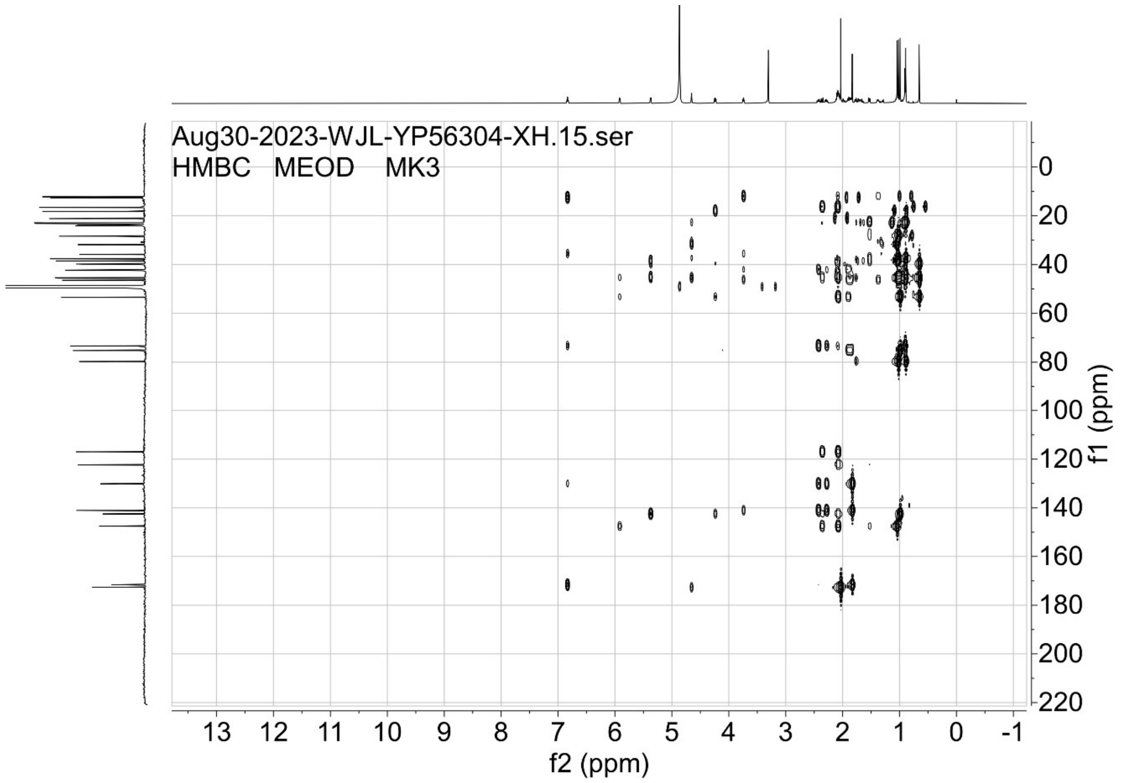


**Supplementary Fig. S31: HMBC spectrum of 7.**

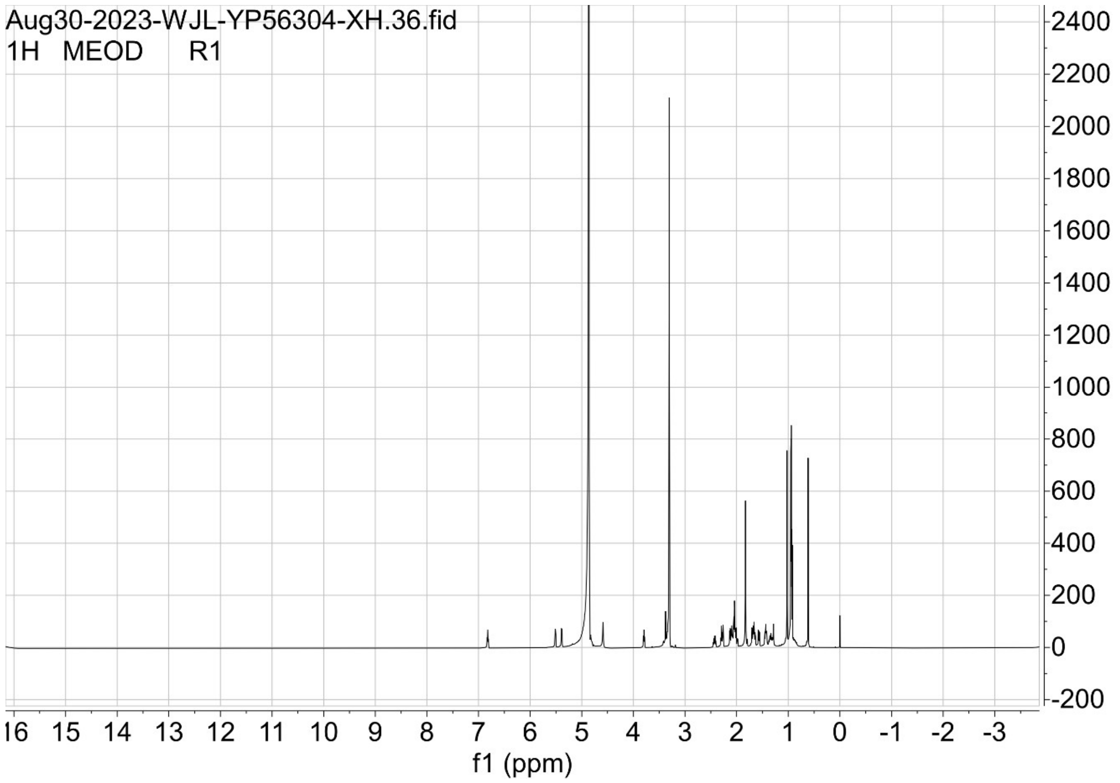


**Supplementary Fig. S32: $^{1}$H NMR spectrum of 8.**

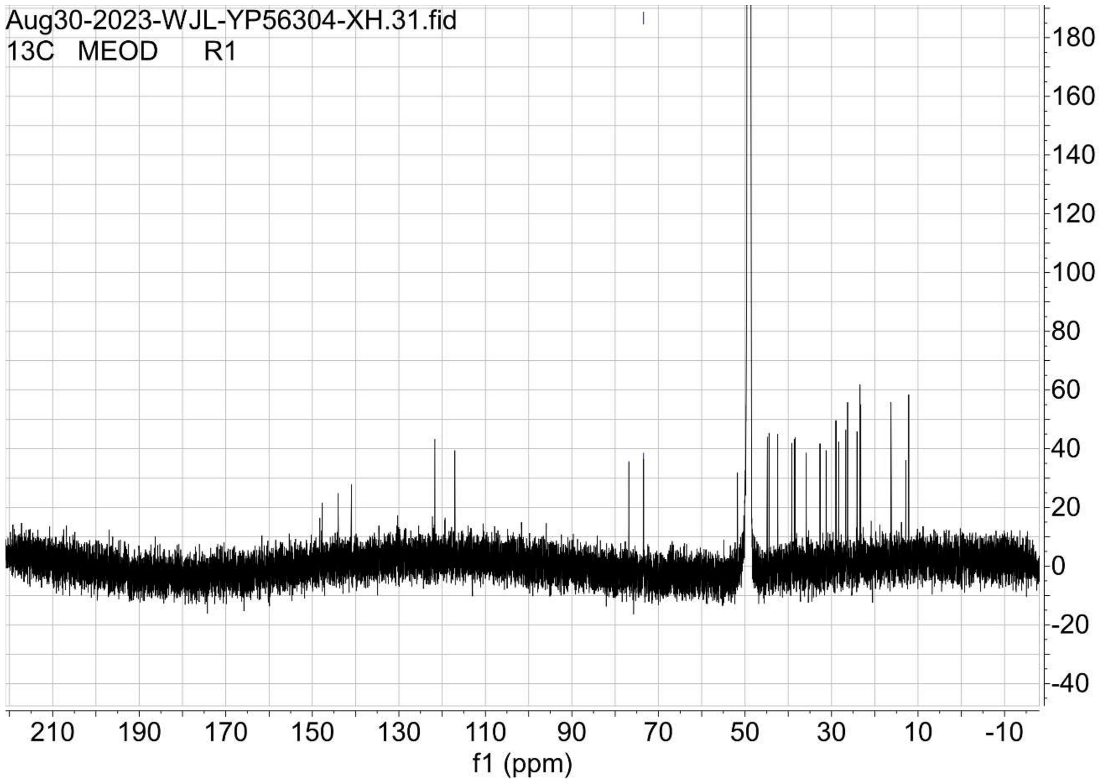


**Supplementary Fig. S33: $^{13}$C NMR spectrum of 8.**

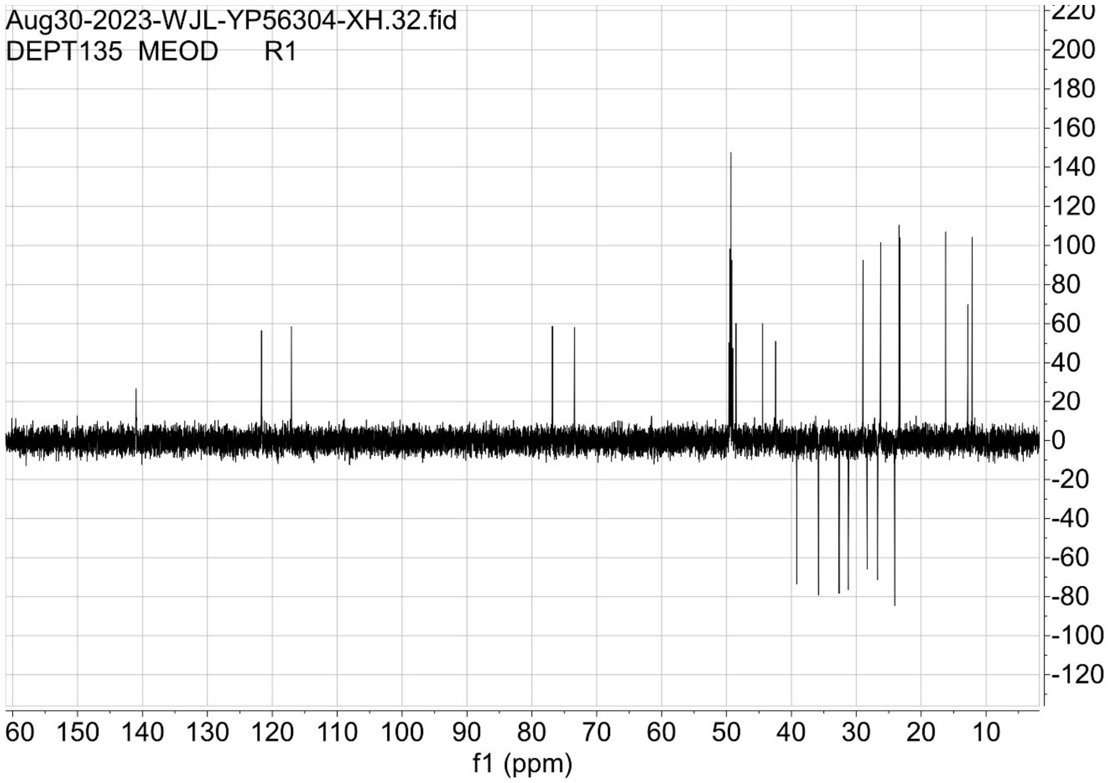


**Supplementary Fig. S34: DEPT-135 spectrum of 8.**

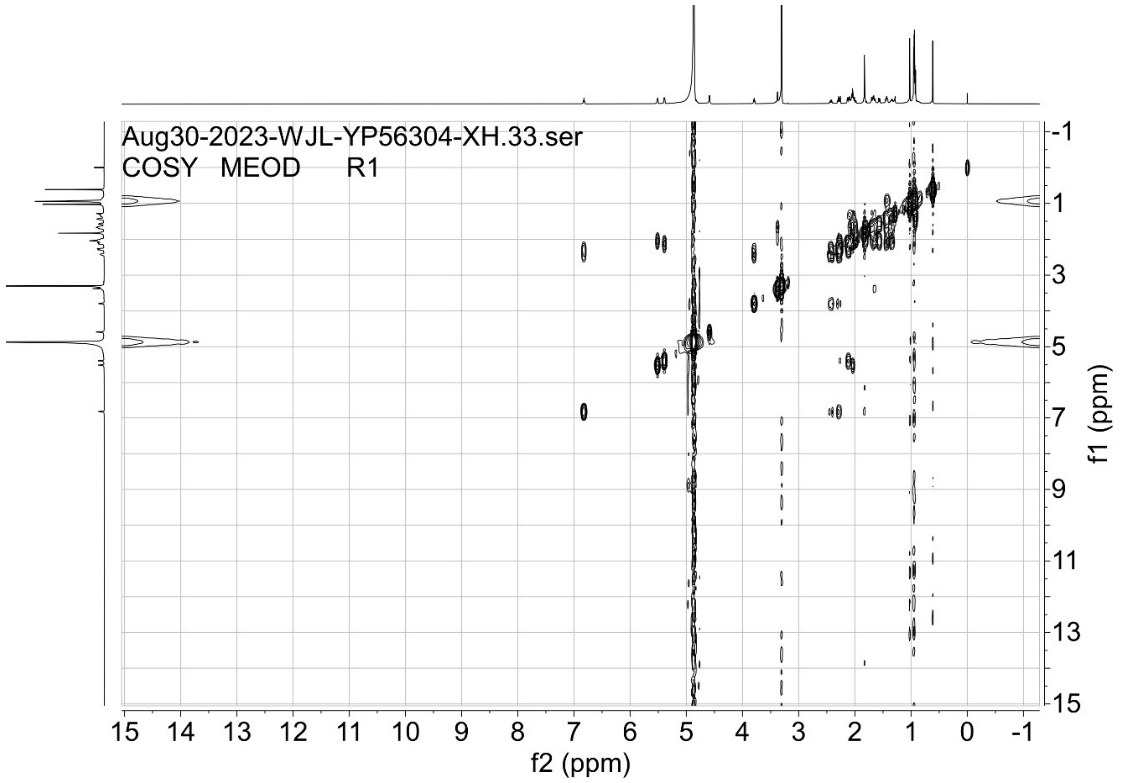


**Supplementary Fig. S35: COSY spectrum of 8.**

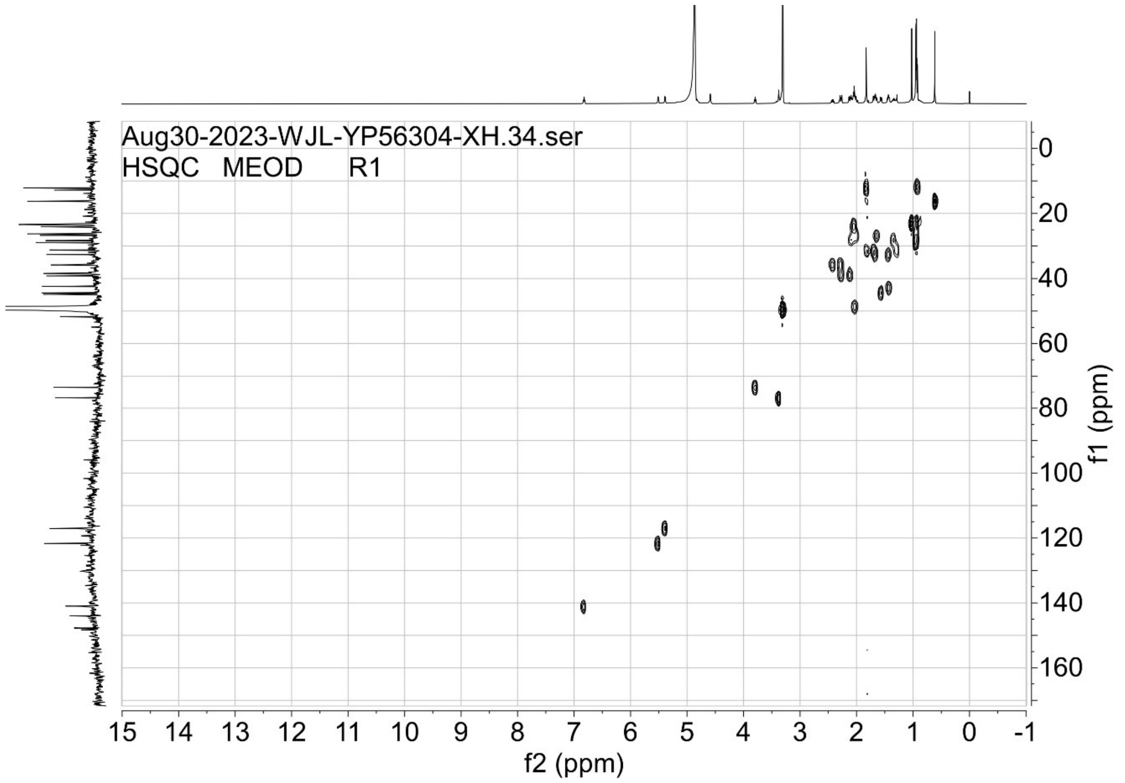


**Supplementary Fig. S36: HSQC spectrum of 8.**

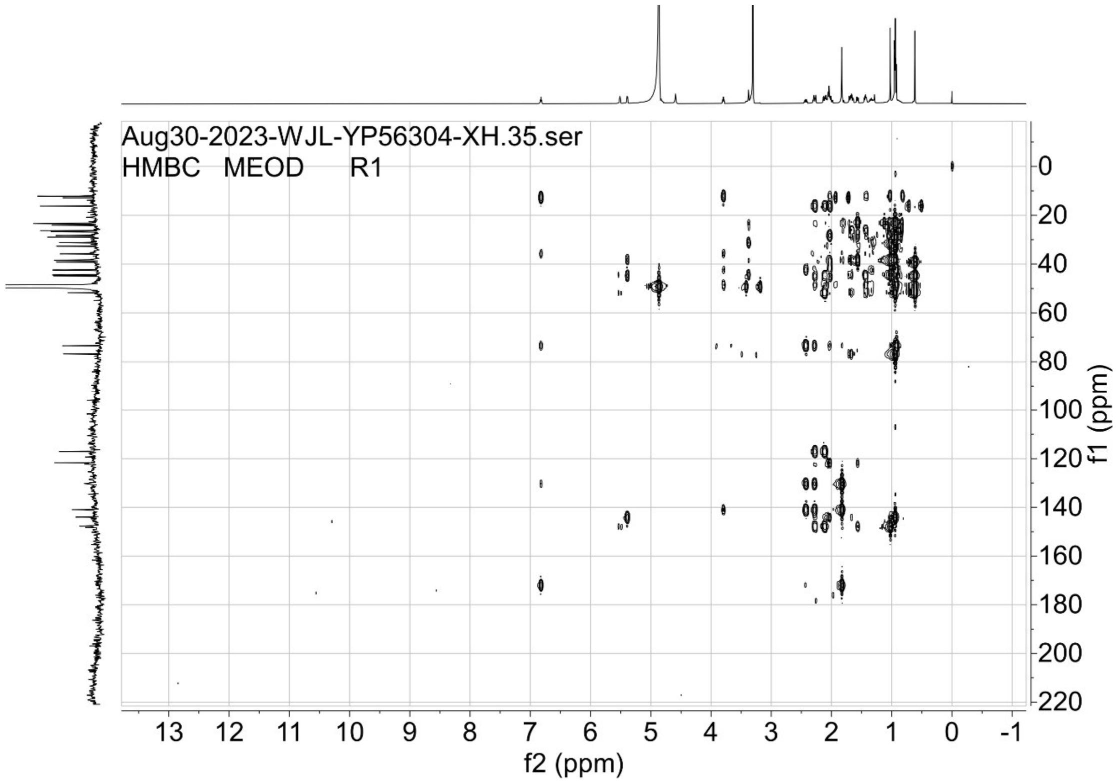


**Supplementary Fig. S37: HMBC spectrum of 8.**

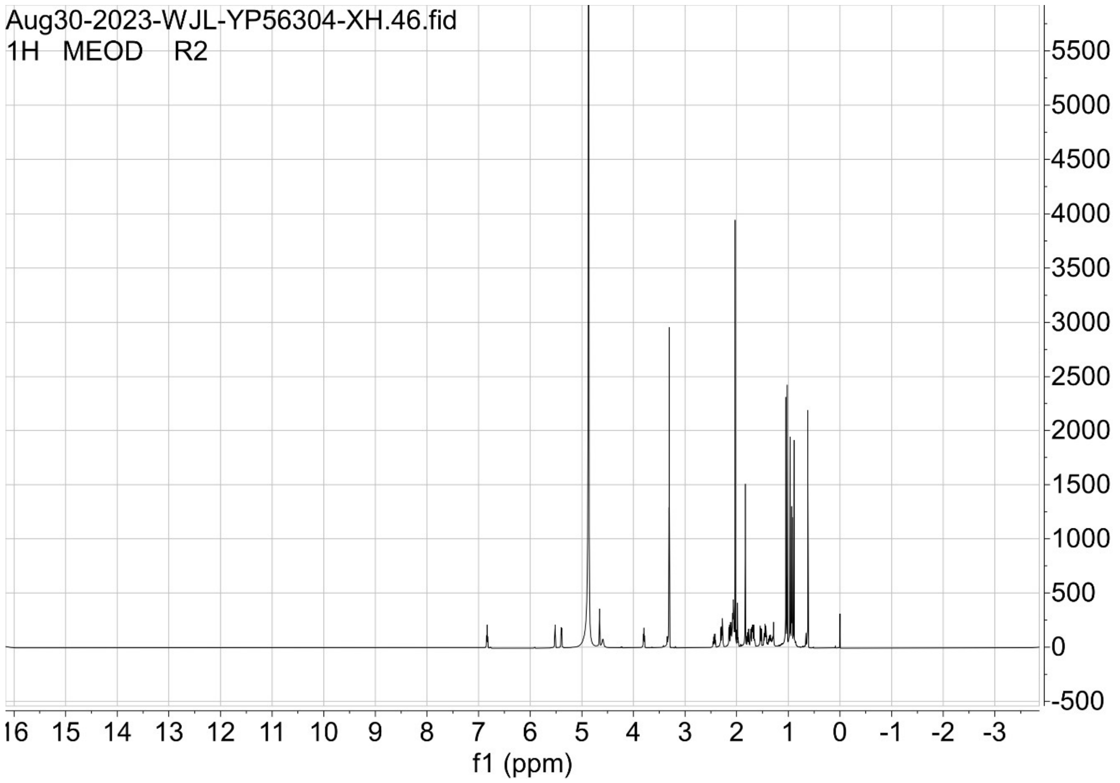


**Supplementary Fig. S38: $^{1}$H NMR spectrum of 9.**

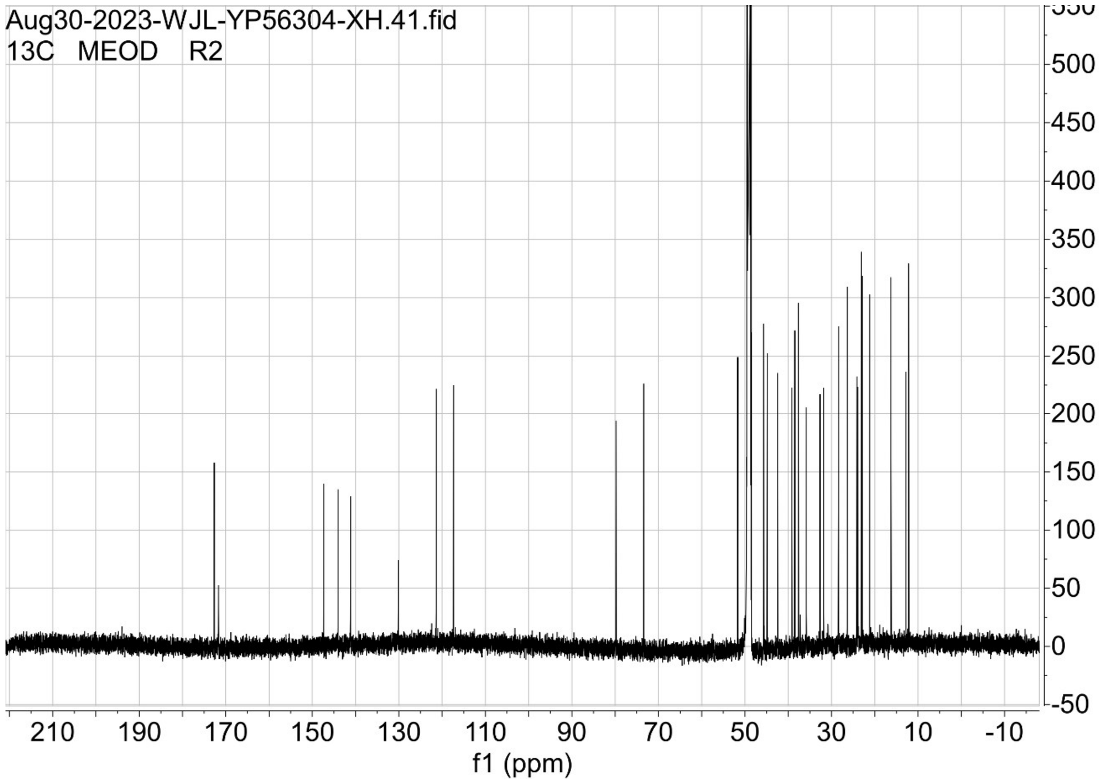


**Supplementary Fig. S39: $^{13}C$ NMR spectrum of 9.**

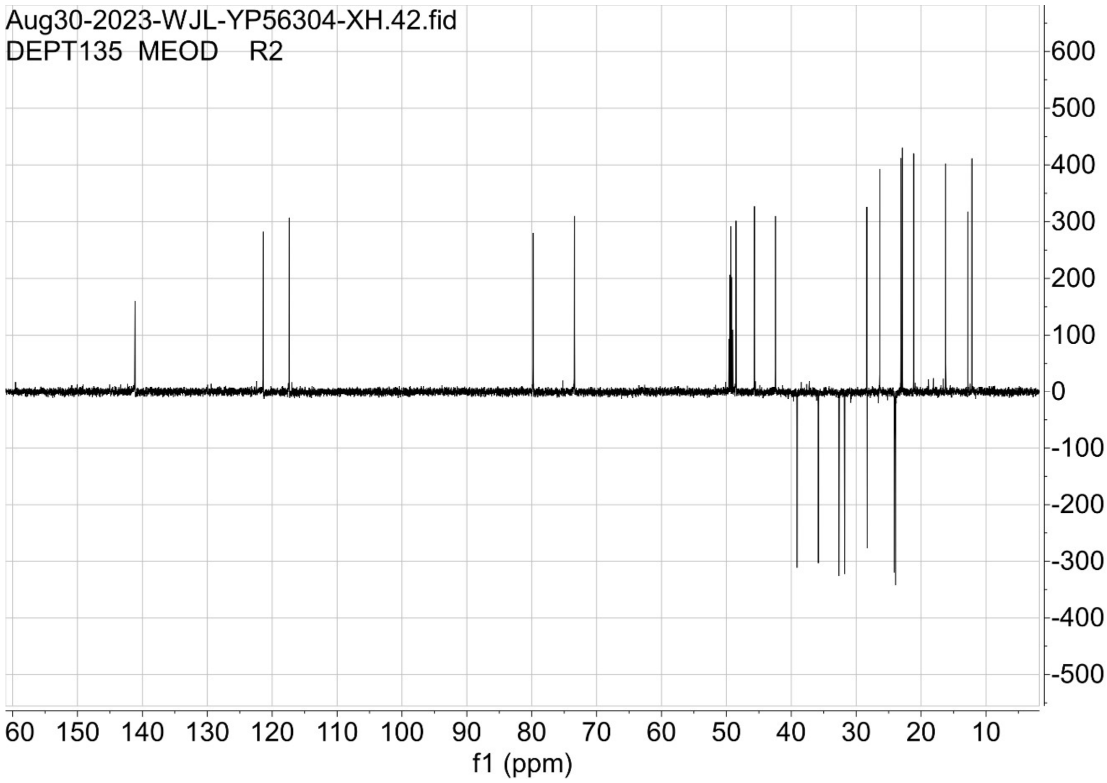


**Supplementary Fig. S40: DEPT-135 spectrum of 9.**

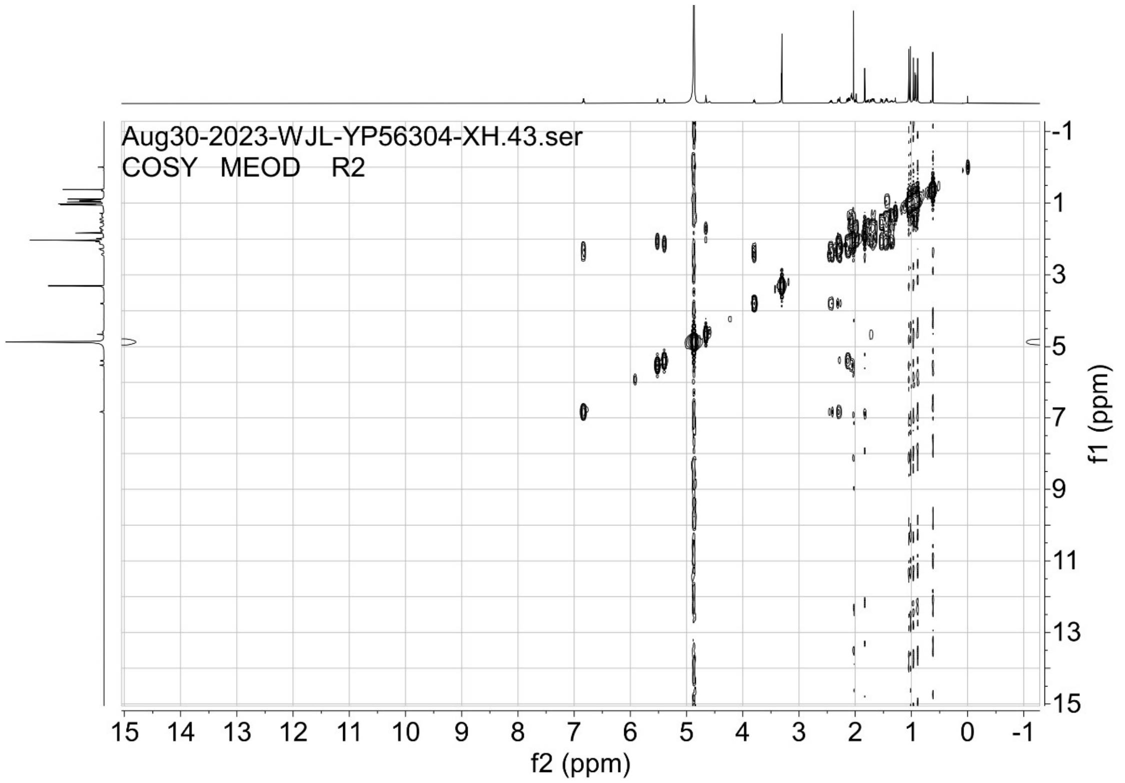


**Supplementary Fig. S41: COSY spectrum of 9.**

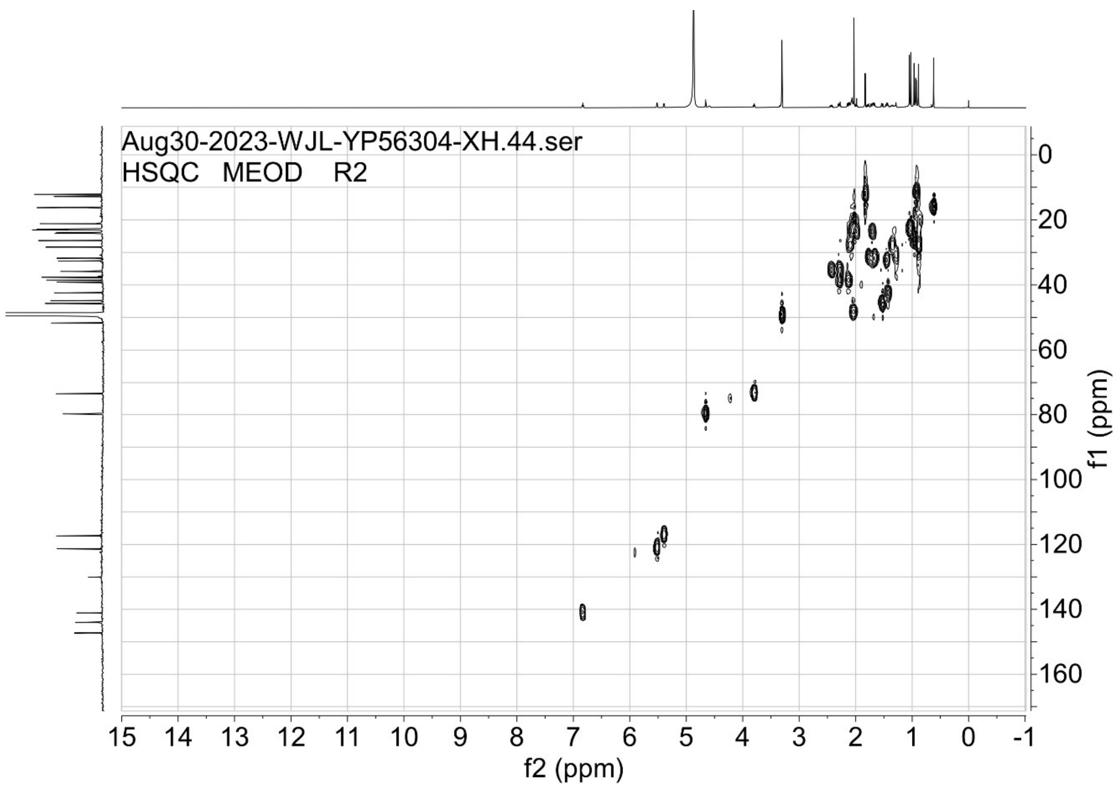


**Supplementary Fig. S42: HSQC spectrum of 9.**

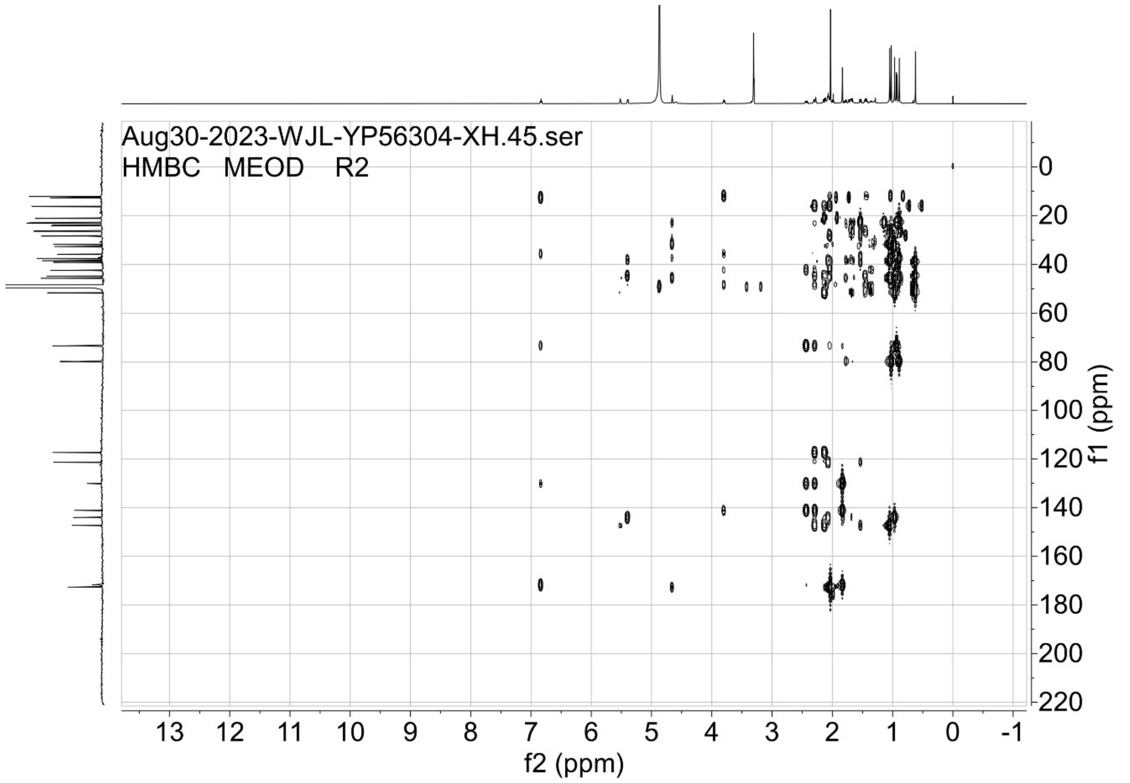


**Supplementary Fig. S43: HMBC spectrum of 9.**

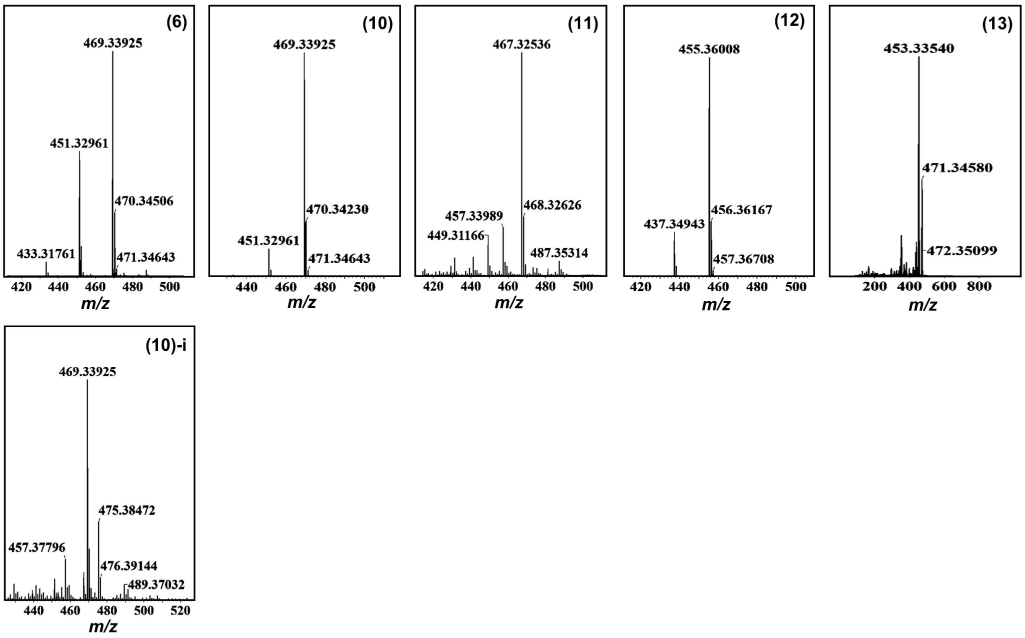


**Supplementary Fig. S44: Mass spectra of peaks 6, 10, 11, 12, 13 (from fermentation extracts) and 10-i (from *in vitro* reaction extracts).**

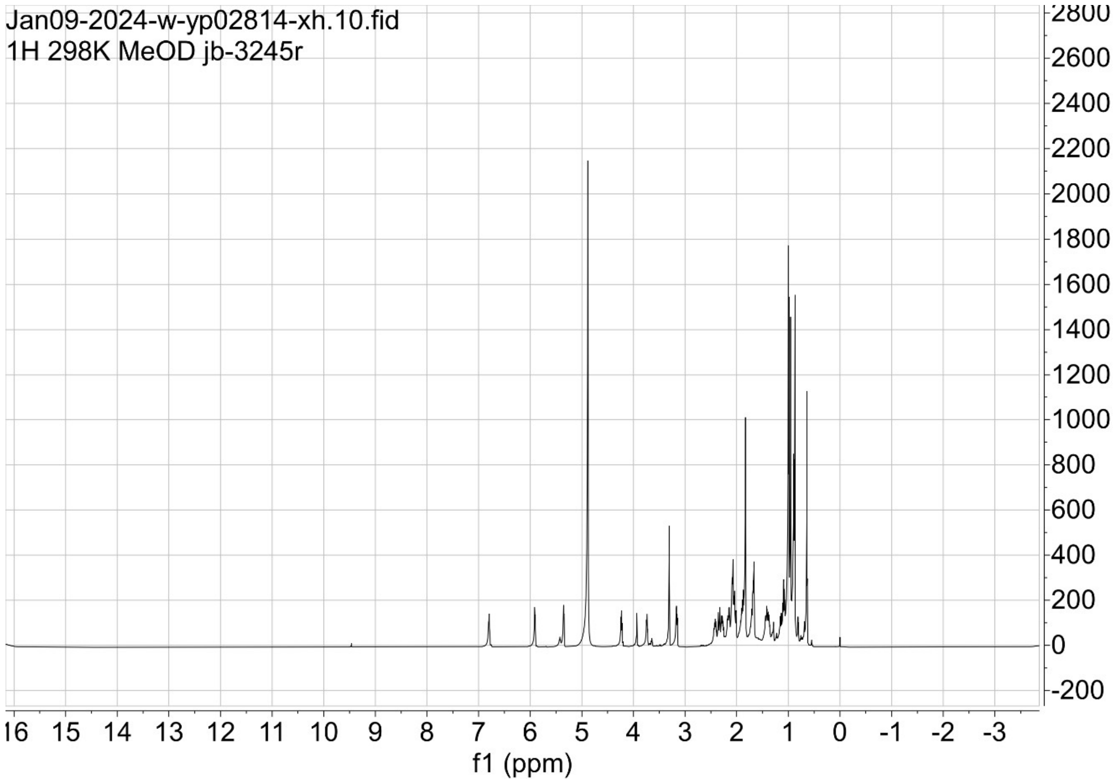


**Supplementary Fig. S45: $^{1}$H NMR spectrum of 10.**

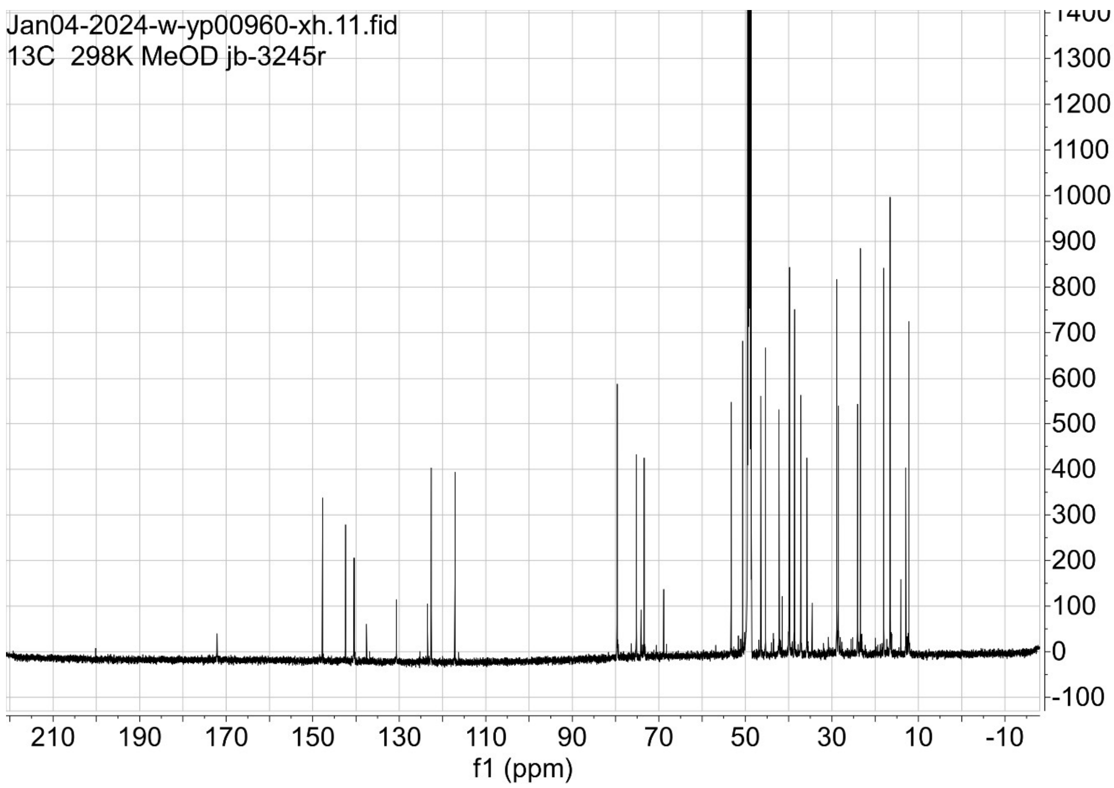


**Supplementary Fig. S46: $^{13}$C NMR spectrum of 10.**

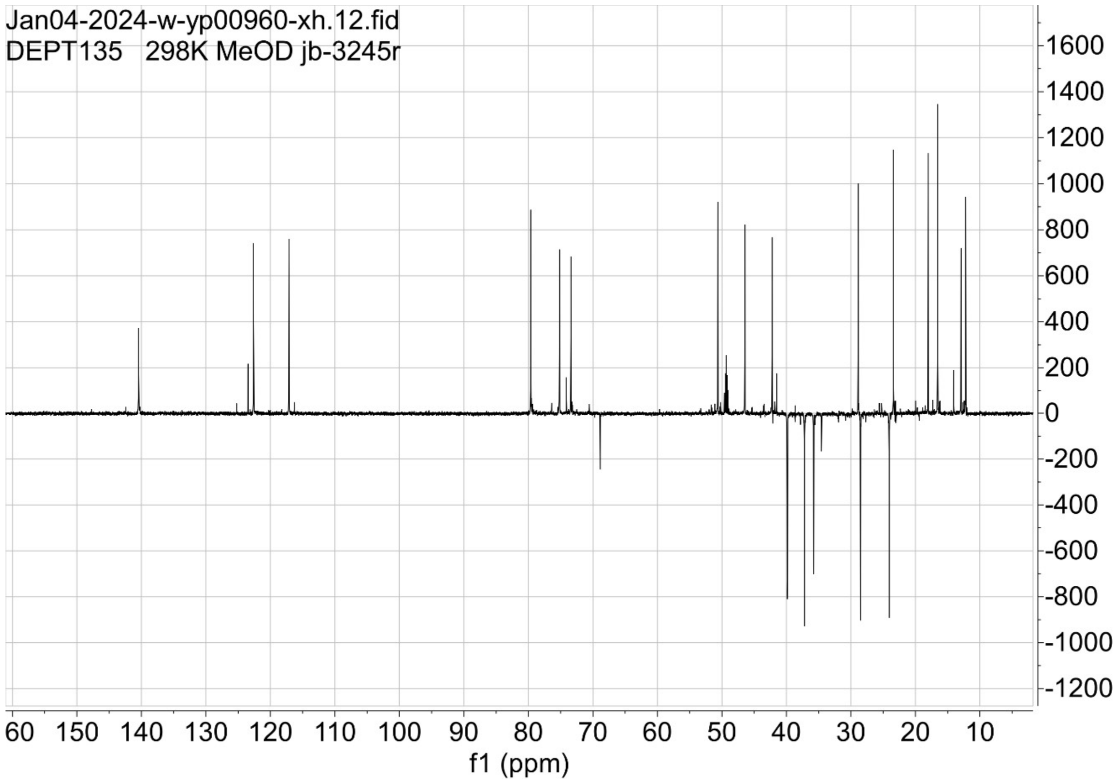


**Supplementary Fig. S47: DEPT-135 spectrum of 10.**

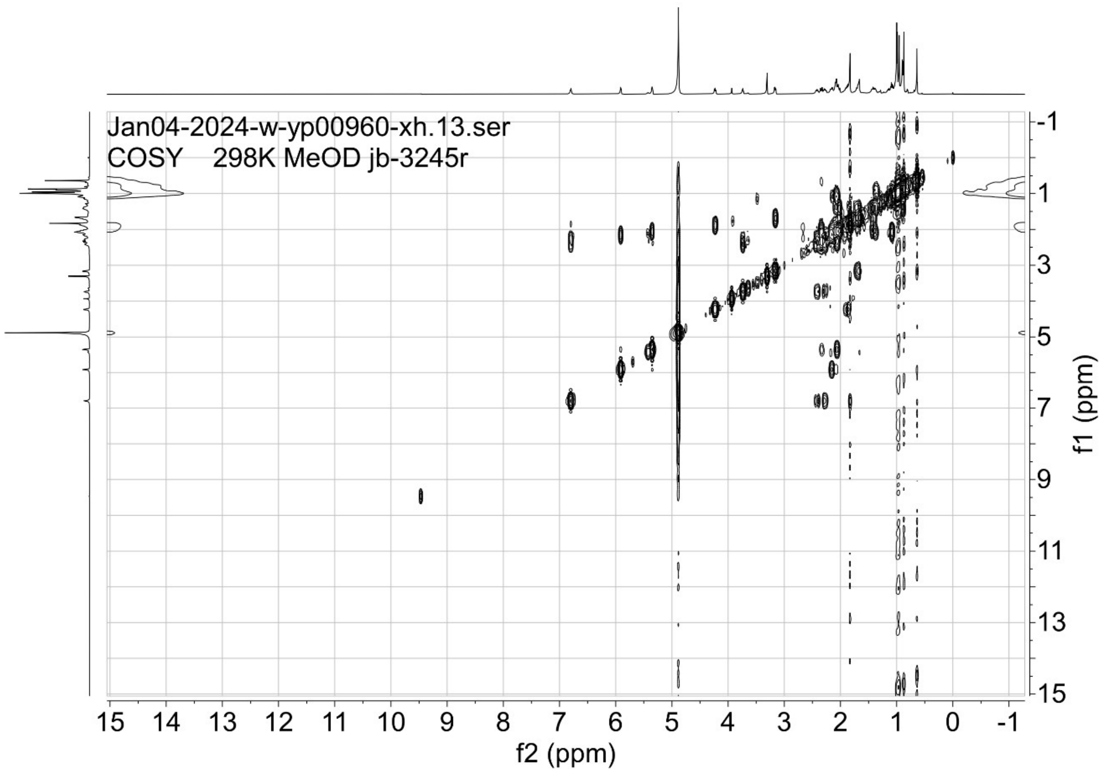


**Supplementary Fig. S48: COSY spectrum of 10.**

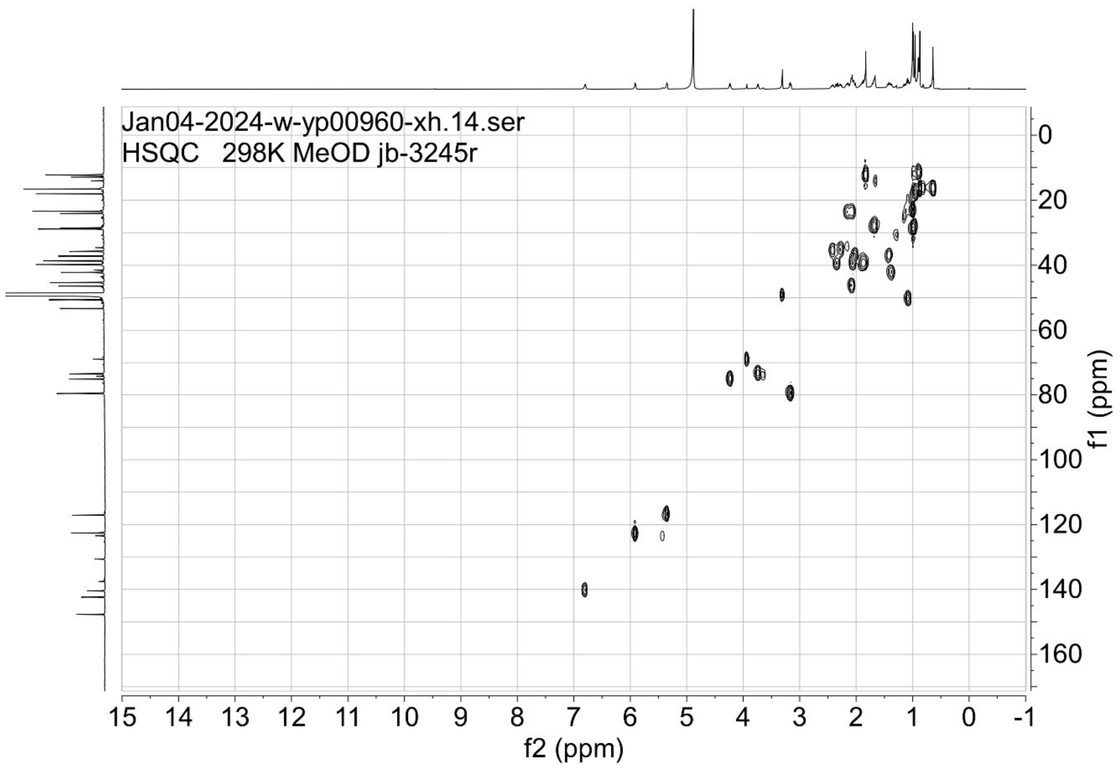


**Supplementary Fig. S49: HSQC spectrum of 10.**

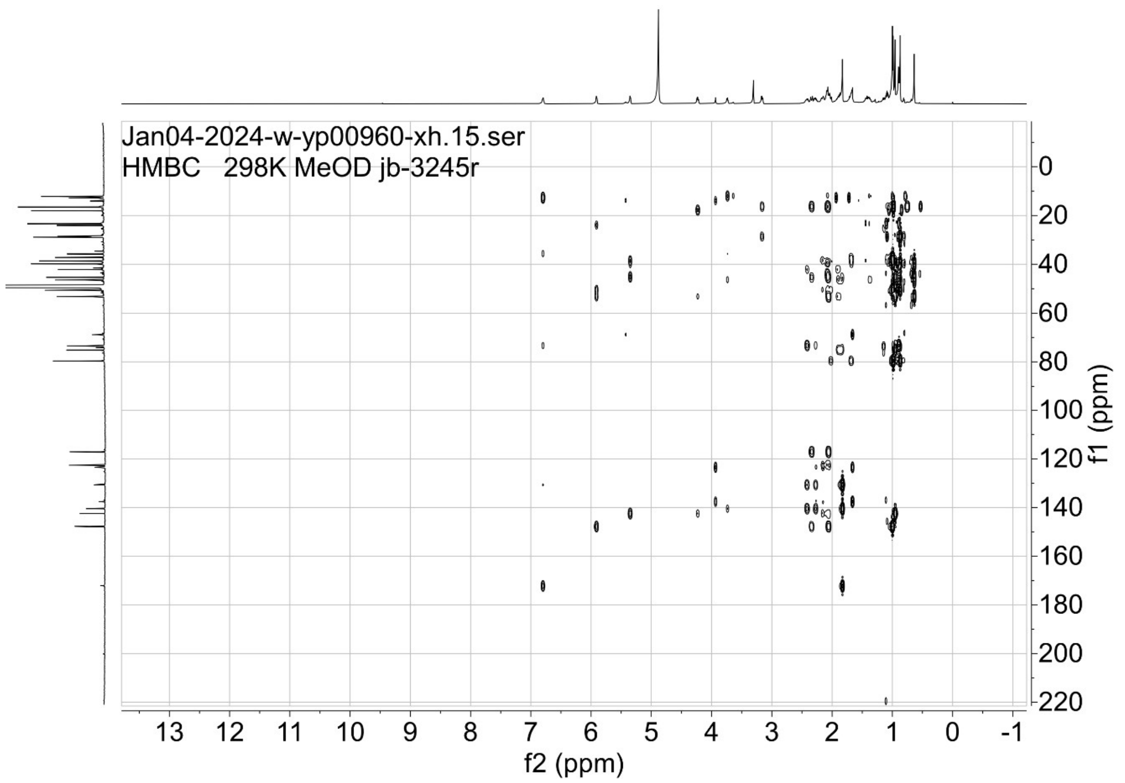


**Supplementary Fig. S50: HMBC spectrum of 10.**

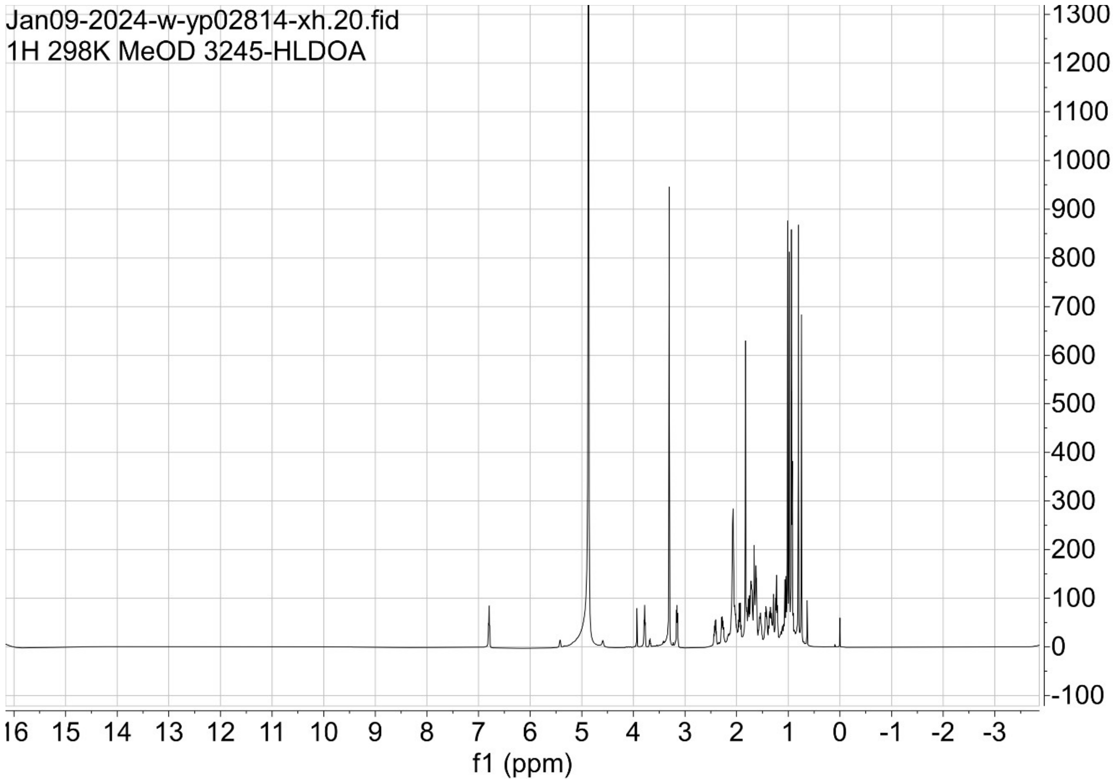


**Supplementary Fig. S51: $^1$H NMR spectrum of 12.**

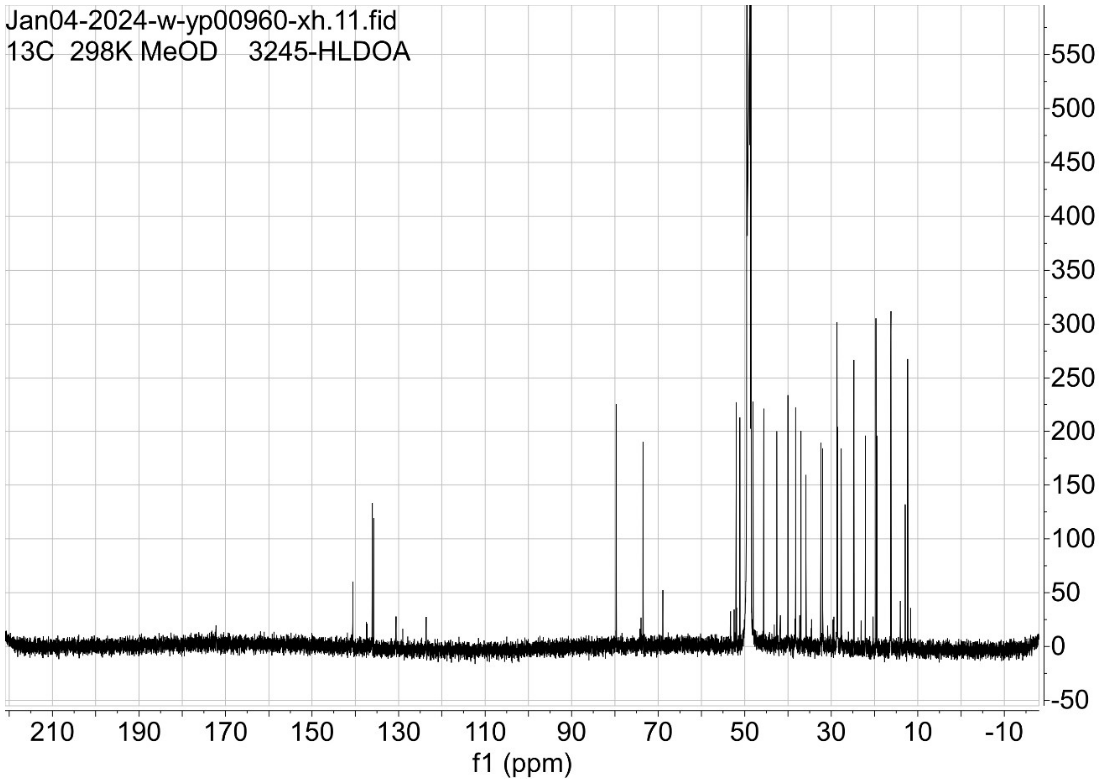


**Supplementary Fig. S52: $^{13}C$ NMR spectrum of 12.**

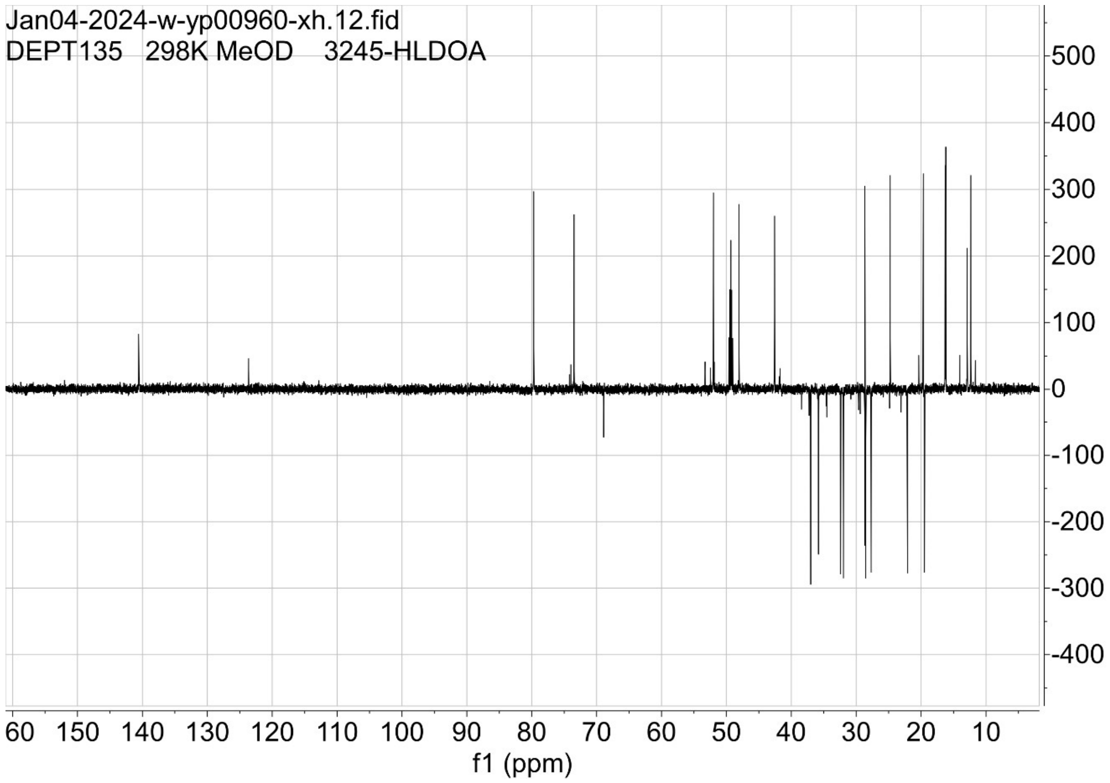


**Supplementary Fig. S53: DEPT-135 spectrum of 12.**

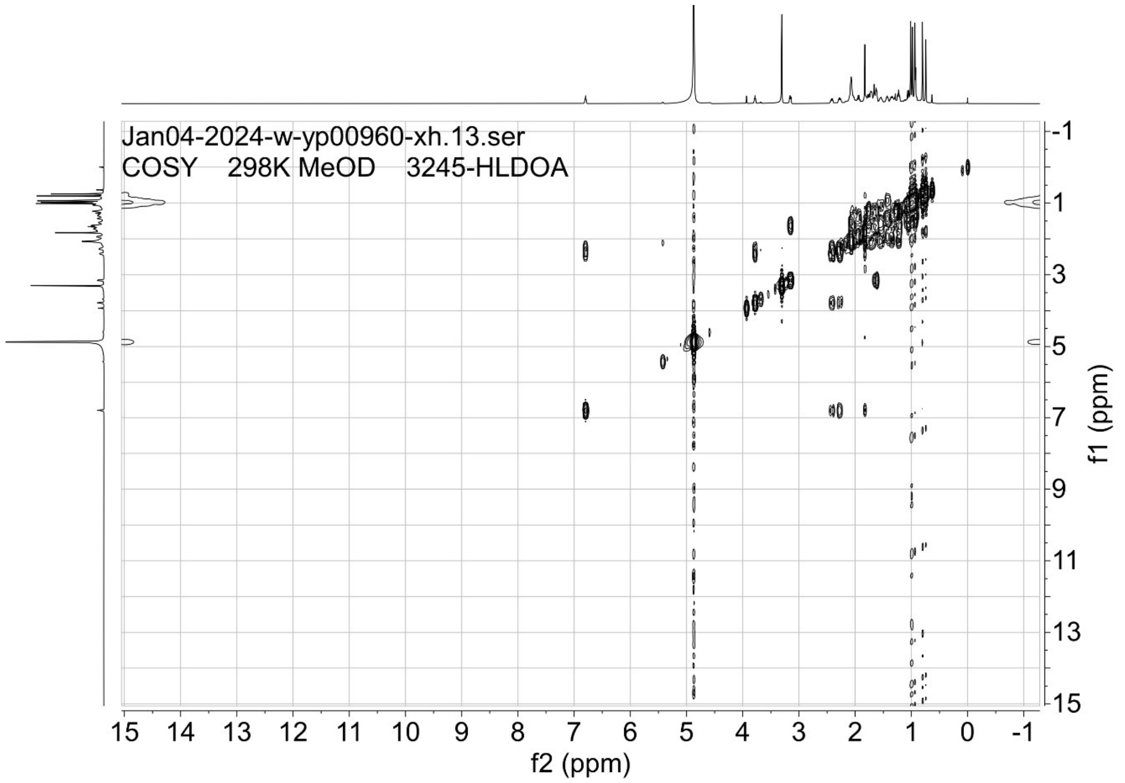


**Supplementary Fig. S54: COSY spectrum of 12.**

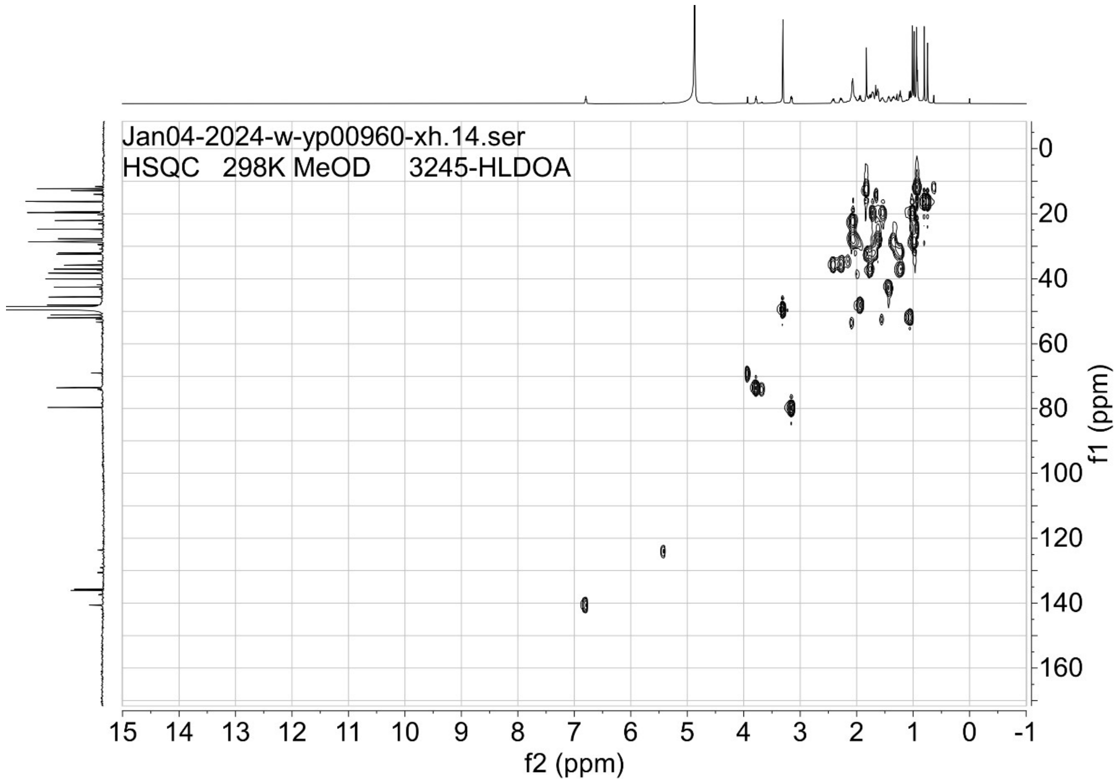


**Supplementary Fig. S55: HSQC spectrum of 12.**

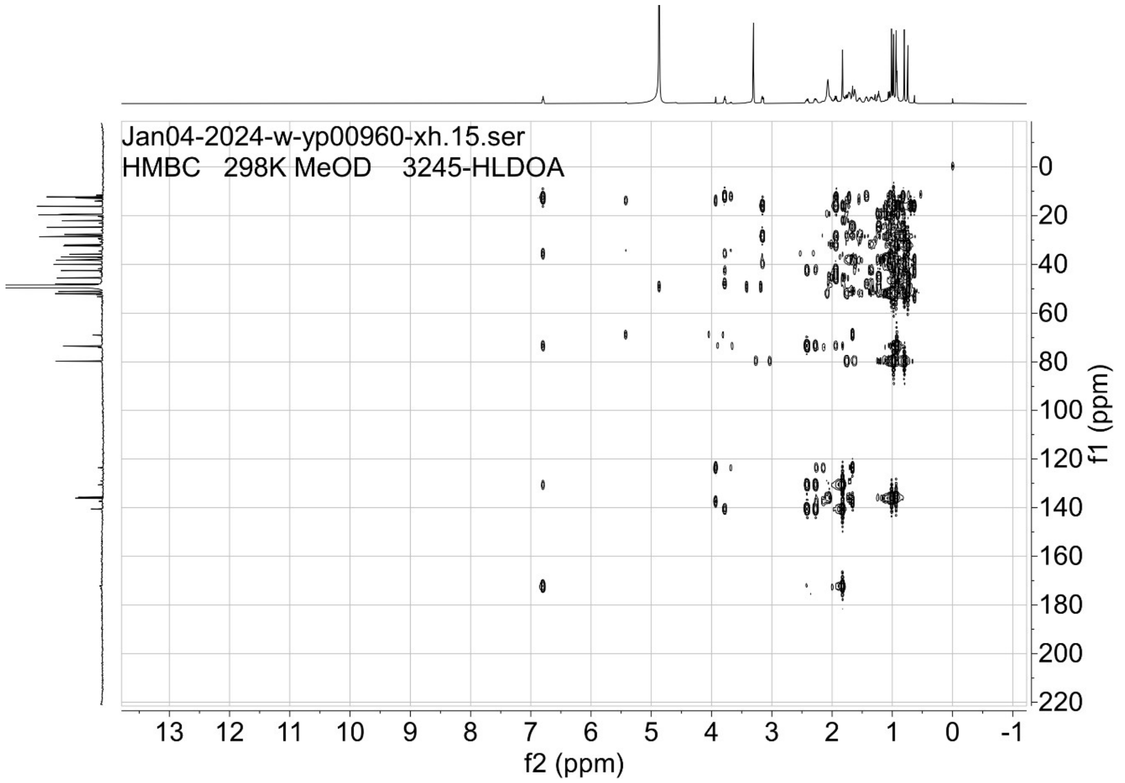


**Supplementary Fig. S56: HMBC spectrum of 12.**

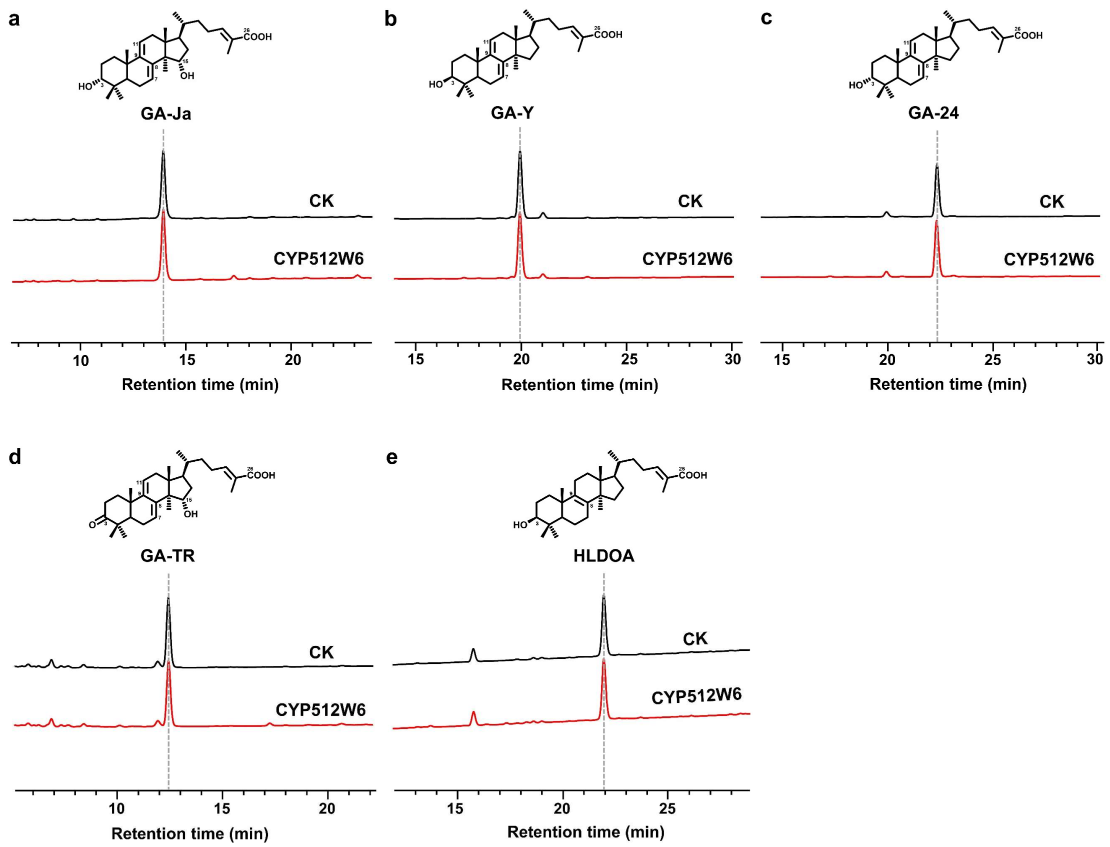


**Supplementary Fig. S57: *In vitro* enzymatic reaction of CYP512W6 with different GAs.** HPLC analysis of *in vitro* reaction extracts by incubation of CYP512W6 containing microsomes with GA-Ja (**a**), GA-Y (**b**), GA-24 (**c**), GA-TR (**d**), and HLDOA (**e**). CK indicates microsomes prepared from the control strain CK-r-iGLCPR-r, CYP512W6 indicates microsomes prepared from the strain CYP512W6-r-iGLCPR-r.

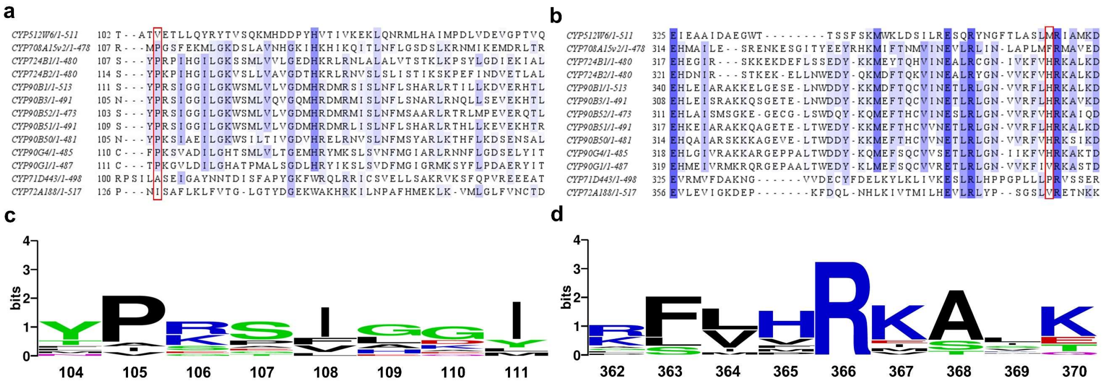

**Supplementary Fig. S58: Sequence alignment of CYP capable of hydroxylating C22 of GA-like substrates.** The position number is assigned by CYP512W6. **a.** The sequences contain V105 in 13 CYPs (highlighted in red square). **b.** The sequences contain M365 in 13 CYPs (highlighted in red square). Sequence logo of 12 heterologous CYPs contain V105 (**c**) and M365 (**d**) corresponding to CYP512W6.

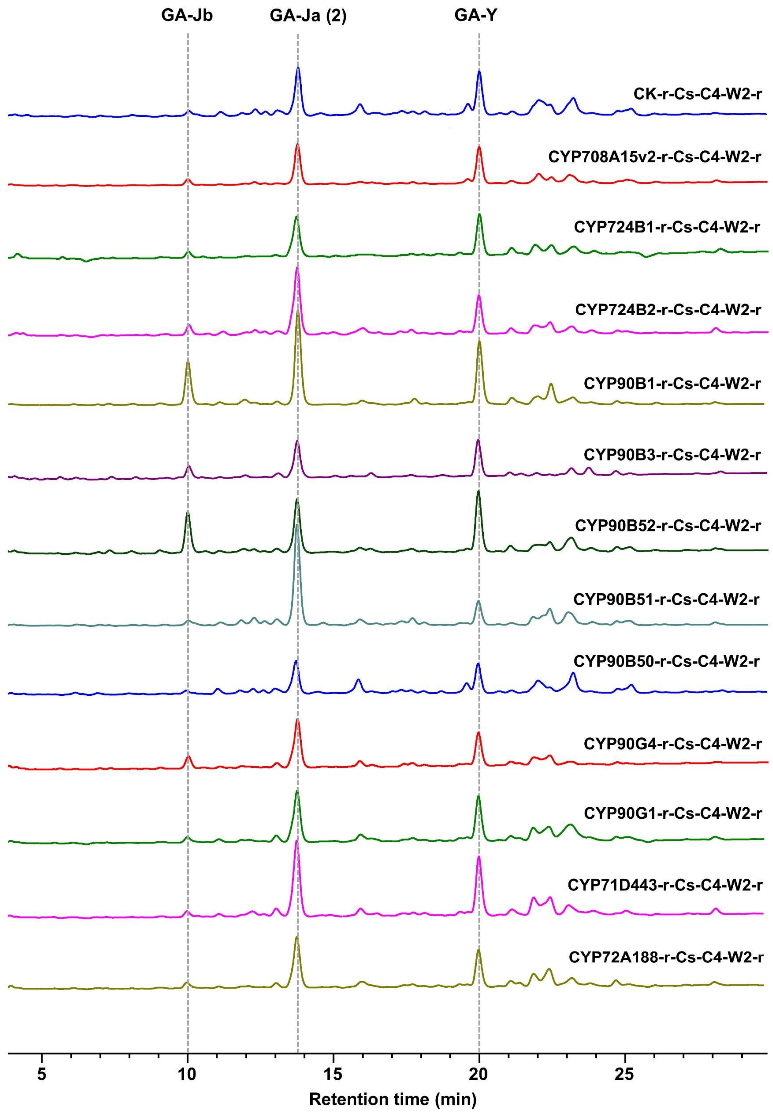


**Supplementary Fig. S59: HPLC analysis of the fermentation extracts of strains expressing heterologous CYPs.** CK-r-Cs-C4-W2-r (SC62-CK-r-CsSDR-AKR1C4-CYP512W2-r), and CYP(s)-r-Cs-C4-W2-r (SC62-CYP(s)-r-CsSDR-AKR1C4-CYP512W2-r).

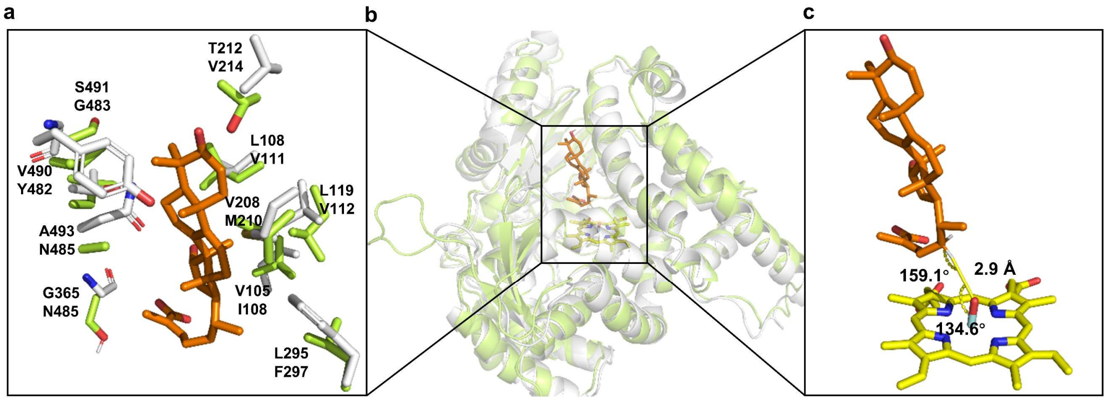


**Supplementary Fig. S60: The predicted structure of CYP512W6. a.** Residue around GA-Jb. The residue of CYP512W6 is shown on the upper part, while CYP512W2 is shown on the lower part. **b.** The comparison between the structures of CYP512W6 (limon) and CYP512W2 (grey). **c.** GA-Jb is docked to CYP512W2 at the PRS. GA-Jb and heme are represented as orange and yellow sticks, respectively.

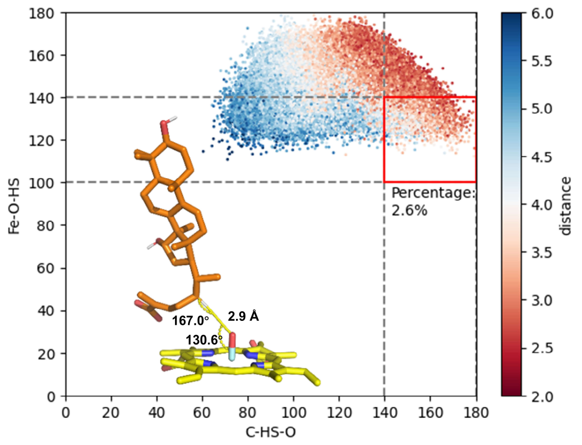


**Supplementary Fig. S61: The percentage of PRS of hydroxylation at C22 of GA-Jb in CYP512W6.** One snapshot of MD trajectories that satisfies the PRS requirement is shown. Heme and GA-Jb are shown as yellow and orange sticks, respectively.

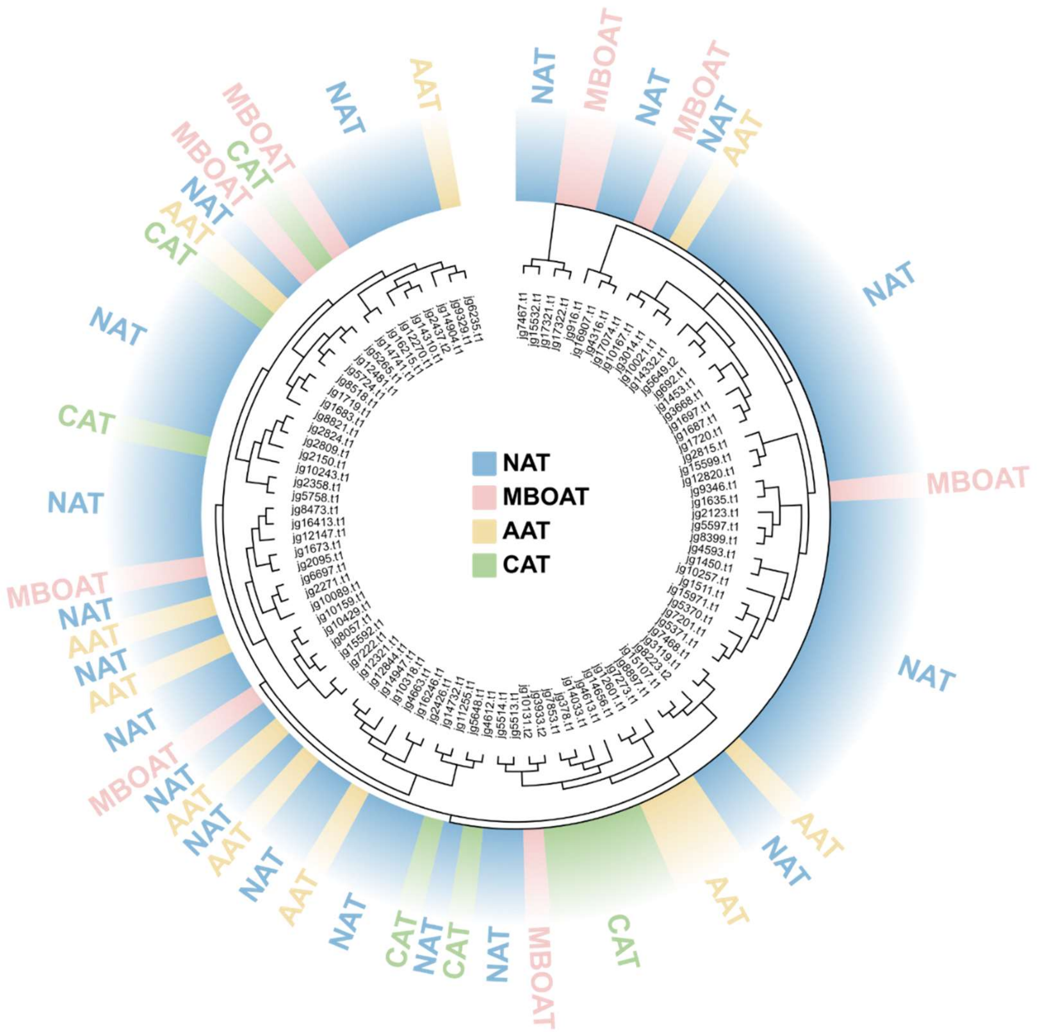


**Supplementary Fig. S62: Phylogenetic tree of 96 predicted acetyltransferases in *G. lucidum*.**

Amino acid sequences of acetyltransferase are aligned by using ClustalW as implemented in MEGA11. The evolutionary history is inferred by using the neighbor-Joining method based on the p-distance model. Evolutionary analyses are conducted in MEGA11 with 1000 bootstrap replicates. NAT, N-acetyltransferase, MBOAT, membrane-bound O-acyltransferase, AAT, alcohol acetyltransferase, CAT, chloramphenicol acetyltransferase.

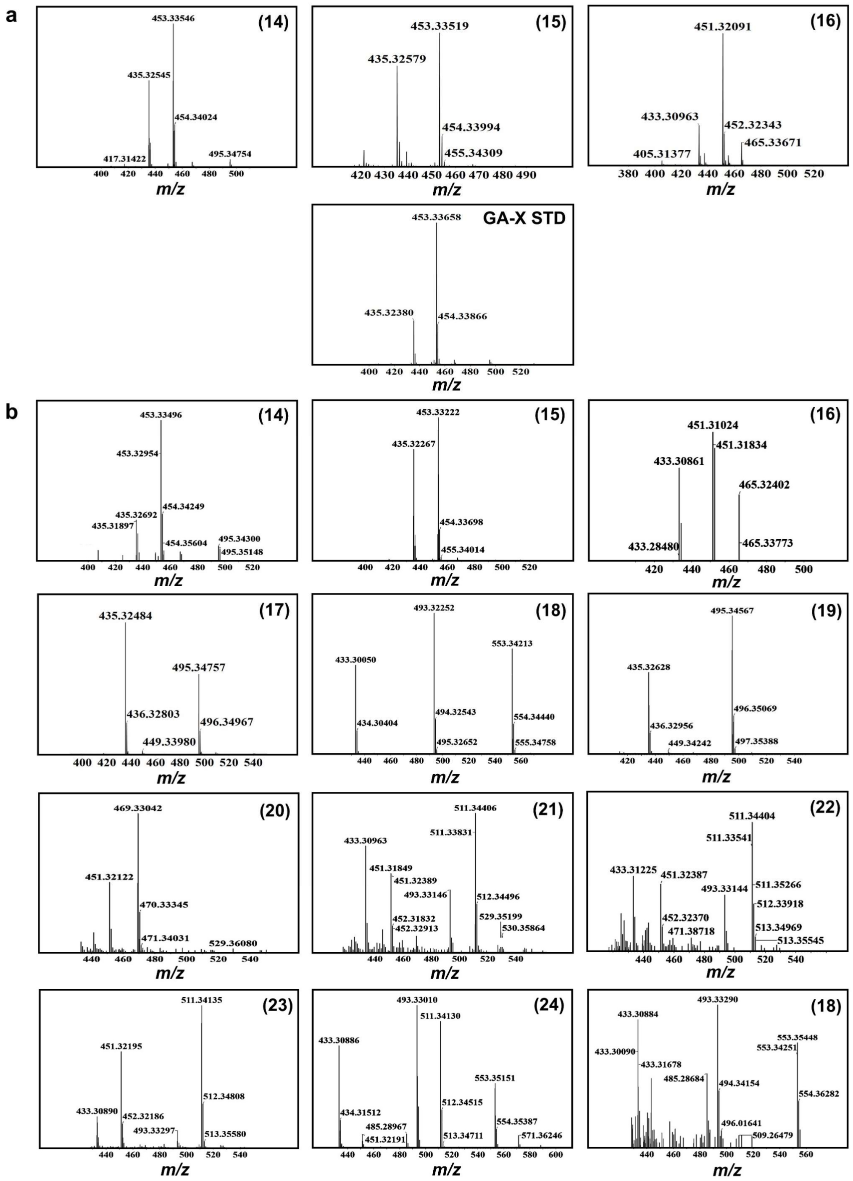


**Supplementary Fig. S63: Mass spectra of peaks 14-24 and the authentic standard of GA-X.**

**a.** Peaks of the fermentation extracts of GlAT expressing yeast strains. **b.** Peaks of *in vitro* enzymatic reaction extracts of GlAT.

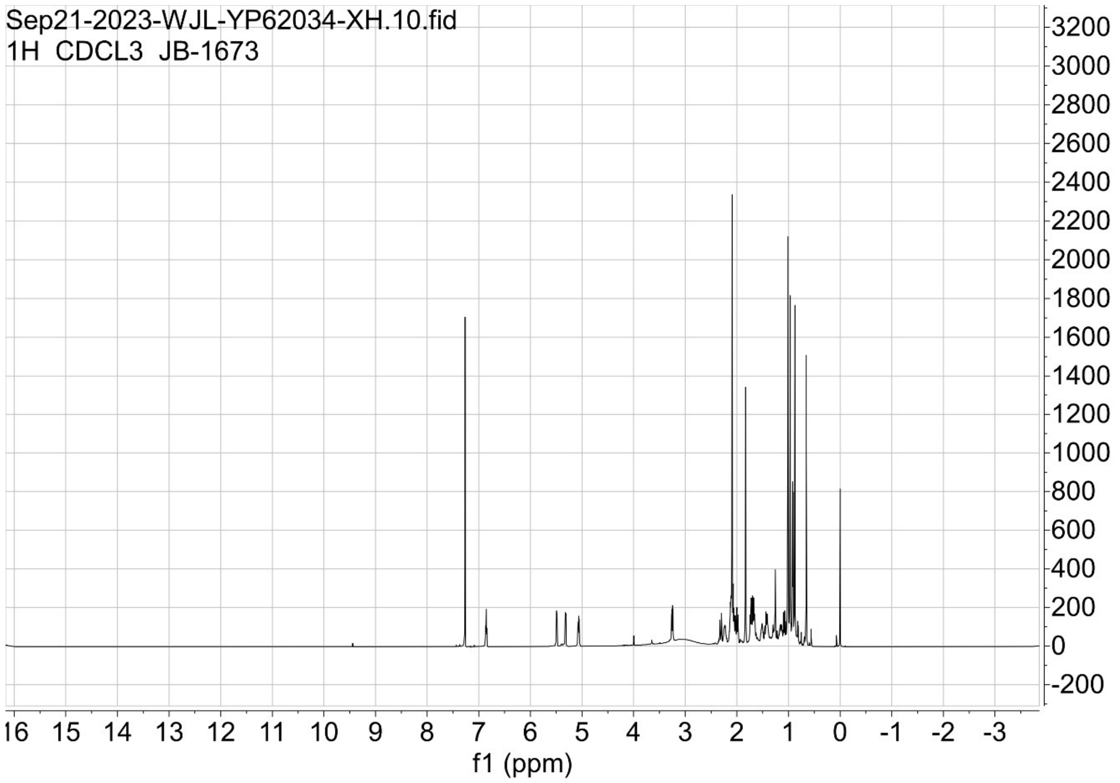


**Supplementary Fig. S64: $^{1}$H NMR spectrum of 14.**

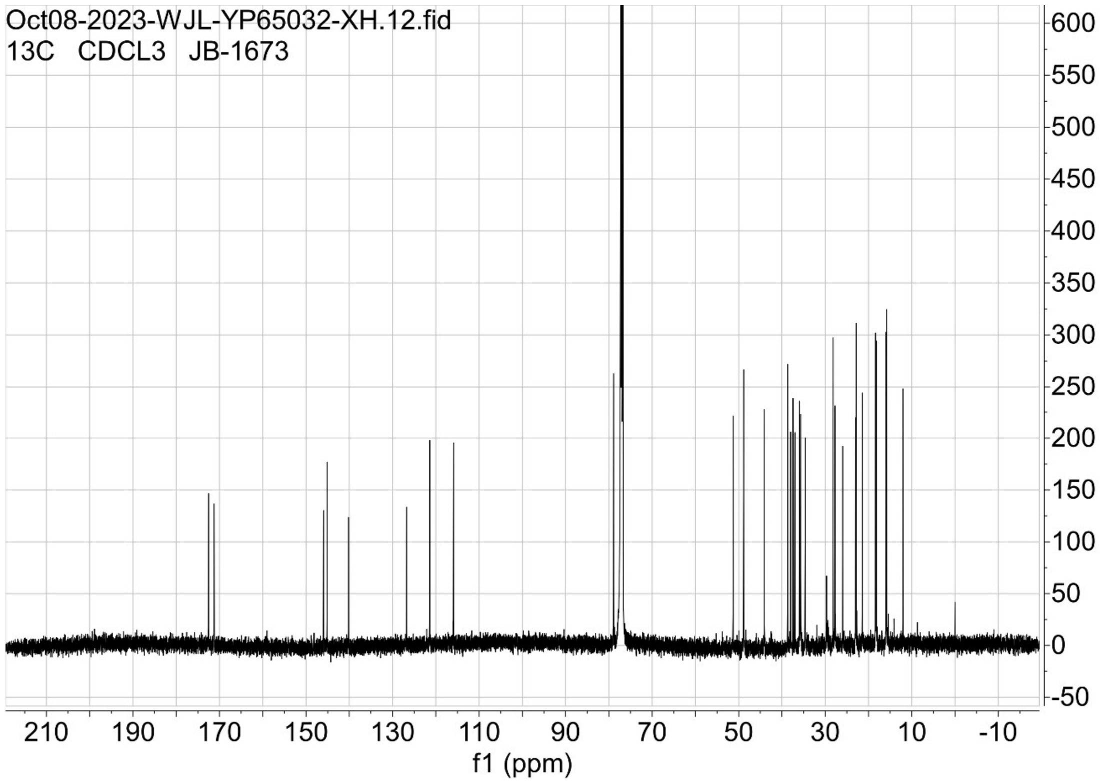


**Supplementary Fig. S65: $^{13}$C NMR spectrum of 14.**

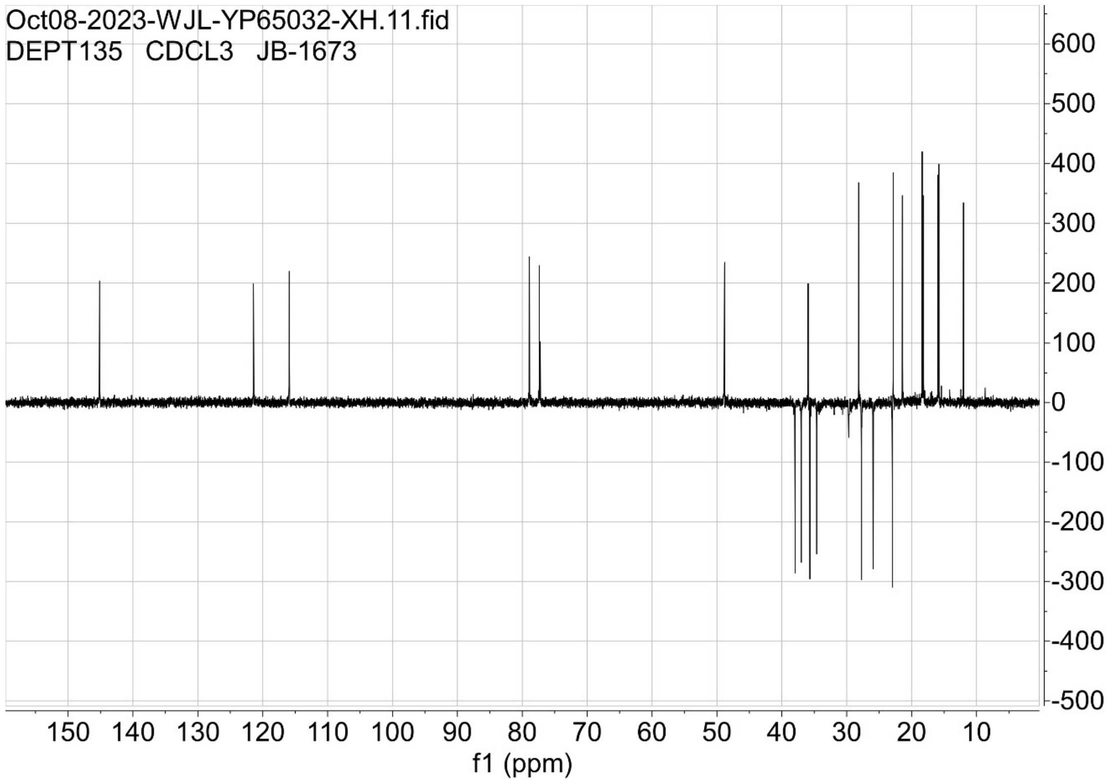


**Supplementary Fig. S66: DEPT-135 spectrum of 14.**

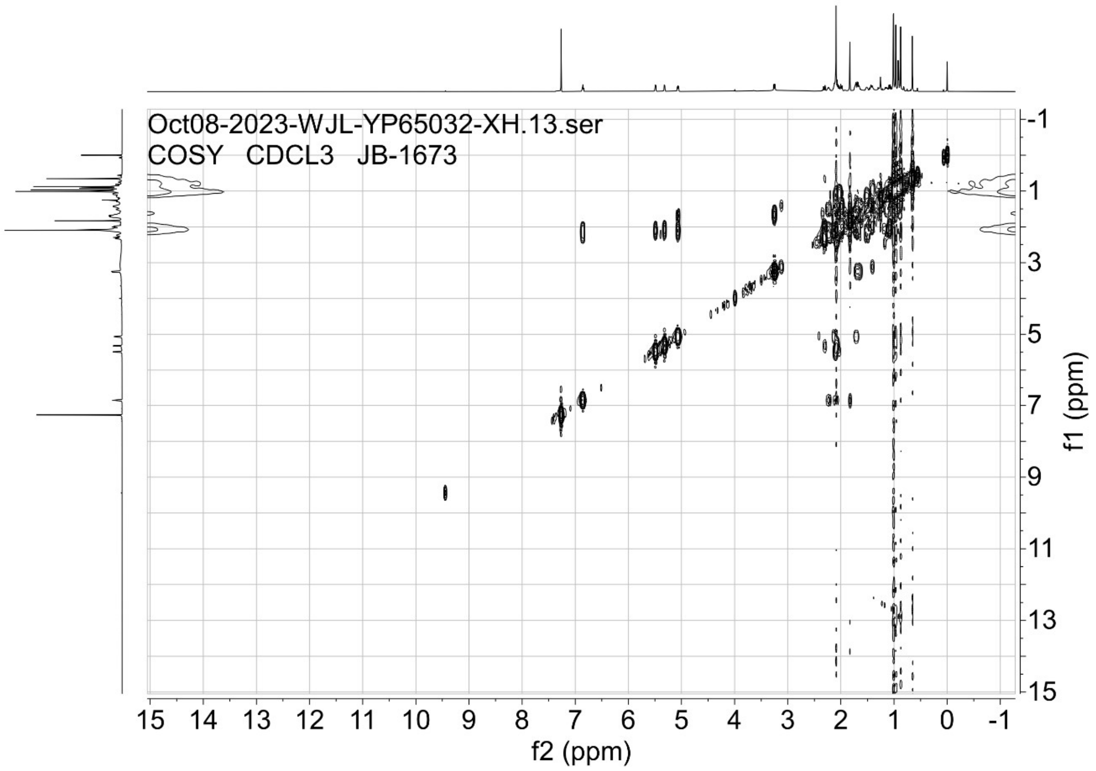


**Supplementary Fig. S67: COSY spectrum of 14.**

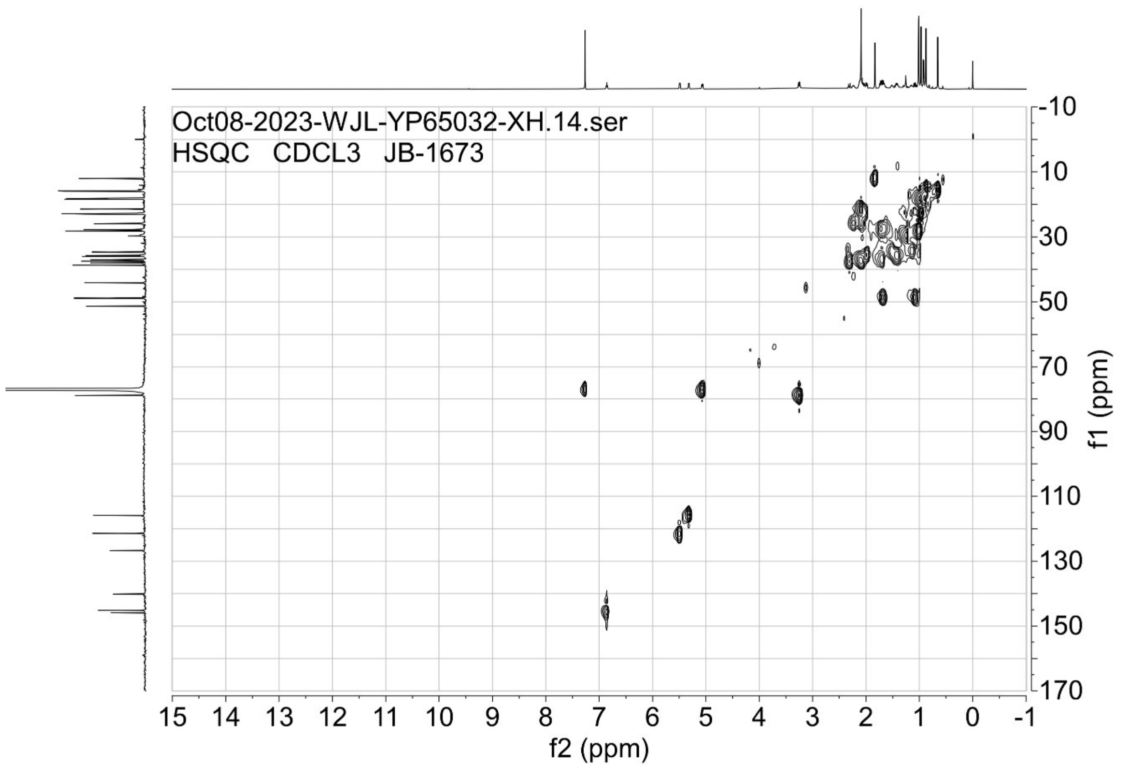


**Supplementary Fig. S68: HSQC spectrum of 14.**

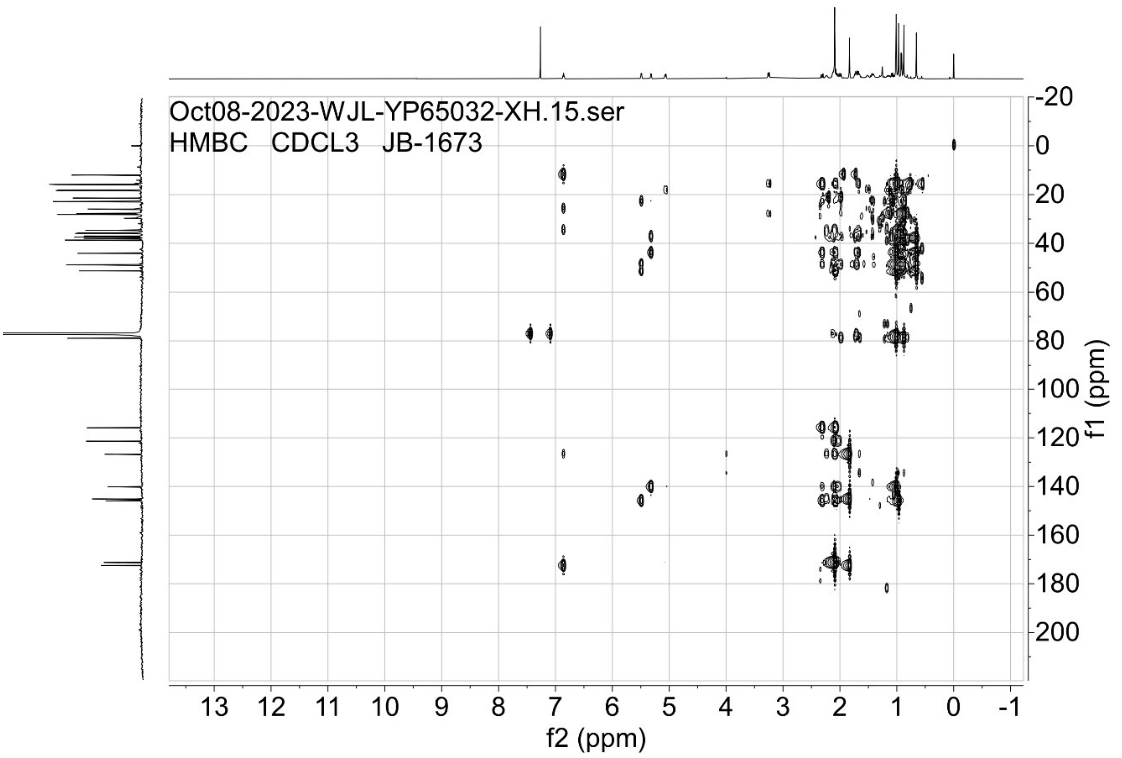


**Supplementary Fig. S69: HMBC spectrum of 14.**

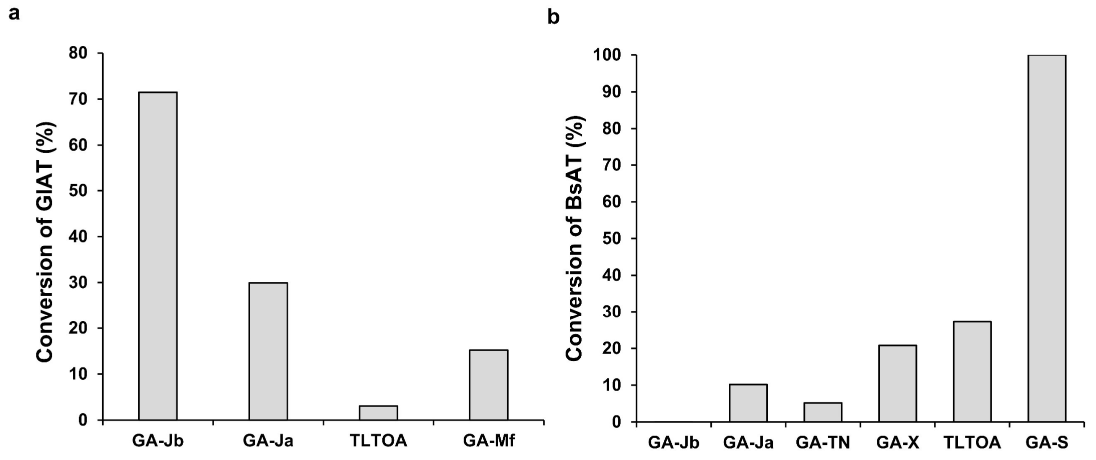


**Supplementary Fig. S70: The conversion of different substrates by GlAT (a) and BsAT (b).**

The conversion refers to the ratio of consumed substrate (the difference of the substrate concentration before and after the substrate is added) to the total amount of substrate.

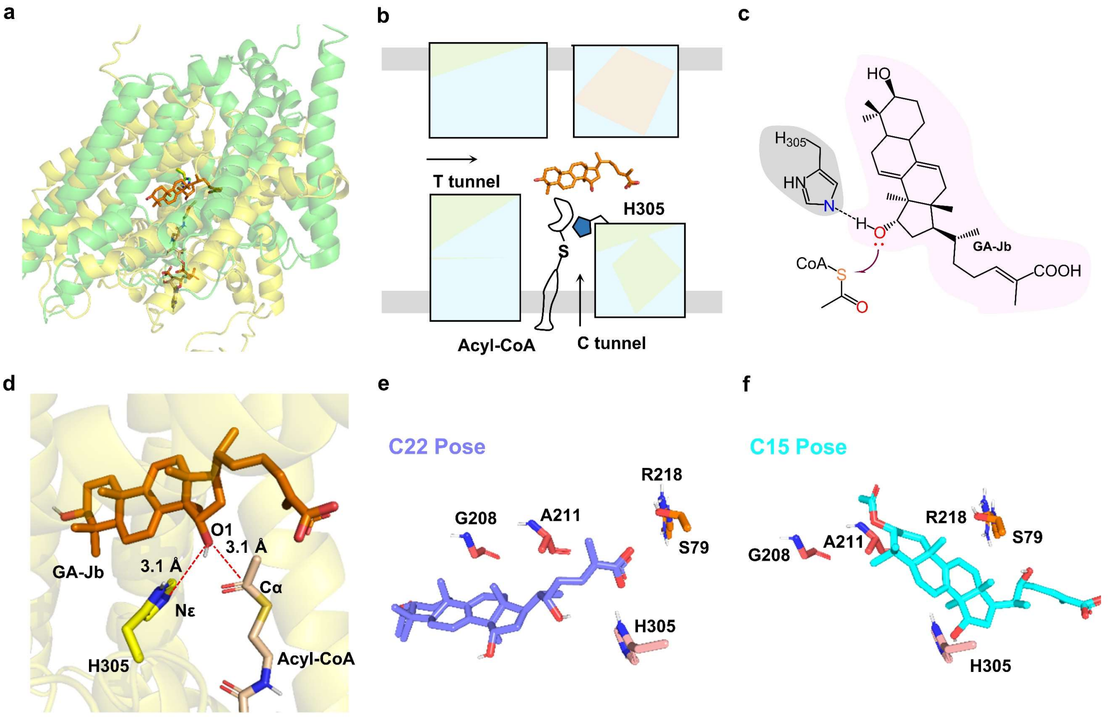


**Supplementary Fig. S71: The predicted structure of GlAT. a.** Comparison of GlAT (yellow) and ACAT1(green). GIAT1 with GA-Jb (orange) and acyl-CoA (wheat). **b.** The schematic working model of GlAT illustrates that the T-tunnel and the C-tunnel serve as entrances for GA-Jb and acyl-CoA, respectively. The reaction is catalyzed at the intersection of these two tunnels, where the active residue H305 is located. **c.** The catalytic mechanism for transfer of acetyl group. **d.** GA-Jb (orange stick) with acyl-CoA (wheat stick) and GlAT (yellow cartoon). Key distance is labeled as dotted lines. **e.** GA-T2 (slate) with GlAT at C22 pose. **f.** GA-T2 (cyan) with GlAT at C15 pose.

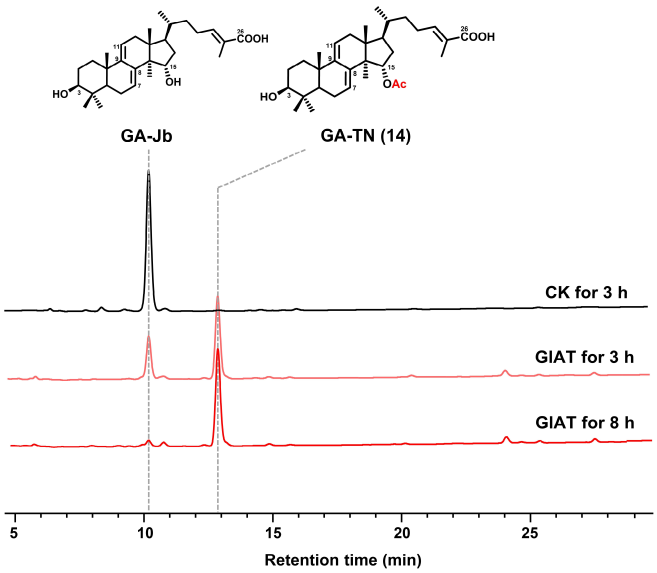


**Supplementary Fig. S72: HPLC analysis of the reaction extracts of strain BJ5464-GlAT feeding with GA-Jb for different times.** CK refers to the control strain BJ5464-CK-r. GlAT refers to the strain BJ5464-GlAT-r.

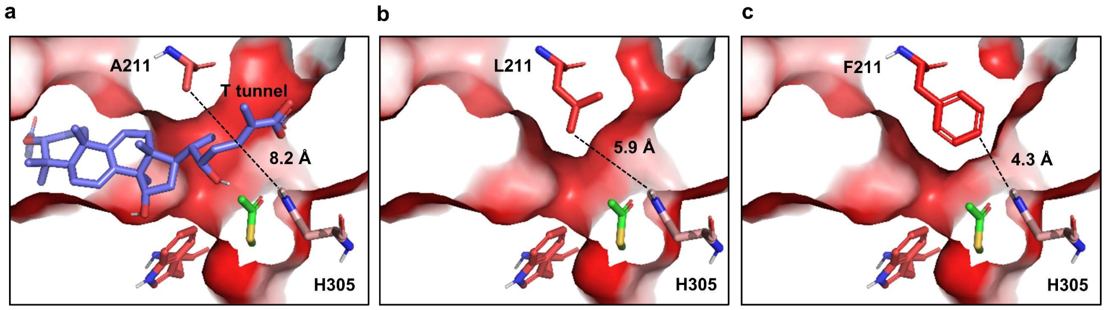


**Supplementary Fig. S73: Molecular docking of GlAT and its mutants with GA-T2. a.** T tunnel size of GlAT complexed with GA-T2 (slate) and acyl-CoA (green). **b.** T tunnel size of A211L. **c.** T tunnel size of A211F.

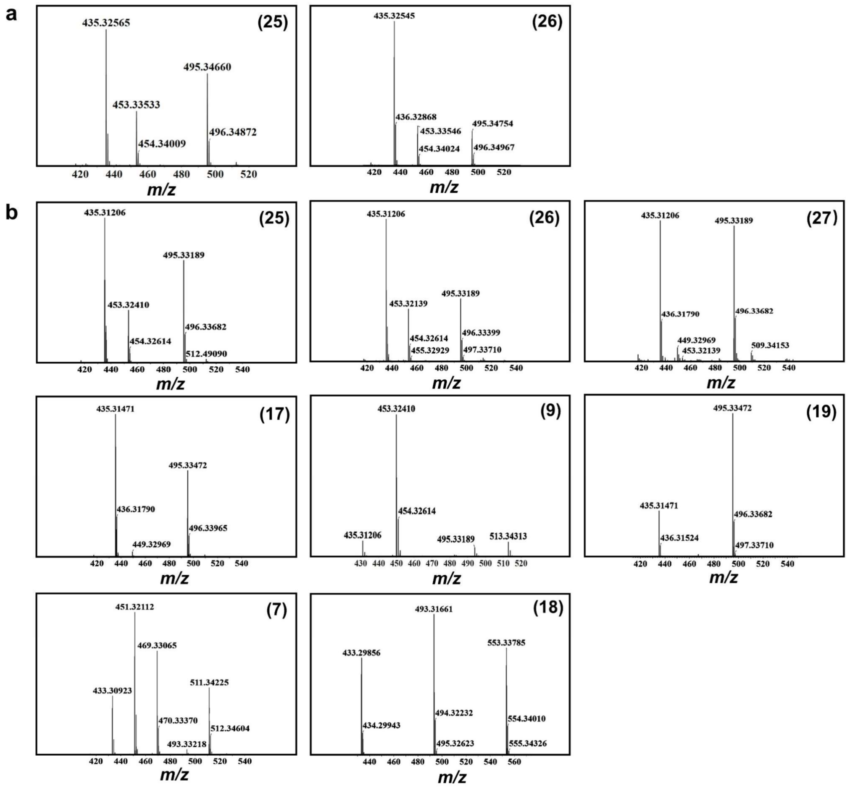


**Supplementary Fig. S74: Mass spectra of peaks 25-27, 17, 9, 19, 7, and 18. a.** Peaks of the fermentation extracts from BsAT expressing yeast strains. **b.** Peaks of *in vitro* enzymatic reaction extracts of BsAT.

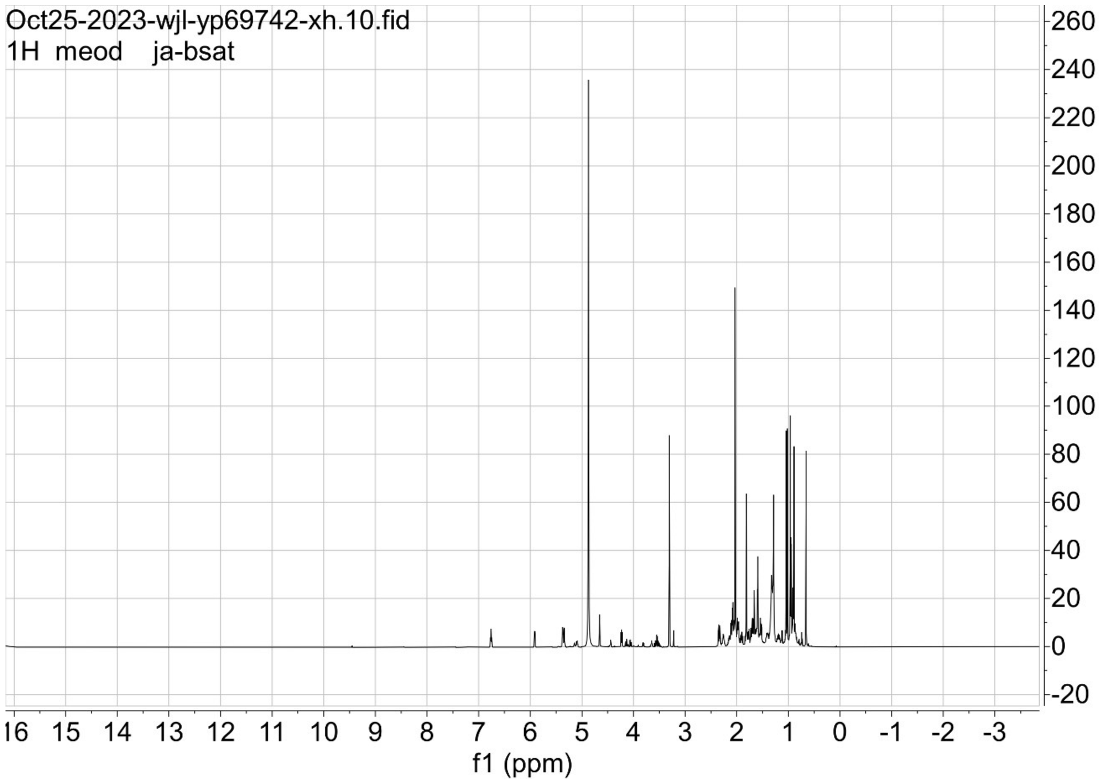


**Supplementary Fig. S75: $^{1}$H NMR spectrum of 26.**

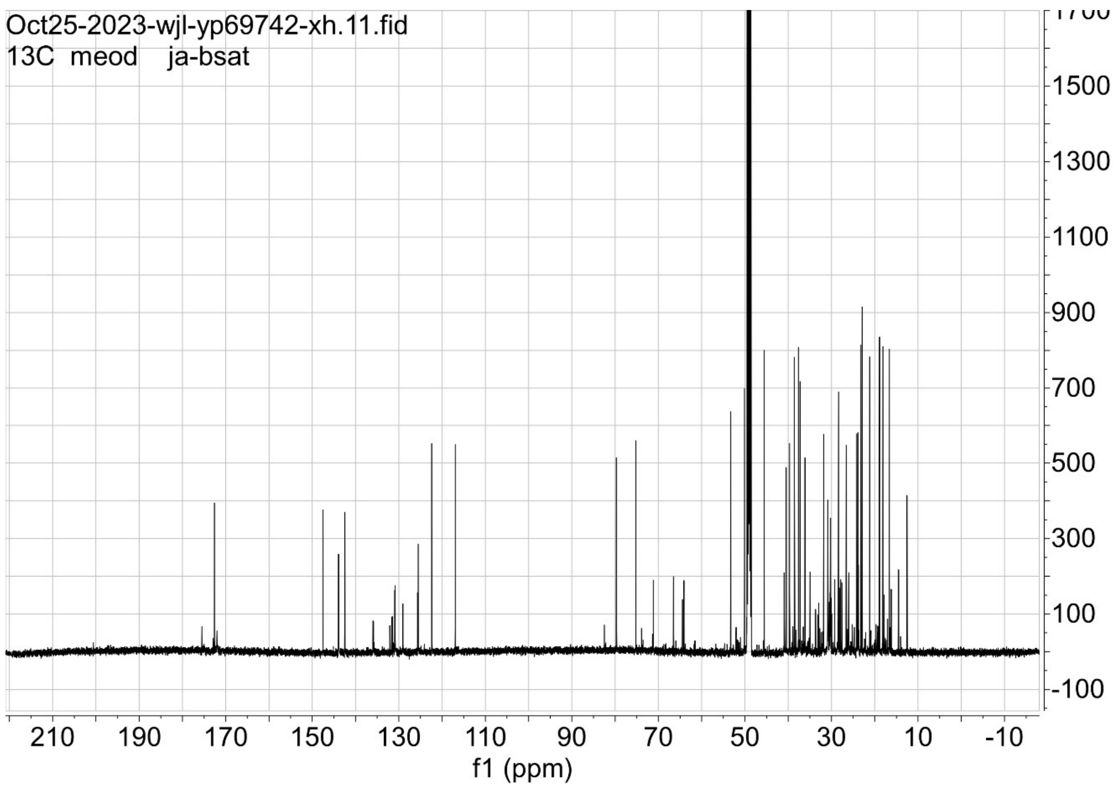


**Supplementary Fig. S76: $^{13}$C NMR spectrum of 26.**

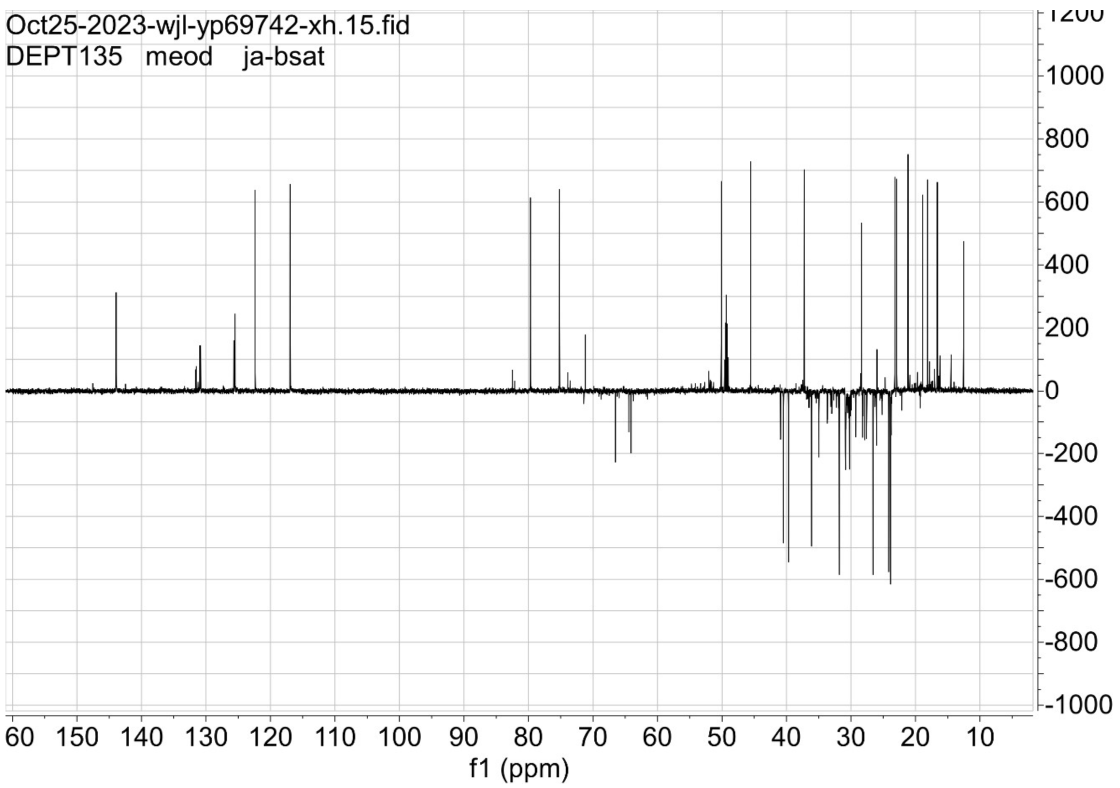


**Supplementary Fig. S77: DEPT-135 spectrum of 26.**

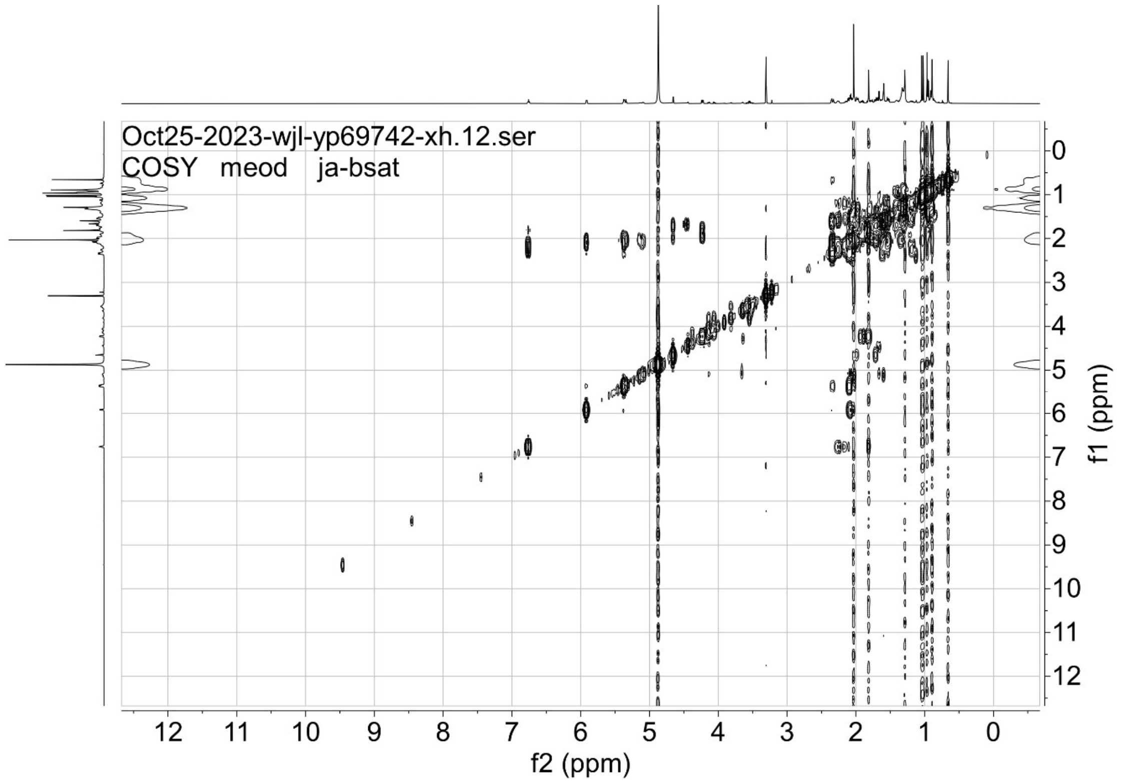


**Supplementary Fig. S78: COSY spectrum of 26.**

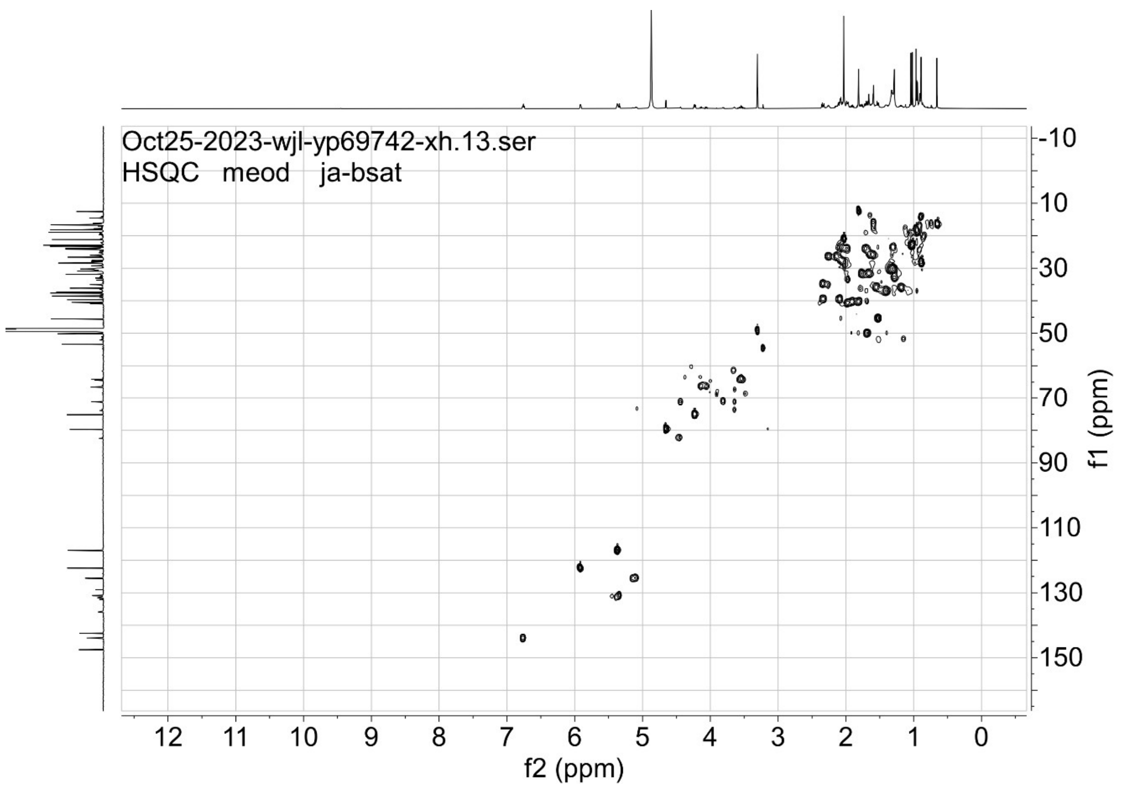


**Supplementary Fig. S79: HSQC spectrum of 26.**

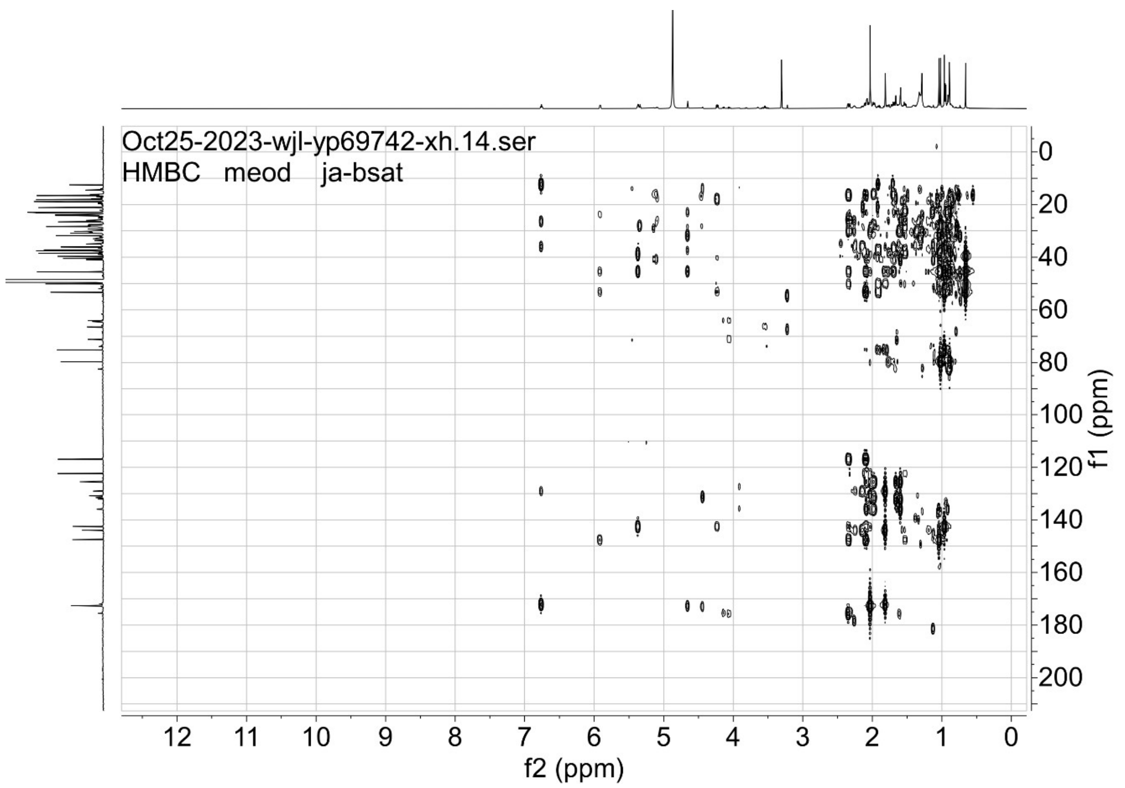


**Supplementary Fig. S80: HMBC spectrum of 26.**

a

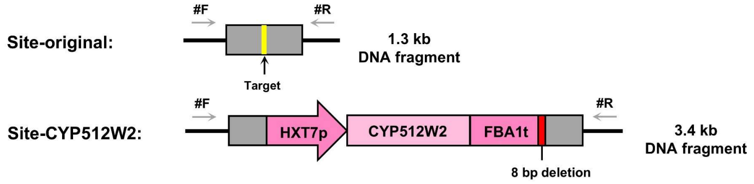


b

| Site Name | Chromosomal Coordinates | Genes flanking the intergenic region | 20 bp gRNA sequence |
|---|---|---|---|
| H1 | ChrXV: 160595–161578 | ADH1/YOL085W-A | CCAATGCTAGTAGAGAAGGG |
| H2 | ChrXII: 234082–235036 | PDC1/STU2 | GAGATAGGTAAATAAACGCG |

c

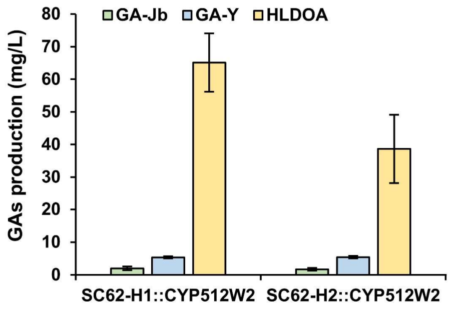


**Supplementary Fig. S81: Single copy integration of the expression cassettes of CYP512W2. a.** Overview of the design and detection of the engineered strains. **b.** Integration loci and the corresponding gRNA sequences. **c.** Production of GA-Jb, GA-Y, and HLDOA after 120 h fermentation of the engineered yeast strains. All data represent the mean of three independent samples, the error bars indicate the standard deviation.

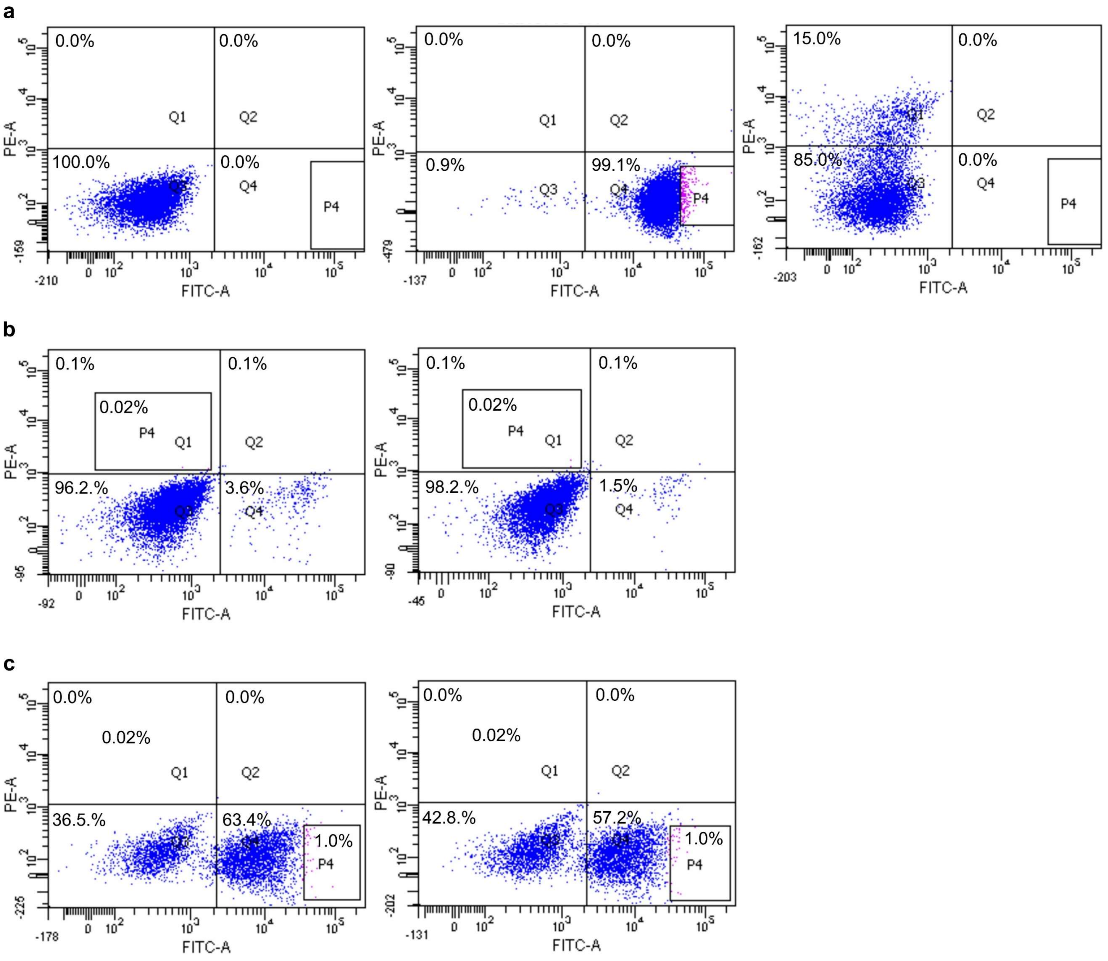


**Supplementary Fig. S82: FACS analysis of engineered strains with integration of key enzyme expression cassettes.** The red and green fluorescent signals of strains BJ5464 (left), SC62 (middle) and YL-T3-RFP (right) (**a**), SC62 integrated with CYP512W2$^{I108A}$ and RFP expression cassettes in the site of EGFP-122 bp (left) and 309 bp (right) (**b**), SC27 integrated with CsSDR, AKR1C4 and EGFP$^{syn-mutant}$ (left) or CYP512W6, CsSDR, AKR1C4 and EGFP$^{syn-mutant}$ (right) expression cassettes in the RFP-191 bp site (**c**).

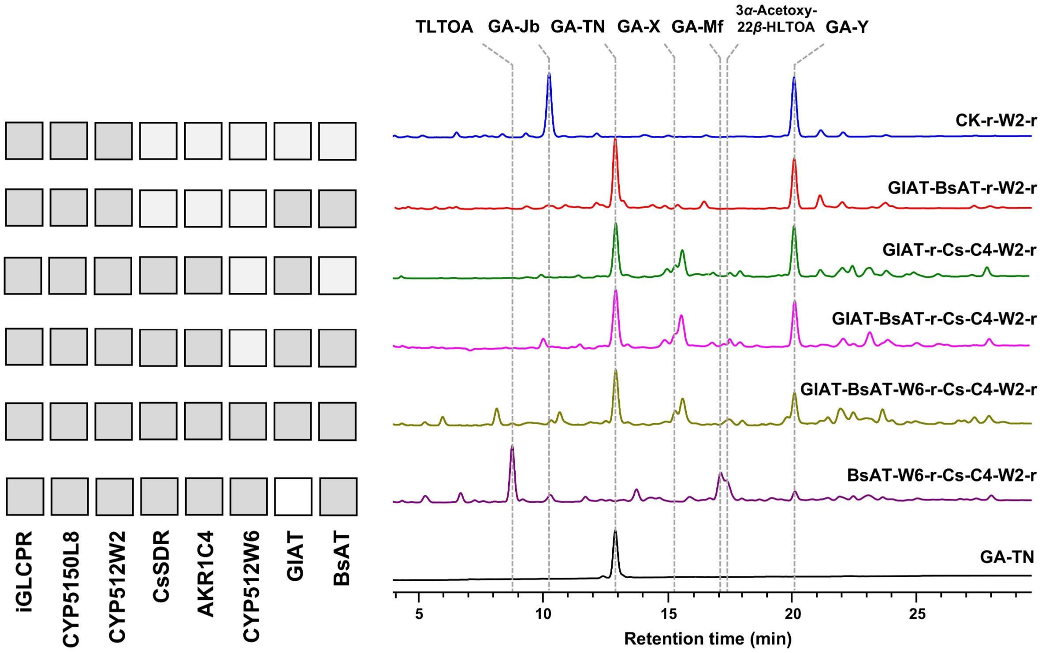


**Supplementary Fig. S83: Production of TIIGAs by constitutive expression of identified genes.**

HPLC analysis of the fermentation extracts of strains SC62-CK-r-CYP512W2-r (CK-r-W2-r), SC62-GlAT-BsAT-r-CYP512W2-r (GlAT-BsAT-r-W2-r), SC62-GlAT-r-CsSDR-AKR1C4-CYP512W2-r (GlAT-r-Cs-C4-W2-r), SC62-GlAT-BsAT-r-CsSDR-AKR1C4-CYP512W2-r (GlAT-BsAT-r-Cs-C4-W2-r), SC62-GlAT-BsAT-CYP512W6-r-CsSDR-AKR1C4-CYP512W2-r (GlAT-BsAT-W6-r-Cs-C4-W2-r), and SC62-BsAT-CYP512W6-r-CsSDR-AKR1C4-CYP512W2-r (BsAT-W6-r-Cs-C4-W2-r).

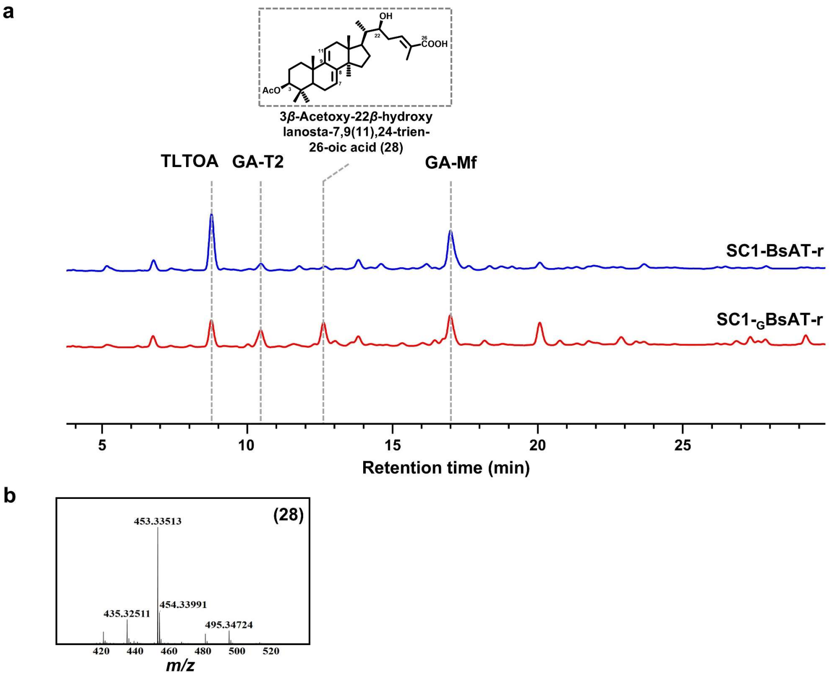


**Supplementary Fig. S84 Production of TIIGAs by regulating the expression of BsAT. a.** HPLC analysis of the fermentation extracts by SC1-BsAT-r and SC1-P$_{GAL1}$BsAT-r (SC1-$_{G}$BsAT-r) after 168 h fermentation. **b**. MS spectra of peak 28. The chemical structure of compound, highlighted by a dotted line frame, was inferred from the mass spectrum signal. For the chemical structure speculation, see Supplementary Note S2.

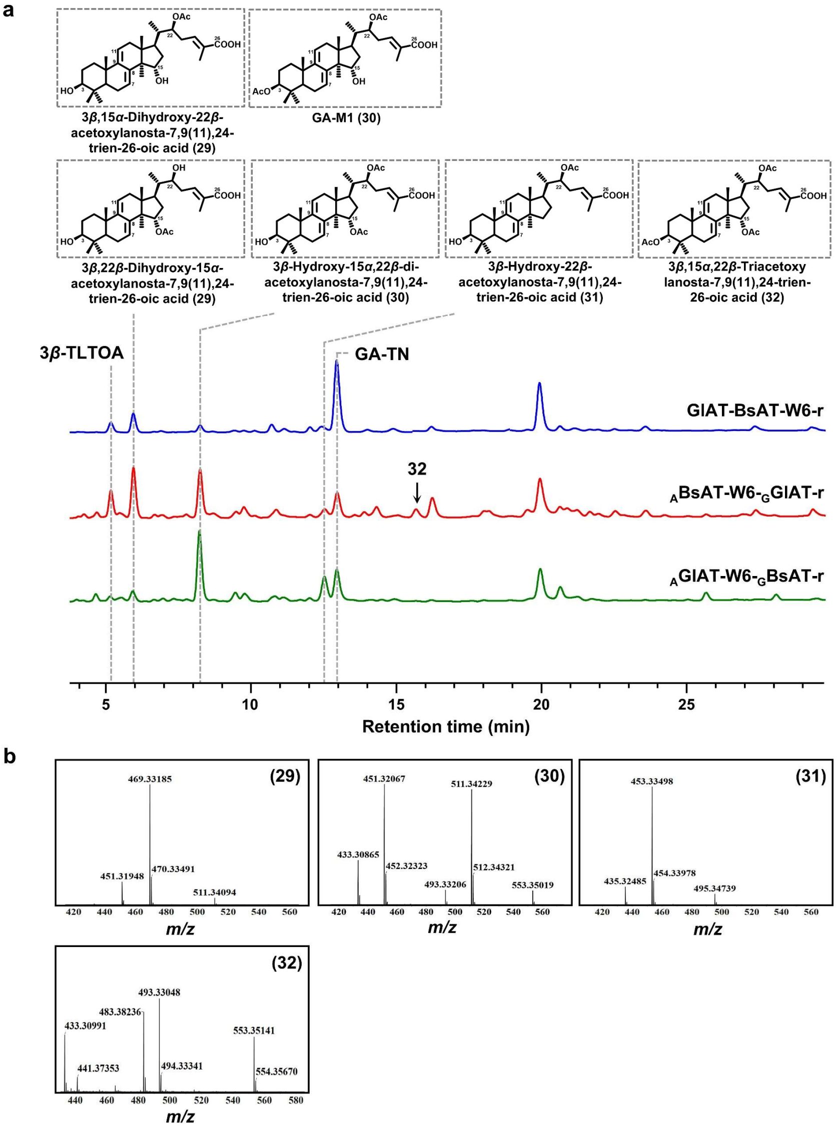


**Supplementary Fig. S85: Production of C3 configuration unconverted TIIGAs by regulating the expression of acetyltransferases. a.** HPLC analysis of the fermentation extracts by SC27-GlAT-BsAT-CYP512W6-r (GlAT-BsAT-W6-r), SC27-P$_{ADH2}$BsAT-CYP512W6-P$_{GAL1}$GlAT-r ($_A$BsAT-W6-$_G$GlAT-r), and SC27-P$_{ADH2}$GlAT-CYP512W6-P$_{GAL1}$BsAT-r ($_A$GlAT-W6-$_G$BsAT-r) after 168 h fermentation. **b.** MS spectra of peaks **29-32**. The chemical structure of compounds, highlighted by the dotted line frames, were inferred from the mass spectrum signal. For the chemical structure speculation, see Supplementary Note S2.

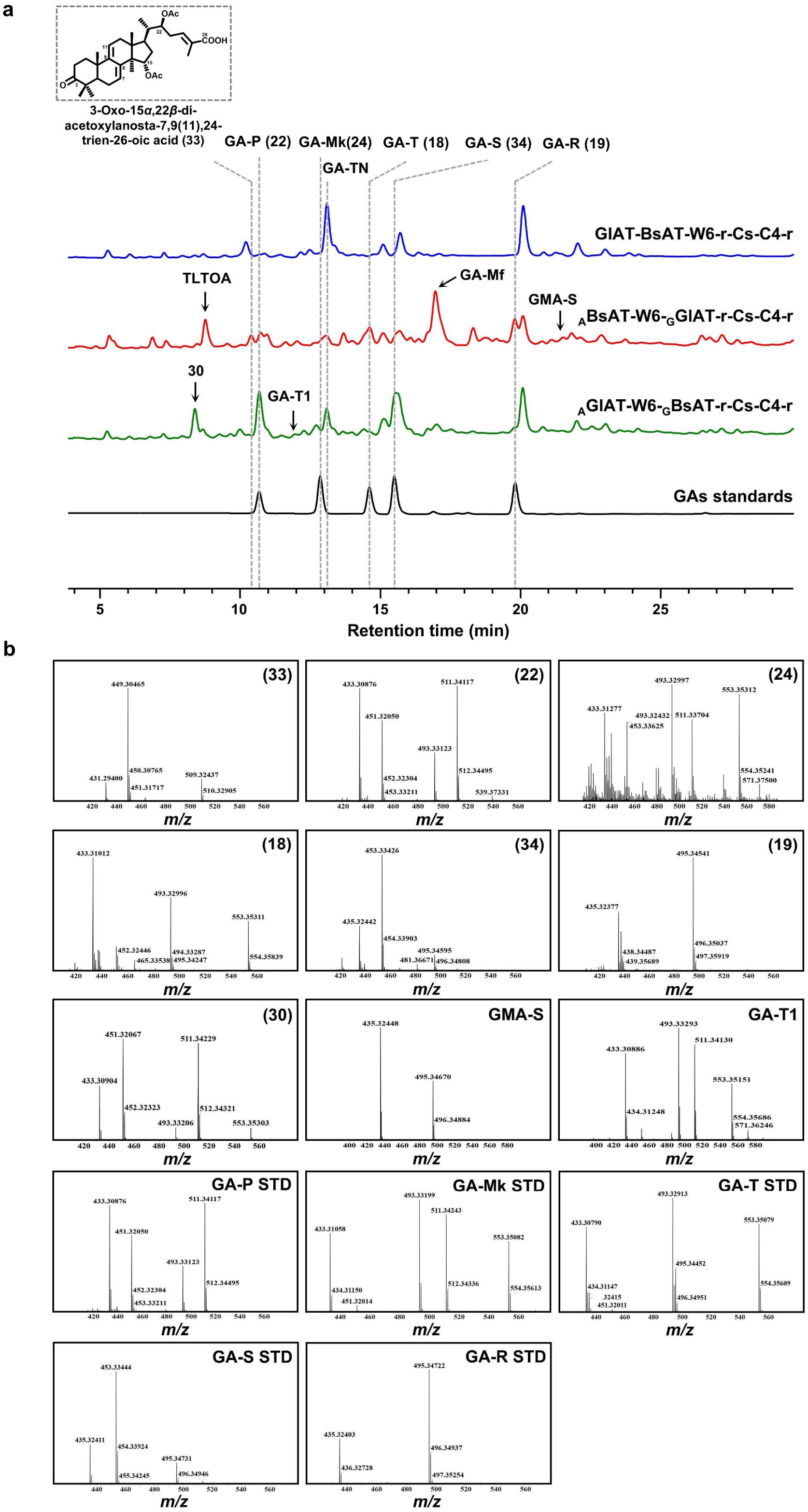


**Supplementary Fig. S86 Production of C3 configuration converted TIIGAs by regulating the expression of acetyltransferases. a.** HPLC analysis of the fermentation extracts by SC27-GlAT-BsAT-CYP512W6-r-CsSDR-AKR1C4-r (GlAT-BsAT-W6-r-Cs-C4-r), SC27-P*ADH2*BsAT-

CYP512W6-P$_{GAL1}$GlAT-r-CsSDR-AKR1C4-r ($_{A}$BsAT-W6-$_{G}$GlAT-r-Cs-C4-r) and SC27-P$_{ADH2}$GlAT-CYP512W6-P$_{GAL1}$BsAT-r-CsSDR-AKR1C4-r ($_{A}$GlAT-W6-$_{G}$BsAT-r-Cs-C4-r) after 168 h fermentation. **b.** MS spectra of peak **30**, **33**, GMA-S, GA-T1, GA-P, GA-Mk, GA-T, GA-S, GA-R and the authentic standards. The chemical structure of compounds, highlighted by the dotted line frames, were inferred from the mass spectrum signal. For the chemical structure speculation, see Note S2.

**Supplemental Table S1. Bioactivities of typical TIIGAs**

| Structures | Compounds | Biological activity | Activity index | Ref. |
|---|---|---|---|---|
| 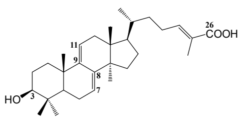 | GA-Y | Angiotensin-converting enzyme (ACE) inhibition | $IC_{50}$ = 43 μM | 1 |
| | | Acetylcholinesterase (AChE) inhibition | $IC_{50}$ = 21.11±2.66 μM | 2 |
| | | Cholesterol synthesis inhibition | $ID_{50}$ = 1.4 μM | 3 |
| | | 3-Hydroxy-3-methylglutaryl coenzyme A (HMG-CoA) reductase inhibition | $IC_{50}$ = 8.6±2.0 μM | 4 |
| | | Inhibiting the release of tumor necrosis factor-α (TNF-α), interleukin-1β (IL-1β) and interleukin-6 (IL-6), anti-inflammatory | NO release inhibition rate, % = 27.87±0.73; Inflammatory factor release, ng/mL = 211.92±4.8 (TNF-α); 101.6±22.62 (IL-1β); 58.62±5.16 (IL-6) | 5 |
| | | Cytotoxicity against cancer cell lines HeLa (human cervical cancer); K562 (human myelogenous leukemia); PC-3 (human prostate cancer); H460 (lung cancer); HCT116 (colorectal carcinoma); BGC823 (gastric cancer); SMMC-7721 (liver cancer) and A549 (human lung cancer) | $IC_{50}$, μM = 59 (HeLa); 17.5±4.5 (K562); 179.8±22.5 (PC-3); 22.4 (H460);47.9 (HCT116); 49.1 (BGC823); 33.51 (SMMC-7721); 29.94 (A549) | 4,6-8 |
| | | Antiviral effects against enterovirus 71 infection | - | 9 |
| 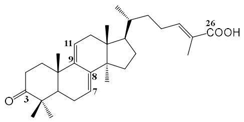 | 3-Oxo-lanosta-7,9(11),24-trien-26-oic acid | ACE inhibition | $IC_{50}$ = 95 μM | 1 |
| | | HMG-CoA reductase inhibition | $IC_{50}$ = 12.5±2.3 μM | 4 |
| | | Cytotoxicity against tumor cell lines | $IC_{50}$, μM =6 5.2±9.9 (K562); 75.2±17.9 (PC-3) | 4 |
| | | Brain-derived neurotrophic factor-like neuronal survival-promoting activities | $ED_{50}$ = 27.89±7.12 μg/mL (TrkB expressing fibroblasts) | 10 |

| | | | | |
|---|---|---|---|---|
| | | Rho-kinase (ROCK-I and ROCK-II) inhibition | Inhibition percentage at 100μM concentration = 27.4±2.5% (ROCK-I); 29.9±3.6% (ROCK-II) | 11 |
| | | Inhibiting the release of TNF-α, IL-1β and IL-6, anti-inflammatory | NO release inhibition rate, % = 27.64±1.35; Inflammatory factor release, ng/mL = 204.72±8.76 (TNF-α); 67.88±16.97 (IL-1β); 60.59±3.55 (IL-6) | 5 |
| 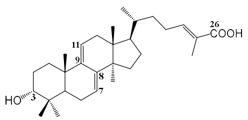 | GA-24 | Inhibiting the release of TNF-α, IL-1β and IL-6, anti-inflammatory | NO release inhibition rate, % = 43.55±0.81; Inflammatory factor release, ng/mL = 157.92±3.11 (TNF-α); 63.31±4.04 (IL-1β); 56.3±4.2 (IL-6) | 5 |
| 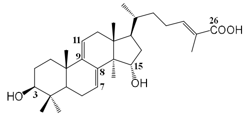 | GA-Jb | Inhibiting the release of TNF-α, IL-1β and IL-6, anti-inflammatory | NO release inhibition rate, % = 38.56±0.58; Inflammatory factor release, ng/mL = 122.32±4.24 (TNF-α); 119.31±4.04 (IL-1β); 42.5±0.79 (IL-6) | 5 |
| | | Activation of human platelet phospholipases C and $A_2$ | - | 12 |
| 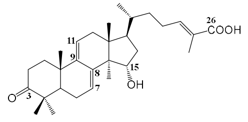 | GA-TR | Inhibitory activities against the HMG-CoA reductase and acyl CoA acyltransferase | $IC_{50}$, μM = 21.7 (HMG-CoA reducta-se); 47.3 (acyl CoA acyltransferase) | 13 |
| | | 5α-reductase inhibition | $IC_{50}$ = 8.5 μM | 14 |
| | | Cytotoxicity against human cervical cancer cell line HeLa | $IC_{50}$ = 58 μM | 6 |
| | | ACE inhibition | $IC_{50}$ = 0.359±0.043 mM | 15 |
| | | The farnesoid X receptor (FXR)-inducing activity | $ED_{50}$ > 14.0 μM | 16 |

| | | | | |
|---|---|---|---|---|
| | | Rho-kinase (ROCK-I and ROCK-II) inhibition | Inhibition percentage at 100μM concentration = 42.4±4.0% (ROCK-I); 40.7±2.6% (ROCK-II) | 11 |
| | | Inhibiting the release of TNF-α, IL-1β and IL-6, anti-inflammatory | NO release inhibition rate, % = 44.37±0.72; Inflammatory factor release, ng/mL = 114.12±1.13 (TNF-α); 143.88±16.16 (IL-1β); 54.04 ± 3.87 (IL-6) | 5 |
| | | Inhibitory activity against fatty acid amide hydrolase (FAAH) | - | 17 |
| 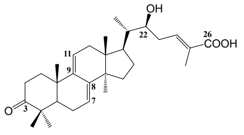 | GA-U1 | Hepatoprotective activity | - | 18 |
| 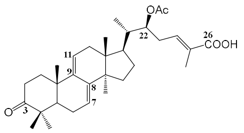 | Ganorbiformin G | Cytotoxicity against cancer cells NCI-H187 (human small-cell lung cancer) and KB (oral human epidermoid carcinoma) | $IC_{50}$, μM = 65 (NCI-H187); 35 (KB) | 19 |
| 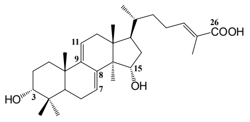 | GA-Ja | Inhibiting the release of TNF-α, IL-1β and IL-6, anti-inflammatory | NO release inhibition rate, % = 38.33±0.22; Inflammatory factor release, ng/mL = 149.72±3.39 (TNF-α); 77.02±4.04 (IL-1β); 55.43±4.19 (IL-6) | 5 |
| | | Activation of human platelet phospholipases C and $A_2$ | - | 12 |

| | | | | |
|---|---|---|---|---|
| 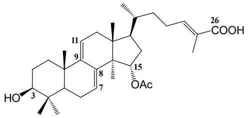 | GA-TN | Rho-kinase (ROCK-I and ROCK-II) inhibition | Inhibition percentage at 100μM concentration = 40.6±1.7% (ROCK-I); 39.2±3.2% (ROCK-II) | 11 |
| | | Activation of human platelet phospholipases C and $A_2$ | - | 12 |
| 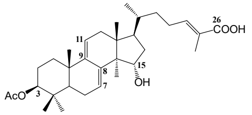 | GA-T-O | Growth inhibitory activity against *Mycobacterium tuberc-ulosis* H37Ra | MIC = 6.25 μg/mL | 20 |
| | | Activation of human platelet phospholipases C and $A_2$ | - | 12 |
| 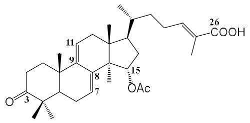 | GA-T-Q | Aldose reductase inhibition | $IC_{50}$ = 41.3±2.5 μM | 21 |
| | | Inhibitory effects on 12-O-tetradecanoylphorbol-13-acetate (TPA)-induced inflammation | $ID_{50}$ = 0.13 mg/ear | 22 |
| | | Brain-derived neurotrophic factor-like neuronal survival-promoting activities | $ED_{50}$ = 5.15±1.36 μg/mL (TrkB expressing fibroblasts) | 10 |
| | | Growth inhibitory activity against *Mycobacterium tuberc-ulosis* H37Ra | MIC = 12.5 μg/mL | 20 |
| | | Rho-kinase (ROCK-I and ROCK-II) inhibition | Inhibition percentage at 100μM concentration = 45.7±2.5% (ROCK-I); 42.4±0.9% (ROCK-II) | 11 |
| | | Inhibiting the release of TNF-α, IL-1β and IL-6, anti-inflammatory | NO release inhibition rate, % = 87.92±0.95; Inflammatory factor release, ng/mL = 129.32±6.22 (TNF-α); 77.6±14.54 (IL-1β); 47.11±0.12 (IL-6) | 5 |

| | | | | |
|---|---|---|---|---|
| 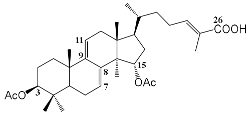 | Ganodermic acid S | Anti-proliferation of tumor cells K562, A549 and HepG2 (liver cancer) | $IC_{50}$, μM = 46.42±3.31 (K562); 68.47±4.08 (A549); 74.42±3.15 (HepG2) | 23 |
| | | Growth inhibitory activity against *Mycobacterium tuberc-ulosis* H37Ra | MIC = 0.391 μg/mL | 20 |
| | | Inhibition of human platelet function | - | 24 |
| 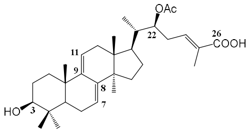 | 3*β*-Hydroxylanosta-22*β*-acetoxy-7,9(11),24-trien-26-oic acid | Growth inhibitory activity against *Mycobacterium tuberc-ulosis* H37Ra | MIC = 6.25 μg/mL | 20 |
| 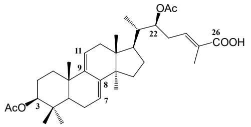 | 3*β*,22*β*-Diacetoxylanosta-7,9(11),24-trien-26-oic acid | Anti-proliferation of tumor cells | - | 23 |
| | | Antitubercular activity against *Mycobacterium tuberculosis* H37Ra | MIC = 12.5 μg/mL | 25 |
| 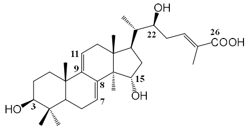 | 3*β*,15*α*,22*β*-Trihydroxylanosta-7,9(11),24-trien-26-oic acid | Inhibiting the release of TNF-α and IL-6, anti-inflammatory | Secretion inhibition rate, % = 64.2±3.4 (TNF-α); 25.1±0.6 (IL-6); $IC_{50}$, μM = 13.1±0.6 (NO production) | 26 |
| | | Anti-proliferation of tumor cells | $IC_{50}$ = 110.92±4.09 μM (K562) | 23 |
| 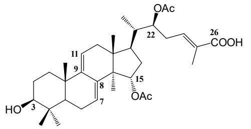 | 3*β*-Hydroxy-15*α*,22*β*-diacetoxylanosta-7,9(11),24-trien-26-oic acid | Growth inhibitory activity against *Mycobacterium tuberc-ulosis* H37Ra | MIC = 33 μg/mL | 20 |

| | | | | |
|---|---|---|---|---|
| 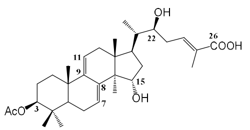 | GA-M2 | Anti-proliferation of tumor cells | $IC_{50}$, μM = 81.33±3.87 (K562); 80.58±3.12 (A549); 131.02±2.09 (HepG2) | 23 |
| | | Inhibiting the release of TNF-α and IL-6, anti-inflammatory | Secretion inhibition rate, % = 62.1±1.6 (TNF-α); 26.3±0.7 (IL-6); $IC_{50}$, μM = 6.9±0.4 (NO production) | 26 |
| 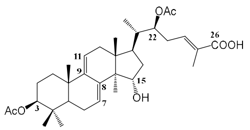 | GA-M1 | Anti-proliferation of tumor cells | $IC_{50}$, μM = 30.59±4.77 (K562); 109.76±3.22 (A549); 115.14±4.12 (HepG2) | 23 |
| | | Inhibiting the release of TNF-α and IL-6, anti-inflammatory | Secretion inhibition rate, % = 94.6±0.5 (TNF-α); 72.4±0.4 (IL-6); $IC_{50}$, μM = 1.5±0.2 (NO production) | 26 |
| 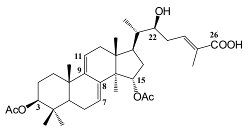 | GA-M3 | Anti-proliferation of tumor cells | $IC_{50}$, μM = 62.72±5.10 (K562); 62.97±2.13 (A549); 62.97±2.80 (HepG2) | 23 |
| 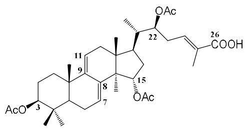 | 3*β*,15*α*,22*β*-Triacetoxylanosta-7,9(11),24-trien-26-oic acid | Cytotoxicity against cancer cells NCI-H187, MCF-7 (human breast cancer), and KB | $IC_{50}$, μM = 13 (NCI-H187); 51 (MCF-7); 13 (KB) | 19 |
| | | Antimalarial activity against *Plasmodium falciparum* K1 | $IC_{50}$ = 4.6 μM | 19 |
| | | Antitubercular activity against *Mycobacterium tuberculosis* H37Ra | MIC = 1.3 μM; 0.781 μg/mL | 19,20 |
| | | Inhibiting the release of TNF-α and IL-6, anti-inflammatory | Secretion inhibition rate, % = 100.0±0.7 (TNF-α); 100.0±0.4 (IL-6); $IC_{50}$, μM = 0.6±0.1 (NO production) | 26 |

| | | | | |
|---|---|---|---|---|
| 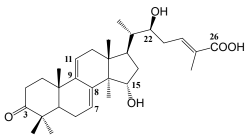 | 3-Oxo-15*α*,22*β*-dihydroxylanosta-7,9(11),24-trien-26-oic acid | Inhibiting the release of TNF-α and IL-6, anti-inflammatory | Secretion inhibition rate, % = 77.5±2.4 (TNF-α); 5.5±0.2 (IL-6); $IC_{50}$, μM = 25.4±2.3 (NO production) | 26 |
| | | Anti-proliferation of tumor cells | $IC_{50}$, μM = 143.25±3.31 (K562); 182.64±2.11 (A549) | 23 |
| 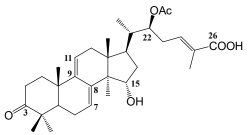 | 3-Oxo-15*α*-hydroxy-22*β*-acetoxylanosta-7,9(11),24- trien-26-oic acid | Cytotoxicity against cancer cell NCI-H187 | $IC_{50}$ = 34 μM | 19 |
| | | Anti-proliferation of tumor cells | $IC_{50}$, μM = 56.52±2.17 (K562); 90.52±5.20 (A549); 153.73±3.31 (HepG2) | 23 |
| | | Inhibiting the release of TNF-α and IL-6, anti-inflammatory | Secretion inhibition rate, % = 72.6±1.2 (TNF-α); 20.4±0.3 (IL-6); $IC_{50}$, μM = 6.5±0.6 (NO production) | 26 |
| | | Hepatoprotective activity | - | 18 |
| 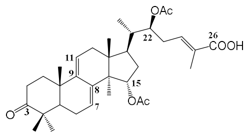 | 3-Oxo-15*α*,22*β*-diacetoxylanosta-7,9(11),24-trien-26-oic acid | Anti-proliferation of tumor cells | $IC_{50}$, μM = 42.07±3.03 (K562); 53.47±4.01 (A549); 80.54±2.11 (HepG2) | 23 |
| | | Inhibiting the release of TNF-α and IL-6, anti-inflammatory | Secretion inhibition rate, % = 98.2±1.3 (TNF-α); 82.8±1.1 (IL-6); $IC_{50}$, μM = 1.3±0.3 (NO production) | 26 |
| | | Inhibitory activity against FAAH | - | 17 |
| 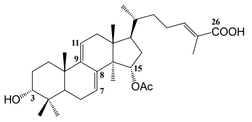 | GA-X | Aldose reductase inhibition | $IC_{50}$ = 32.5±0.3 μM | 21 |
| | | Inhibition of Topo I and Topo IIα activities | - | 27 |
| | | Cytotoxicity against cancer cells HCT-116, HuH-7 (hepatocellular carcinoma), Raji (Burkitt's lymphoma), HL-60 (promyelocyte leukemia) | $IC_{50}$, μg/mL = 38.3 (HCT-116); 20.3 (HuH-7); 39.2 (Raji); 26.5 (HL-60) | 27 |

| | | | | |
|---|---|---|---|---|
| | | Inhibiting the release of TNF-α, IL-1β and IL-6, anti-inflammatory | NO release inhibition rate, % = 89.08±1.1; Inflammatory factor release, ng/mL = 44.12±5.09 (TNF-α); 79.88±8.08 (IL-1β); 42.44±5.67 (IL-6) | 5 |
| 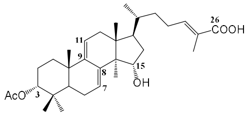  | GA-Mf | Cytotoxicity against cell lines 95-D (human highly metastatic lung tumor) and HeLa | $IC_{50}$, μM = 33.2±0.5 (95-D); 36.4±1.5 (HeLa); | 28 |
| | | Growth inhibition effects on cell lines SW1990 (pancreas), HO-8910PM (ovarian) | $IC_{50}$, μM = 47.1 (SW1990); 34.8 (HO-8910PM); | 29 |
| | | Inhibiting the release of TNF-α, IL-1β and IL-6, anti-inflammatory | NO release inhibition rate, % = 50.29±0.31; Inflammatory factor release, ng/mL = 208.12±1.69 (TNF-α); 227.31±9.69 (IL-1β); 54.04±1.85 (IL-6) | 5 |
| | | Inhibitory activity against FAAH | - | 17 |
| 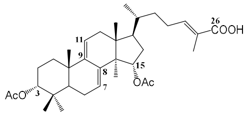  | GA-Me | Cytotoxicity against cancer cell lines HeLa, 95-D, HO-8910PM, SMMC-7721, LoVo (colorectal carcinoma), H1299(lung cancer) | $IC_{50}$, μM = 14.8 (HeLa); 55.1 (95-D); 91.1 (HO-8910PM); 169.6 (SMMC772); 113.6 (LoVo); 83.7 (H1299) | 30 |
| | | Inhibiting the release of TNF-α and IL-6, anti-inflammatory | NO release inhibition rate, % = 76.28±1.77; Inflammatory factor release, ng/mL = 81.24±5.00 (TNF-α); 35.39±4.38 (IL-6) | 31 |
| | | Inducing apoptotic response and arrested the cell cycle of 95-D, HCT-116 and H1299 | - | 30 |
| | | Inhibiting tumor growth and lung metastasis through increasing immune function | - | 32 |

| | | | | |
|---|---|---|---|---|
| | | Inducing T cell apoptosis, restraining T cell activation, and enhancing mediated immunosuppression | - | 33 |
| | | Inhibiting cell migration of 95-D and tumor invasion | - | 34 |
| | | Activation of human platelet phospholipases C and $A_2$ | - | 12 |
| 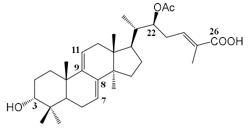 | GA-S | Cytotoxicity against cancer cell lines 95-D, HeLa, HO-8910PM, SW-1990, KB, NCI-H187, K562 and L1210 (mouse leukemia cell) | $IC_{50}$, μM = 38.5±1.8 (95-D); 23.8±0.7 (HeLa); 25.2 (HO-8910PM); 41.4 (SW-1990); 53 (KB); 39 (NCI-H187); 60.20 (K562); 16.03 (L1210); | 19,28,29,35 |
| | | Inhibiting the release of TNF-α and IL-6, anti-inflammatory | NO release inhibition rate, % = 54.03±1.66; Inflammatory factor release, ng/mL = 82.26±1.12 (TNF-α); 27.63±3.67 (IL-6) | 31 |
| 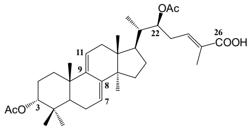 | GA-R | Cytotoxicity against cancer cell lines | $IC_{50}$, μM = 3 1.9±1.1 (95-D); 21.4±0.5 (HeLa); 38.39 (L1210); 40.24 (K562) | 28,35 |
| | | Cytotoxicity on a multidrug resistance tumor cell line (KB-A-1/Dox) and a sensitive tumor cell line (KB-A-1) | - | 36 |
| 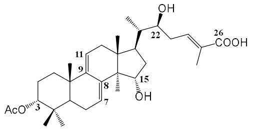 | GA-T2 | Inducing apoptosis in HeLa cells | Approximate $IC_{50}$ = 82 μM | 37 |
| 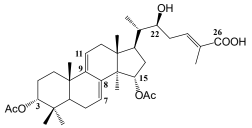 | GA-T1 | Cytotoxicity against cancer cell L1210 | $IC_{50}$ = 21.13±2.54 μM | 38 |
| | | Inducing apoptosis in HeLa cells | Approximate $IC_{50}$ = 31 μM | 37 |
| | | Inhibiting the release of TNF-α and IL-6, anti-inflammatory | NO release inhibition rate, % = 70.72±1.77; Inflammatory factor release, ng/mL = 57.19±0.84 (TNF-α); 19.84±3.19 (IL-6) | 31 |

| | | | | |
|---|---|---|---|---|
| 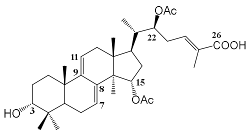 | GA-P | Cytotoxicity against cancer cell lines | $IC_{50}$, μM = 58 (MCF-7); 40 (KB); 26 (NCI-H187); 27.10±3.28 (L1210) | 19,38 |
| | | Antimalarial activity against *Plasmodium falciparum* K1 | $IC_{50}$ = 17 μM | 19 |
| | | Inhibiting the release of TNF-α and IL-6, anti-inflammatory | NO release inhibition rate, % = 54.03±1.10; Inflammatory factor release, ng/mL = 69.4±1.55 (TNF-α); 29.81±6.33 (IL-6) | 31 |
| 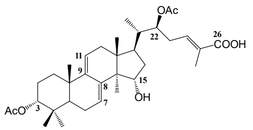 | GA-Mk | Cytotoxicity against cancer cell lines | $IC_{50}$, μM = 29.8±0.3 (HeLa); 30.8±2.4 (95-D); 37.9±1.0 (SW1990); 44.2±1.3 (HO-8910PM); | 28,39 |
| | | Inhibiting the release of TNF-α and IL-6, anti-inflammatory | Secretion inhibition rate, % = 63.8±0.7 (TNF-α); 24.7±0.2 (IL-6); $IC_{50}$, μM = 8.7±0.1 (NO production) | 26 |
| | | Anti-proliferation of HeLa cells | - | 39 |
| | | Inducing apoptosis in HeLa cells | - | 39 |
| 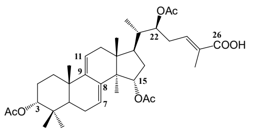 | GA-T | Cytotoxicity against cancer cell lines HeLa, MCF-7, NCI-H187, KB, 95-D, HepG2, L1210 and K562, A375 (melanoma), and Ls174T (colon carcinoma) | $IC_{50}$, μM = 11.0±0.3 (HeLa); 78 (MCF-7); 15 (NCI-H187); 18 (KB); 14.5±1.0 (95-D); 13.1±0.3 (HepG2); 6.55 (L1210); 16.18 (K562); 31.27 (A375); 30.18 (Ls17-4T) | 19,35,40,41 |
| | | Inhibiting the release of TNF-α and IL-6, anti-inflammatory | Secretion inhibition rate, % = 100.0±0.8 (TNF-α); 99.3±0.8 (IL-6); $IC_{50}$, μM = 0.9±0.1 (NO production) | 26 |
| | | Antimalarial activity against *Plasmodium falciparum* K1 | $IC_{50}$ = 5.5 μM | 19 |
| | | Antitubercular activity against *Mycobacterium tuberculosis* H37Ra. | MIC = 10 μM | 19 |
| | | Inhibitory activity against FAAH | - | 17 |

| | | | | |
|---|---|---|---|---|
| | | Inhibiting the growth of solid tumor implanted in athymic mice | - | 42 |
| | | Antimetastatic effect *in vitro* through inhibition of cancer cell invasion | - | 40 |

**Supplemental Table S2. Production of GAs in this and previous studies**

| No. | Structures | Compounds | Species | Sources | Content or titer | Production efficiency [a] | Ref. | This study [b] | | |
|---|---|---|---|---|---|---|---|---|---|---|
| | | | | | | | | Content or titer | Production efficiency | Strain |
| 1 | 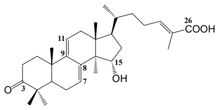 | GA-TR | *G. lucidum* | 10 kg of dried mushroom fruiting bodies | $1.20\times10^{-3}$ mg/g-DW | - | 13 | 136.80±5.51 mg/L (14.71±0.59 mg/g-DW) | 27.36±1.10 mg/L/d (2.94±0.07 mg/g-DW/d) | SC62-AfuSDR-r-CYP512W2-r |
| | | | *G. amboinense* | 1.0 kg of dried mushroom fruiting bodies | $7.30\times10^{-3}$ mg/g-DW | - | 43 | | | |
| | | | *Ganoderma* sp. (strain BCC 16642) | Mycelia harvested from 10 L liquid culture | 1.80 mg/L | $1.62\times10^{-2}$ mg/L/d | 25 | | | |
| | | | *G.mbrekobenum* | 738 g of dried mushroom fruiting bodies | $3.40\times10^{-2}$ mg/g-DW | $2.91\times10^{-4}$ mg/g-DW/d | 44 | | | |
| | | | *G. calidophilum* | 2.5 kg of dried mushroom fruiting bodies | $2.44\times10^{-3}$ mg/g-DW | - | 45 | | | |
| | | | *G. lucidum* | 1 kg of dried mushroom fruiting bodies | $1.90\times10^{-3}$ mg/g-DW | - | 46 | | | |

| | | | | | | | | | | |
|---|---|---|---|---|---|---|---|---|---|---|
| 2 | 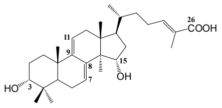 | GA-Ja | *G. lucidum* (strain TP-1) | 56 g of dried mycelia | 5.18×10$^{-2}$ mg/g-DW | 1.73×10$^{-3}$ mg/g-DW/d | 47 | 47.00±4.36 mg/L (5.05±0.47 mg/g-DW) | 9.40±0.87 mg/L/d (1.01±0.09 mg/g-DW/d) | SC3* |
| 3 | 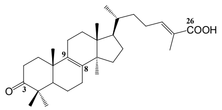 | 3-Oxo-lanosta-8,24-dien-26-oic acid | *G. orbiforme* | 224 g dried mushroom fruiting bodies | 8.04×10$^{-2}$ mg/g-DW | 2.80×10$^{-4}$ mg/g-DW/d | 48 | 1006.97±145.42 mAU·s | 201.39±29.08 mAU·s/d | SC62-CsSDR-AKR1C4-r |
| | | | *Vatica cinerea* | 2.72 kg dried plant materials | 1.13×10$^{-2}$ mg/g-DW | - | 49 | | | |
| 4 | 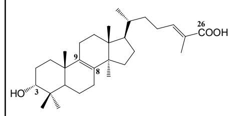 | 3*α*-Hydroxy lanosta-8,24-dien-26-oic acid | - | - | - | - | - | 358.22±32.70 mAU·s | 71.64±6.54 mAU·s/d | SC62-CsSDR-AKR1C4-r |
| 5 | 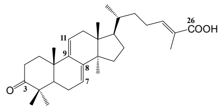 | 3-Oxo-lanosta-7,9(11),24-trien-26-oic acid | *G. orbiforme* | 224 g dried mushroom fruiting bodies | 4.46×10$^{-2}$ mg/g-DW | 1.56×10$^{-4}$ mg/g-DW/d | 48 | 67.86±18.21 mAU·s | 13.57±3.64 mAU·s/d | SC62-CsSDR-AKR1C4-CYP512A2-r |
| | | | *G. lucidum* | 2 kg of dried mushroom fruiting bodies | 2.65×10$^{-2}$ mg/g-DW | - | 50 | | | |
| | | | *G. lucidum* | 6.9 kg of dried mushroom fruiting bodies | 5.80×10$^{-4}$ mg/g-DW | - | 10 | | | |

| | | | | | | | | | | |
|---|---|---|---|---|---|---|---|---|---|---|
| | | | *G. leucocontextum* | 3 kg of dried mushroom fruiting bodies | $1.04\times10^{-2}$ mg/g-DW | $1.04\times10^{-4}$ mg/g-DW/d | 4 | | | |
| 6 | 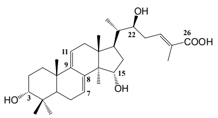 | 3*α*,15*α*,22*β*-Trihydroxylanosta-7,9(11),24-trien-26-oic acid (TLTOA) | - | - | - | - | - | 29.22±3.59 mg/L (3.14±0.39 mg/g-DW) | 5.84±0.72 mg/L/d (0.63±0.08 mg/g-DW/d) | SC1* |
| 7 | 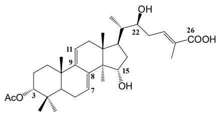 | GA-T2 | *G. lucidum* (strain TP-1) | 56 g of dried mycelia | 0.25 mg/g-DW | $8.33\times10^{-3}$ mg/g-DW/d | 51 | 8.45±0.76 mg/L (0.91±0.08 mg/g-DW) | 1.21±0.11 mg/L/d (0.13±0.01 mg/g-DW/d) | SC1-P$_{GAL1}$BsAT-r |
| 8 | 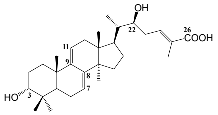 | 3*α*,22*β*-Dihydroxylanosta-7,9(11),24-trien-26-oic acid (DLTOA) | - | - | - | - | - | 5846.67±1205.14 | 825.24±172.16 /d | SC27-GlAT-BsAT-CYP512W6-r-CsSDR-AKR1C4-r |

| | | | | | | | | | | |
|---|---|---|---|---|---|---|---|---|---|---|
| 9 | 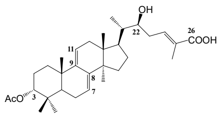 | 3*α*-Acetoxy-22*β*-hydroxylanosta-7,9(11),24-trien-26-oic acid (3*α*-acetoxy-22*β*-HLTOA) | - | - | - | - | - | 10.44±1.72 mg/L (1.12±0/18 mg/g-DW) | 2.09±0.18 mg/L/d (0.22±0.04 mg/g-DW/d) | SC62-BsAT-CYP512W6-r-CsSDR-AKR1C4-CYP512W2-r |
| 10 | 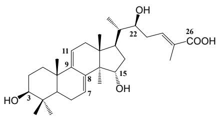 | 3*β*,15*α*,22*β*-Trihydroxylanosta-7,9(11),24-trien-26-oic acid (3*β*-TLTOA) | *G. amboinense* | 1.0 kg of dried fruiting bodies | $7.20\times10^{-3}$ mg/g-DW | - | 43 | 21.45±2.03 mg/L (2.31±0.22 mg/g-DW) | 4.29±0.41 mg/L/d (0.46±0.04 mg/g-DW/d) | SC62-CYP512W6-r-CYP512W2-r |
| | | | *G. lucidum* (strain TP-1) | 56 g of dried mycelia | $3.21\times10^{-2}$ mg/g-DW | $1.07\times10^{-3}$ mg/g-DW/d | 52 | | | |
| | | | *G. cochlear* | 68 kg of dried fruiting bodies | $3.38\times10^{-4}$ mg/g-DW | - | 53 | | | |
| 11 | 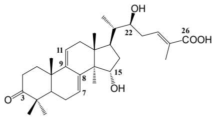 | 3-Oxo-15*α*,22*β*-dihydroxylanosta-7,9(11),24-trien-26-oic acid | - | - | - | - | - | 115.05±21.11mAU·s | 23.01±4.22 mAU·s/d | SC62-CYP512W6-r-CsSDR-AKR1C4-CYP512W2-r, |
| 12 | 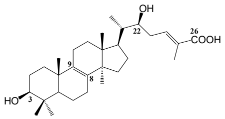 | 3*β*,22*β*-Dihydroxylanosta-8,24-dien-26-oic acid (DLDOA) | *Ganoderma* sp. (strain BCC 16642) | Mycelia harvested from 10 L liquid culture | 0.11 mg/L | $9.91\times10^{-4}$ mg/L/d | 25 | 17.84±0.27 mg/L (1.92±0.03 mg/g-DW) | 3.57±0.05 mg/L/d (0.38±0.01 mg/g-DW/d) | SC62-CYP512W6-r |

| | | | | | | | | | | |
|---|---|---|---|---|---|---|---|---|---|---|
| 13 | 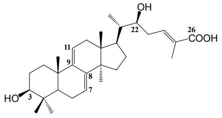 | 3*β*,22*β*-Dihydroxylanosta-7,9(11),24-trien-26-oic acid | - | - | - | - | - | 113.38±23.42 mAU·s | 22.68±4.68 mAU·s/d | SC62-CYP512W6-r-CYP512W2-r |
| 14 | 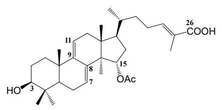 | GA-TN | - | - | - | - | - | 73.45±16.34 mg/L (7.90±1.76 mg/g-DW) | 14.69±3.27 mg/L/d (1.58±0.35 mg/g-DW/d) | SC62-GlAT-r-CYP512W2-r |
| 15 | 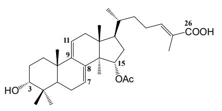 | GA-X | *G. orbiforme* (strain BCC 22324) | Mycelia harvested from 7 L liquid culture | 0.14 mg/L | $5.10\times10^{-3}$ mg/L/d | 19 | 1.43±0.05 mg/L (0.15±0.01 mg/g-DW) | 0.20±0.01 mg/L/d (0.02 mg/g--DW/d) | SC62-GlAT-r-CsSDR-AKR1C4-CYP512W2-r |
| | | | *Ganoderma* sp. (strain BCC 16642) | Mycelia harvested from 10 L liquid culture | 0.29 mg/L | $2.61\times10^{-3}$ mg/L/d | 25 | | | |
| | | | *G. orbiforme* (strain BCC 22325) | Mycelia harvested from 15 L liquid culture | 0.73 mg/L | $2.16\times10^{-2}$ mg/L/d | 20 | | | |

| | | | | | | | | | | |
|---|---|---|---|---|---|---|---|---|---|---|
| 16 | 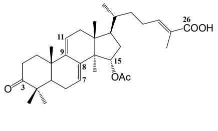 | GA-T-Q | *G. lucidum* | 373 g of dried mushroom fruiting bodies | $5.42\times10^{-2}$ mg/g-DW | - | 54 | 265.53±55.89 mAU·s | 53.11±11.18 mAU·s/d | SC62-GlAT-r-CsSDR-AKR1C4-CYP512W2-r |
| | | | *G. lucidum* | 2 kg of dried mushroom fruiting bodies | $1.25\times10^{-2}$ mg/g-DW | - | 6 | | | |
| | | | *G. hainanense* | 2.5 kg of dried mushroom fruiting bodies | $4.00\times10^{-3}$ mg/g-DW | - | 55 | | | |
| | | | *G. lucidum* | 6.9 kg of dried mushroom fruiting bodies | $2.90\times10^{-3}$ mg/g-DW | - | 10 | | | |
| | | | *G. orbiforme* (strain BCC 22324) | Mycelia harvested from 20 L liquid culture | 0.10 mg/L | $3.57\times10^{-3}$ mg/L/d | 19 | | | |
| | | | *G.orbiforme* (strain BCC 22325) | Mycelia harvested from 15 L liquid culture | 3.00 mg/L | $8.80\times10^{-2}$ mg/L/d | 20 | | | |
| 18 | 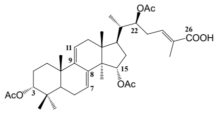 | GA-T | *G. lucidum* (strain TP-1) | 56 g of dried mycelia | 0.25 mg/g-DW | $8.33\times10^{-3}$ mg/g-DW/d | 51 | 3003.33±144.97 | 429.05±20.71 /d | SC27-$P_{ADH2}$BsAT-CYP512W6-$P_{GAL1}$GlAT- |

| | | | | | | | | | | |
|---|---|---|---|---|---|---|---|---|---|---|
| | | | *G. lucidum* (strain CGMCC 5.0644) | Liquid shaking and static culture (nitrogen-limiting conditions) | 206.40 mg/L | 6.07 mg/L/d | 56 | | | r-CsSDR-AKR1C4-r |
| | | | *G. orbiforme* (strain BCC 22324) | Mycelia harvested from 20 L liquid culture | 2.45 mg/L | $8.75\times10^{-2}$ mg/L/d | 19 | | | |
| | | | *Ganoderma* sp. (strain BCC 16642) | Mycelia harvested from 10 L liquid culture | 4.30 mg/L | $3.87\times10^{-2}$ mg/L/d | 25 | | | |
| | | | *Ganoderma* sp. (strain BCC 22325) | Mycelia harvested from 15 L liquid culture | 13.47 mg/L | $3.96\times10^{-2}$ mg/L/d | 20 | | | |
| | | | *G. lucidum* (strain CCGMC 5.616) | 200 g of dried mycelia | 0.50 mg/g-DW | - | 57 | | | |
| | | | *G. lucidum* (strain CCGMC 5.616) | Liquid shaking and static culture | 2.056 mg/g-DW | $7.09\times10^{-2}$ mg/g-DW/d | 58 | | | |

| | | | | | | | | | | |
|---|---|---|---|---|---|---|---|---|---|---|
| | | | *G. lucidum* (strain CCGMC 5.616) | Liquid shaking and static culture (nitrogen-limiting conditions) | 11.76±1.82 mg/g-DW | 0.44 mg/g-DW/d | 59 | | | |
| | | | *G. lucidum* (strain CCGMC 5.616) | Liquid shaking and static culture (10 mM $Ca^{2+}$) | 10.90±2.07 mg/g-DW | 0.40 mg/g-DW/d | 60 | | | |
| | | | *G. lucidum* (strain CCGMC 5.616) | Liquid shaking and static culture (100 mM $Mn^{2+}$) | 13.50±3.03 mg/g-DW | 0.50 mg/g-DW/d | 61 | | | |
| | | | *G. lucidum* (strain CCGMC 5.616) | Liquid shaking and static culture (10 mM $Na^{+}$) | 16.17±2.12 mg/g-DW | 0.60 mg/g-DW/d | 62 | | | |
| | | | *G. lucidum* (strain CCGMC 5.616) | Liquid shaking and static culture (overexpression of *lss*) | 0.698±0.082 mg/g-DW | $3.03\times10^{-2}$ mg/g-DW/d | 63 | | | |

| | | | | | | | | | | |
|---|---|---|---|---|---|---|---|---|---|---|
| | | | *G. lucidum* (strain CCGMC 5.616) | Liquid shaking and static culture (overexpression of *fps*) | 0.41±0.02 mg/g-DW | 1.78×10$^{-2}$ mg/g-DW/d | 64 | | | |
| | | | *G. lucidum* (strain CCGMC 5.616) | Shake flask fermentation (overexpression of *VHb*) | 0.346±0.021 mg/g-DW | 1.73×10$^{-2}$ mg/g-DW/d | 65 | | | |
| | | | *G. lucidum* (strain CCGMC 5.616) | Shake flask fermentation (overexpression of *sqs*) | 0.40 mg/g-DW | 0.02 mg/g-DW/d | 66 | | | |
| | | | *G. lucidum* (strain CCGMC 5.616) | Shake flask fermentation (overexpression of *hmgr, se*) | 0.904±0.075 mg/g-DW | 4.52×10$^{-2}$ mg/g-DW/d | 67 | | | |
| 19 | 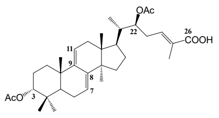 | GA-R | *G. orbiforme* (strain BCC 22324) | Mycelia harvested from 7 L liquid culture | 0.14 mg/L | 5.10×10$^{-3}$ mg/L/d | 19 | 8.08±0.61 mg/L (0.87±0.09 mg/g-DW) | 1.15±0.09 mg/L/d (0.12±0.01 mg/g- | SC27-$P_{ADH2}$BsAT-CYP512W6-$P_{GAL1}$GlAT- |

| | | | | | | | | | | |
|---|---|---|---|---|---|---|---|---|---|---|
| | | | *Ganoderma* sp. (strain BCC 16642) | Mycelia harvested from 10 L liquid culture | 2.70 mg/L | $2.43\times10^{-2}$ mg/L/d | 25 | | DW/d) | r-CsSDR-AKR1C4-r |
| | | | *Ganoderma* sp. (strain BCC 22325) | Mycelia harvested from 15 L liquid culture | 0.67 mg/L | $1.96\times10^{-2}$ mg/L/d | 20 | | | |
| | | | *G. lucidum* (strain CGMCC 5.0644) | Liquid shaking and static culture (nitrogen-limiting conditions) | 50.60 mg/L | 1.27 mg/L/d | 56 | | | |
| 22 | 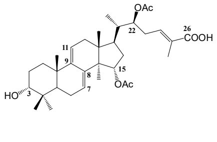 | GA-P | *G. orbiforme* (strain BCC 22324) | Mycelia harvested from 20 L liquid culture | 1.50 mg/L | $5.36\times10^{-2}$ mg/L/d | 19 | 3531.77±477.02 mAU·s | 504.54±59.63 mAU·s/d | SC27-$P_{ADH2}$GlAT-CYP512W6-$P_{GAL1}$BsAT-r-CsSDR-AKR1C4-r |
| | | | *Ganoderma* sp. (strain BCC 22325) | Mycelia harvested from 15 L liquid culture | 8.73 mg/L | $2.57\times10^{-2}$ mg/L/d | 20 | | | |

| | | | | | | | | | | |
|---|---|---|---|---|---|---|---|---|---|---|
| | | | *G. lucidum* (strain CGMCC 5.0644) | Liquid shaking and static culture (nitrogen-limiting conditions) | 9.67 mg/L | 0.24 mg/L/d | 56 | | | |
| 23 | 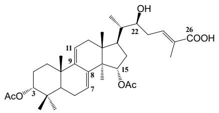 | GA-T1 | - | - | - | - | - | 116.00±28.47 | 16.57±4.07 /d | SC27-$P_{ADH2}$GlAT-CYP512W6-$P_{GAL1}$BsAT-r-CsSDR-AKR1C4-r |
| 24 | 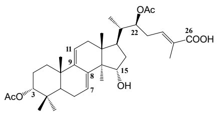 | GA-Mk | *G. lucidum* (strain TP-1) | 56 g of dried mycelia | 0.11 mg/g-DW | $3.81\times10^{-3}$ mg/g-DW/d | 47 | 118.67±24.73 | 16.95±3.53 /d | SC27-$P_{ADH2}$BsAT-CYP512W6-$P_{GAL1}$GlAT-r-CsSDR-AKR1C4-r |
| | | | *G. lucidum* | 300 g of dried mycelia | 0.05 mg/g-DW | - | 28 | | | |
| | | | *G. lucidum* (strain CCGMC 5.616) | Liquid shaking and static culture | 0.426 mg/g-DW | $1.70\times10^{-2}$ mg/g-DW/d | 58 | | | |

| | | | | | | | | | | |
|---|---|---|---|---|---|---|---|---|---|---|
| | | | *G. lucidum* (strain CCGMC 5.616) | Liquid shaking and static culture (nitrogen-limiting conditions) | 2.16±0.19 mg/g-DW | 8.00×$10^{-2}$ mg/g-DW/d | 59 | | | |
| | | | *G. lucidum* (strain CCGMC 5.616) | Liquid shaking and static culture (10 mM $Ca^{2+}$) | 2.55±0.45 mg/g-DW | 9.44×$10^{-2}$ mg/g-DW/d | 60 | | | |
| | | | *G. lucidum* (strain CCGMC 5.616) | Liquid shaking and static culture (10 mM $Mn^{2+}$) | 12.01±0.04 mg/g-DW | 0.45 mg/g-DW/d | 61 | | | |
| | | | *G. lucidum* (strain CCGMC 5.616) | Liquid shaking and static culture (100 mM $Na^{+}$) | 15.76±0.52 mg/g-DW | 0.58 mg/g-DW/d | 62 | | | |
| | | | *G. lucidum* (strain CCGMC 5.616) | Liquid shaking and static culture (overexpression of *lss*) | 0.243±0.035 mg/g-DW | 1.06×$10^{-2}$ mg/g-DW/d | 63 | | | |

| | | | | | | | | | | |
|---|---|---|---|---|---|---|---|---|---|---|
| | | | *G. lucidum* (strain CCGMC 5.616) | Shake flask fermentation (overexpression of *VHb*) | 1.915±0.131 mg/g-DW | $9.58\times10^{-2}$ mg/g-DW/d | 65 | | | |
| | | | *G. lucidum* (strain CCGMC 5.616) | Shake flask fermentation (overexpression of *sqs*) | 0.16 mg/g-DW | $8.00\times10^{-3}$ mg/g-DW/d | 66 | | | |
| | | | *G. lucidum* (strain CCGMC 5.616) | Shake flask fermentation (overexpression of *hmgr, se*) | 0.062±0.005 mg/g-DW | $3.65\times10^{-3}$ mg/g-DW/d | 67 | | | |
| 25 | 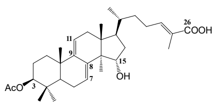 | GA-T-O | *G. casuarinicola* (strain BCC 78460) | 953 g of dried mushroom fruiting bodies | $6.09\times10^{-3}$ mg/g-DW | $6.27\times10^{-5}$ mg/g-DW/d | 68 | 81.00±8.34 mAU·s | 16.20±1.67 mAU·s/d | SC62-BsAT-r-CYP512W2-r |
| | | | *G. austral* (strain TBRC-BCC 22314) | 100 g of dried mycelia | 0.68 mg/g-DW | $6.54\times10^{-3}$ mg/g-DW/d | 69 | | | |
| | | | *Ganoderma* sp. (strain BCC 60695) | Mycelia harvested from 10 L liquid culture | 8.80 mg/L | $2.10\times10^{-2}$ mg/L/d | 20 | | | |

| | | | | | | | | | | |
|---|---|---|---|---|---|---|---|---|---|---|
| | | | *G. australe* (strain BCC 22314) | Mycelia harvested from 20.75 L liquid culture | 3.76 mg/L | 3.42×10$^{-2}$ mg/L/d | 20 | | | |
| | | | *G. australe* (strain BCC 22314) | Mycelia harvested from 30 L liquid culture | 17.03 mg/L | 0.16 mg/L/d | 70 | | | |
| 26 | 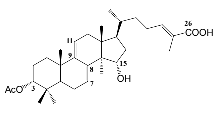 | GA-Mf | *G. lucidum* (strain CCGMC 5.616) | 300 g of dried mycelia | 0.05 mg/g-DW | - | 28 | 12.16±1.40 mg/L (2.43±0.15 mg/g-DW) | 2.43±0.28 mg/L/d (0.49±0.03 mg/g-DW/d) | SC62-BsAT-r-CsSDR-AKR1C4-CYP512W2-r |
| 27 | 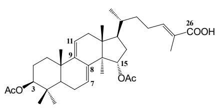 | Ganodermic acid S (GMA-S) | *G. lucidum* (strains TP-1 and AT-4) | 146 g of dried mycelia (56 g TP-1, 90 g AT-4) | 1.03 mg/g-DW | 3.42×10$^{-2}$ mg/g-DW/d | 71 | 780.00±86.41 | 111.43±12.34 /d | SC27-$P_{ADH2}$BsAT-CYP512W6-$P_{GAL1}$GlAT-r-CsSDR-AKR1C4-r |
| | | | *G. austral* (strain TBRC-BCC 22314) | 100 g dried mycelia | 0.87 mg/g-DW | 8.37×10$^{-3}$ mg/g-DW/d | 69 | | | |

| | | | | | | | | | | |
|---|---|---|---|---|---|---|---|---|---|---|
| | | | *G. orbiforme* (strain BCC 22325) | Mycelia harvested from 15 L liquid culture | 0.39 mg/L | $1.16\times10^{-2}$ mg/L/d | 20 | | | |
| | | | *Ganoderma* sp. (strain BCC 60695) | Mycelia harvested from 10 L liquid culture | 13.10 mg/L | $3.12\times10^{-2}$ mg/L/d | 20 | | | |
| | | | *G. australe* (strain BCC 22314) | Mycelia harvested from 20.75 L liquid culture | 6.80 mg/L | $6.18\times10^{-2}$ mg/L/d | 20 | | | |
| | | | *G. australe* (strain BCC 22314) | Mycelia harvested from 30 L liquid culture | 26.40 mg/L | 0.25 mg/L/d | 70 | | | |
| 28 | 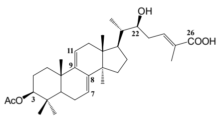 | 3*β*-Acetoxy-22*β*-hydroxylanosta-7,9(11),24-trien-26-oic acid | - | - | - | - | - | 211.87±17.19 mAU·s | 30.27±2.46 mAU·s/d | SC1-$P_{GAL1}$BsAT-r |

| | | | | | | | | | | |
|---|---|---|---|---|---|---|---|---|---|---|
| 29 | 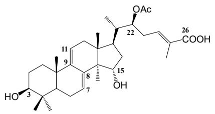 | 3*β*, 15*α*-Dihydroxy-22*β*-acetoxylanosta-7,9(11),24-trien-26-oic acid | *G. lucidum* (strain TP-1) | 56 g of dried mycelia | $6.07×10^{-2}$ mg/g-DW | $2.02×10^{-3}$ mg/g-DW/d | 52 | 206.08±36.65 mAU·s | 29.44±5.24 mAU·s/d | SC27-$P_{ADH2}$BsAT-CYP512W6-$P_{GAL1}$GlAT-r |
| | 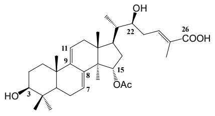 | 3*β*, 22*β*-Dihydroxy-15*α*-acetoxylanosta-7,9(11),24-trien-26-oic acid | - | - | - | - | - | | | |
| 30 | 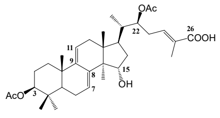 | GA-M1 | - | - | - | - | - | 460.23±43.86 mAU·s | 65.75±6.27 mAU·s/d | SC27-$P_{ADH2}$GlAT-CYP512W6-$P_{GAL1}$BsAT-r |
| | 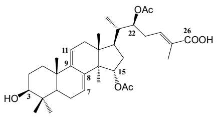 | 3*β*-Hydroxy-15*α*,22*β*-diacetoxylanosta-7,9(11),24-trien-26-oic acid | *G. orbiforme* (strain BCC 22324) | Mycelia harvested from 20 L liquid culture | 0.25 mg/L | $8.92×10^{-3}$ mg/L/d | 19 | | | |

| | | | | | | | | | | |
|---|---|---|---|---|---|---|---|---|---|---|
| | | | *Ganoderma* sp. (strain BCC 16642) | Mycelia harvested from 10 L liquid culture | 2.6 mg/L | $2.34\times10^{-2}$ mg/L/d | 25 | | | |
| | | | *Ganoderma* sp. (strain BCC 22325) | Mycelia harvested from 15 L liquid culture | 0.33 mg/L | $9.80\times10^{-3}$ mg/L/d | 20 | | | |
| | | | *Ganoderma* sp. (strain BCC 60695) | Mycelia harvested from 10 L liquid culture | 0.5 mg/L | $1.19\times10^{-2}$ mg/L/d | 20 | | | |
| 31 | 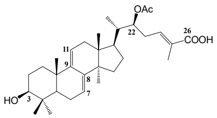 | 3*β*-Hydroxy-22*β*-acetoxylanosta-7,9(11),24-trien-26-oic acid | *Ganoderma* sp. (strain BCC 16642) | Mycelia harvested from 10 L liquid culture | 0.18 mg/L | $1.62\times10^{-3}$ mg/L/d | 25 | 167.31±17.89 mAU·s | 23.90±2.56 mAU·s/d | SC27-$P_{ADH2}$GlAT-CYP512W6-$P_{GAL1}$BsAT-r |
| | | | *Ganoderma* sp. (strain BCC 60695) | Mycelia harvested from 10 L liquid culture | 1.80 mg/L | $4.29\times10^{-2}$ mg/L/d | 20 | | | |

| | | | | | | | | | | |
|---|---|---|---|---|---|---|---|---|---|---|
| 32 | 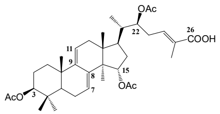 | 3*β*,15*α*,22*β*-Triacetoxylanosta-7,9(11),24-trien-26-oic acid | *G. lucidum* (strain TP-1) | 56 g of dried mycelia | $6.25\times10^{-2}$ mg/g-DW | $2.08\times10^{-3}$ mg/g-DW/d | 51 | 40.96±4.91 mAU·s | 5.85±0.70 mAU·s/d | SC27-$P_{ADH2}$BsAT-CYP512W6-$P_{GAL1}$GlAT-r |
| | | | *G. amboinense* | 1.0 kg of dried fruiting bodies | $7.70\times10^{-3}$ mg/g-DW | - | 43 | | | |
| | | | *G. orbiforme* (strain BCC 22324) | Mycelia harvested from 20 L liquid culture | 0.70 mg/L | $2.50\times10^{-2}$ mg/L/d | 19 | | | |
| | | | *Ganoderma* sp. (strain BCC 16642) | Mycelia harvested from 10 L liquid culture | 2.20 mg/L | $1.98\times10^{-2}$ mg/L/d | 25 | | | |
| | | | *Ganoderma* sp. (strain BCC 22325) | Mycelia harvested from 15 L liquid culture | 2.00 mg/L | $5.88\times10^{-2}$ mg/L/d | 20 | | | |
| | | | *Ganoderma* sp. (strain BCC 60695) | Mycelia harvested from 10 L liquid culture | 1.00 mg/L | $2.38\times10^{-2}$ mg/L/d | 20 | | | |

| | | | | | | | | | | |
|---|---|---|---|---|---|---|---|---|---|---|
| 33 | 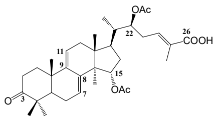 | 3-Oxo-15$\alpha$,22$\beta$-diacetoxylanosta-7,9(11),24-trien-26-oic acid | *G. orbiforme* (strain BCC 22324) | Mycelia harvested from 20 L liquid culture | 0.40 mg/L | $1.43\times10^{-2}$ mg/L/d | 19 | 6846.67±861.99 | 978.09±123.14 /d | SC27-$P_{ADH2}$BsAT-CYP512W6-$P_{GAL1}$GlAT-r-CsSDR-AKR1C4-r |
| | | | *Ganoderma* sp. (strain BCC 16642) | Mycelia harvested from 10 L liquid culture | 5.50 mg/L | $4.95\times10^{-2}$ mg/L/d | 25 | | | |
| | | | *Ganoderma* sp. (strain BCC 22325) | Mycelia harvested from 15 L liquid culture | 3.60 mg/L | $1.06\times10^{-2}$ m/L/d | 20 | | | |
| | | | *Ganoderma* sp. (strain BCC 60695) | Mycelia harvested from 10 L liquid culture | 1.10 mg/L | $2.50\times10^{-2}$ mg/L/d | 20 | | | |
| 34 | 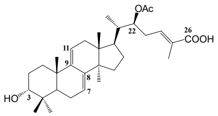 | GA-S | *G. orbiforme* (strain BCC 22324) | Mycelia harvested from 20 L liquid culture | 0.60 mg/L | $2.14\times10^{-2}$ mg/L/d | 19 | 23760.00±4438.38 | 3394.29±634.03 /d | SC27-$P_{ADH2}$GlAT-CYP512W6-$P_{GAL1}$BsAT-r-CsSDR-AKR1C4-r |
| | | | *Ganoderma* sp. (strain BCC 16642) | Mycelia harvested from 10 L liquid culture | 1.40 mg/L | $1.26\times10^{-2}$ mg/L/d | 25 | | | |

| | | | | | | | | | | |
|---|---|---|---|---|---|---|---|---|---|---|
| | | | *Ganoderma* sp. (strain BCC 22325) | Mycelia harvested from 15 L liquid culture | 1.27 mg/L | $3.73\times10^{-2}$ mg/L/d | 20 | | | |
| | | | *G. lucidum* | 300 g of dried mycelia | 0.14 mg/g-DW | - | 28 | | | |
| | | | *G. lucidum* (strain CCGMC 5.616) | Liquid shaking and static culture | 0.558 mg/g DW | $2.23\times10^{-2}$ mg/g-DW/d | 58 | | | |
| | | | *Ganoderma* sp. | Liquid shaking and static culture | 0.89 mg/g | $2.54\times10^{-2}$ mg/g-DW/d | 72 | | | |
| | | | *G. lucidum* (strain CGMCC 5.0644) | Liquid shaking and static culture (nitrogen-limiting conditions) | 88.55 mg/L | 2.21 mg/L/d | 56 | | | |
| | | | *G. lucidum* (strain CCGMC 5.616) | Liquid shaking and static culture (nitrogen-limiting conditions) | 31.09±2.67 mg/g-DW | 1.15 mg/g-DW/d | 59 | | | |

| | | | | | | | | | | | |
|---|---|---|---|---|---|---|---|---|---|---|---|
| | | | *G. lucidum* (strain CCGMC 5.616) | Liquid shaking and static culture (10 mM $Ca^{2+}$) | 2.79±0.87 mg/g-DW | 0.12 mg/g-DW/d | 60 | | | | |
| | | | *G. lucidum* (strain CCGMC 5.616) | Liquid shaking and static culture (1 mM $Mn^{2+}$) | 4.68±0.17 mg/g-DW | 0.17 mg/g-DW/d | 61 | | | | |
| | | | *G. lucidum* (strain CCGMC 5.616) | Liquid shaking and static culture (100 mM $Na^{+}$) | 4.93±0.16 mg/g-DW | 0.18 mg/g-DW/d | 62 | | | | |
| | | | *G. lucidum* (strain CCGMC 5.616) | Liquid shaking and static culture (overexpression of *lss*) | 0.289±0.014 mg/g-DW | $1.26\times10^{-2}$ mg/g-DW/d | 63 | | | | |
| | | | *G. lucidum* (strain CCGMC 5.616) | Liquid shaking and static culture (overexpression of *fps*) | 0.21±0.05 mg/g-DW | $9.13\times10^{-3}$ mg/g-DW/d | 64 | | | | |

| | | | | | | | | | | |
|---|---|---|---|---|---|---|---|---|---|---|
| | | | *G. lucidum* (strain CCGMC 5.616) | Shake flask fermentation (overexpression of *VHb*) | 0.191±0.018 mg/g-DW | $9.55\times10^{-3}$ mg/g-DW/d | 65 | | | |
| | | | *G. lucidum* (strain CCGMC 5.616) | Shake flask fermentation (overexpression of *sqs*) | 0.53 mg/g-DW | $2.30\times10^{-2}$ mg/g-DW/d | 66 | | | |
| | | | *G. lucidum* (strain CCGMC 5.616) | Shake flask fermentation (overexpression of *hmgr, se*) | 0.359±0.054 mg/g-DW | $2.11\times10^{-2}$ mg/g-DW/d | 67 | | | |

[a] For *G. lucidum* mycelia cultivation, about 8-16 days preculture in liquid medium is included in the whole fermentation process.

[b] For GAs which have authentic standards, the production levels were characterized by yield (mg/L). For GAs which lack authentic standards, the production levels were characterized by peak area (mAU·s). For GAs which lack authentic standards and exhibit similar LC patterns to other intermediate, the production levels were characterized by mass ion abundance (without unit). After 120 or 168 h fermentation, yeast strains produced 1.43±0.05-136.80±5.51 mg/L of GAs, with $OD_{600}$ at about 30. According to the conversion method as published previously[73], yeast strains produced 0.15±0.01-14.71±0.59 mg/g-DW of GAs. The production efficiency of GAs by yeast strains ranged from 0.02 to 2.94 ± 0.07 mg/g-DW/d.

## Supplementary materials and methods

### Chemicals and enzymes.

GA-Mk, GA-T, GA-S, and GA-R were obtained by extraction and purification from *G. lucidum* fermentation cultures. **1** was obtained by extraction and purification of the reaction products following the incubation of *G. lucidum* crude protein lysates with GA-Jb. **6** and **7** were obtained by extraction and purification of alkaline hydrolysis products of GA-T, whereas **8** and **9** were obtained by extraction and purification of alkaline hydrolysis products of GA-R. GA-Jb, GA-Y, HLDOA, **2**, **10**, **12**, **14**, and **26** were obtained by extraction and purification of yeast fermentation cultures. GA-X was purchased from ChemFaces (Wuhan, China). GA-P and GA-24 were kindly provided by Dr. Na Feng (Institute of Edible Fungi, Shanghai Academy of Agricultural Sciences). All endonucleases used in this study were purchased from New England Biolabs (Ipswich, MA, USA).

### *In vitro* reactions with crude protein lysates of *G. lucidum*

Frozen *G. lucidum* mycelium samples were ground into fine powders under liquid nitrogen protection using a freezing mortar and pestle. The powders were dissolved in 0.1 M Tris-HCl (pH=8), and centrifuged at 12,000 *g*, 4 °C for 10 min. The supernatant was collected and sequentially filtered using filter paper, followed by 0.45 μm and 0.22 μm filter membrane with the aid of a vacuum pump (SHZ-III, Shengye, Shanghai, China). The collected fluids were the crude protein lysates, which were stored at -80 °C in a buffer containing 20% glycerol for subsequent use.

The *in vitro* reaction was conducted in a total volume of 1 mL of 0.1 M Tris-HCl buffer, containing an appropriate amount of crude protein lysates and 10 μg/mL GA-Jb. For the control experiment, an equivalent amount of crude protein lysate was heated at 80 °C for 10 min before incubation with

GA-Jb. After incubation at 30 °C and 120 rpm for 3 h, the reaction product was extracted twice using 800 μl of ethyl acetate. After centrifugation, the supernatant was collected, evaporated and redissolved in methanol for HPLC analysis.

**cDNA synthesis, RNA sequencing and data analysis**

Total RNA was extracted from frozen samples by using Trizol (Sigma-Aldrich, MA, USA), and cDNA was prepared from the extracted mRNA with the FastKing RT Kit (TIANGEN, Beijing, China) following the manufacturer's instructions. RNA concentration and quality were determined using a NanoDrop2000™ spectrophotometer (Thermo Fisher Scientific, MA, USA) and agarose gel electrophoresis. For RNA sequencing, frozen *G. lucidum* mycelia samples were sent to Tianjin Novogene Bioinformatic Technology Co., Ltd for RNA isolation, library preparation, and transcriptome sequencing [74].

Differential expression analysis was performed by taking the logarithm of the ratio of gene expression levels in the blue light exposure group to those in the dark cultivation group (i.e., $Log_2$FoldChange). Co-expression analysis was performed using R (version 4.0.2) to calculate Pearson's correlation coefficient (PCC) for each gene to the bait genes (*cyp5150l8* and *cyp512w2*). A list of strongly co-expressed genes (PCC > 0.6) was obtained and arranged in descending order of PCC (Supplementary Table S4). FPKM values of genes were $log_2$-normalized for heatmapping using the NovoMagic platform (https://magic.novogene.com).

**Cloning of candidate genes**

Primers used in this study were synthesized by GENEWIZ Biotechnology Co.,Ltd. (Suzhou, China) and listed in Supplementary Table S6. Candidate genes were amplified using Phanta Max Super-Fidelity DNA Polymerase (Novoprotein, Nanjing, China) with *G. lucidum* cDNA as the template. PCR products were purified by the TIANgel Midi Purification Kit (TIANGEN) and inserted into *Pme*I linearized pRS426-HXT7p-FBA1t-G418r using the Trelief® SoSoo Cloning Kit (Tsingke, Beijing, China). Plasmids were isolated using the TIANprep Mini Plasmid Kit (TIANGEN), and sequencing was confirmed by Sanger sequencing (GENEWIZ). Heterologous genes (Supplementary Table S5) were synthesized by GENEWIZ or SynbioB (Tianjing, China). Detailed procedures for plasmids and strains construction were summarized in Supplementary Table S7.

**Whole-cell catalysis**

Different yeast colonies were inoculated into 3 mL of YPD medium and incubated for one day. Subsequently, the cultures were transferred into 3 mL of YPD24 (containing 10 g/L of yeast extract, 20 g/L of beef peptone, 20 g/L of glucose, and 40 g/L glycerol) [75] medium at an initial $OD_{600}$ of 0.05, with the addition of GA-X (dissolved in methanol) at a final concentration of 400 μM, and fermented for 5 days in round-bottomed test tubes. Three mL of the fermentation broth was centrifuged at 6,000 *g* for 5 min to separate the cells from the supernatant. Three mL of distilled water and 2.4 mL of ethyl acetate were added to the cell precipitates, and 2.4 mL of ethyl acetate was added to the supernatant for metabolites extraction.

To screen the C3 reductase candidates, the BJ5464 derived strains were cultured in YPD24 containing 300 mg/L G418 for 72 h, after which GA-TR was added to a final concentration of 50

μM. After another 48 h of incubation, metabolites were extracted for HPLC detection. To test the catalytic efficiency of GlAT mutants, the BJ5464 derived strains were cultured under the same condition, and different GA substrates were individually added to a final concentration of 10 μM. After another 3 h of incubation, metabolites were extracted for HPLC detection.

**Liter-scale fermentation, extraction and purification of compounds**

To obtain GA-MK, GA-T, GA-R, and GA-S, 20 g of dried *G. lucidum* mycelium powders were extracted with 0.8 L of ethyl acetate. To obtain **1**, 126 mg of *G. lucidum* crude protein lysates were incubated with 5.2 mg of GA-Jb in 50 mL Tris-HCl for 3 h and extracted with 50 mL ethyl acetate. To obtain **6**, **7**, **8**, and **9**, 12 mg of GA-T and 6 mg of GA-R were subjected to alkaline hydrolysis and extracted with equal volumes of ethyl acetate. To obtain **2**, **10**, **12**, **14**, and **26**, the yeast strains SC62-CsSDR-AKR1C4-CYP512W2-r, SC62-CYP512W6-r-CYP512W2-r, SC62-CYP512W6-r, SC62-GlAT-r-CYP512W2-r, SC62-BsAT-r-CsSDR-AKR1C4-CYP512W2-r underwent liter-scale fermentation. Colonies were selected and cultured in 3 mL of SC-His-Leu-Ura-Trp (SC-HLUT) medium until reaching an $OD_{600}$ of 2.5-4, and then inoculated into 50 mL of SC-HLUT. Seed cultures were inoculated into 2 L shake flasks containing 400 mL of YPD24 with 200 mg/L of G418 and 200 mg/L hygromycin, for a total volume of 5 L. After 5 days of fermentation, 4 L of ethyl acetate was added and mixed with a magnetic stirrer for 1 h to extract metabolites. All extraction processes were repeated twice. The organic layer was collected, evaporated, and redissolved in 20 mL methanol for purification.

Purification of the crude extracts was initially carried out on a preparative Agilent 1200 LC system

(Waldbronn, Germany). Solvent A was ultrapure water, and solvent B was methanol. The first round of purification was performed using a Kromasil 100-10-C18 column (20 mm × 250 mm). The samples were eluted from the column with a linear gradient of 80-100% B for 30 min and 100% B for 30 min at a flow rate of 10 mL/min. GA-Mk, GA-T, GA-S, GA-R, **1**, **10**, **12**, **14**, and **26** were purified in a second round using an Elite Hypersil ODS2 column (10 mm × 250 mm) and eluted with a linear gradient of 80-100% B for 60 min and 100% B for 30 min at a flow rate of 4 mL/min. **2**, **6**, **7**, **8**, and **9** were purified in a second round using a Waters XBridge BEH C18 OBD Prep column (10 mm × 150 mm) on a Waters LC Prep 150 System (Milford, USA) and eluted with a linear gradient of 60-100% B for 30 min and 100% B for 30 min at a flow rate of 2 mL/min. The pure target compounds within the elutes were concentrated to dry powder.

**Alkaline hydrolysis of GAs**

GA-R and GA-T were dissolved in 4 mL mixture of 3 M NaOH aqueous solution and methanol at a substrate to NaOH ratio of 1:12. They were stirred continuously for 72 h at room temperature. Subsequently, the pH of the mixture was adjusted to 3 by the slow addition of 1 M HCl and concentrated by evaporation. The resulting solution was diluted to 1 mL with distilled water and then extracted twice using equal volumes of ethyl acetate. The organic phase was collected after centrifugation (12,000 *g*, 10 min), and the oily liquid obtained after evaporation was the alkaline hydrolysis product.

**Yeast transformation and fermentation**

The standard lithium acetate method[76] was employed for yeast transformation. Yeast cells were

cultured at 30 °C with shaking at 220 rpm in liquid culture. Three parallel colonies of each transformant, unless otherwise indicated, were selected and inoculated into 12 mL round-bottom test tubes containing 3 mL of the appropriate medium and incubated for 24 h until reaching an $OD_{600}$ of 2.5-4. They were then inoculated into 12 mL round-bottom test tubes containing 3 mL of YPD24 or 250 mL shake-flasks containing 50 mL of YPD24 with appropriate concentrations of hygromycin and/or G418 at an initial $OD_{600}$ of 0.05.

For galactose induction, yeast cells grown in YPD24 culture were centrifuged (25 °C, 2,000 *g*, 5 min) and washed twice with sterile distilled water. After centrifugation, the cell precipitate was resuspended in 50 mL of YPG24 (containing 10 g/L of yeast extract, 20 g/L of beef peptone, 20 g/L of galactose, and 40 g/L glycerol) medium for further incubation.

After fermentation for 120 h or 168 h, 1 mL of fermentation broth was extracted twice with 800 μL of ethyl acetate. The ethyl acetate layer was collected, evaporated, and redissolved in methanol for HPLC analysis.

**Yeast chromosome integration and fluorescence sorting**

For single integration, H1 and H2 sites were selected due to the high expression level exhibited by neighboring genes [77]. The same gRNA sequences were assembled into pCRCT plasmids [78]. The donor sequence contained a CYP512W2 expression cassette flanked by 500 bp left and right homology arms for CRISPR integration site. Plasmids were transformed into yeast strain SC62. After incubation for 2-3 days, colonies were randomly selected to extract genomic DNA, and

genotypes were confirmed by PCR amplification of the flanked regions of the integration sites and followed by Sanger sequencing.

For multiple integration at the EGFP site, two gRNA targeting 122 bp and 309 bp downstream of EGFP were designed and assembled into pCRCT. The donor sequence contained CYP512W2$^{I108A}$ and RFP expression cassettes flanked by 500 bp left and right homology arms for the CRISPR integration site. One μg of the pCRCT plasmid and an equimolar amount of linearized donor fragment were introduced into strain SC62. Yeast cells were collected by centrifugation (25 °C, 6,000 *g,* 2 min) after transformation, resuspended in 500 μL of YPD medium, and incubated at 30 °C 220 rpm for 3 h. The transformation products were washed twice with sterile distilled water and then plated on SC-HUT solid medium.

All colonies on the transformation plates were collected in 3 mL of sterile water and inoculated at 1% v/v in 12 mL round-bottomed test tubes containing 3 mL of SC-HUT liquid medium and incubated until reaching an $OD_{600}$ of 2.5-4. Cells were collected by centrifugation (6,000 *g*, 2 min), washed with PBS buffer, and diluted to an $OD_{600}$ of 0.5-0.6. Five mL of cell suspension was taken for FACS analysis (FACSAria II, BD Bioscience, New Jersey, USA) to sort target cells. The detection threshold was set for forward scatter (FSC) and side scattering (SSC) to identify yeast cell populations and exclude debris and attachment interference. GFP fluorescence was analyzed on the FITC channel (488 nm laser, 525 nm filter), and RFP fluorescence was analyzed on the PE channel (488 nm laser, 585 nm filter). The 0.02% yeast cells (~8000-10000) with no green fluorescence and the strongest red fluorescence were collected in 3 mL of SC-HUT liquid medium, incubated at 30°C

and 120 rpm for 4 h, and then inoculated at 5% v/v in YPD solid medium for 2-3 days of growth.

Sixty single clones were randomly selected from YPD plates for fermentation in 3 mL of YPD24 medium, and the two transformants with the highest yield of GA-Jb were selected and inoculated into YPD medium containing 1 g/L 5-FOA for subculture to recycle the CRISPR plasmid. After three generations, an appropriate amount of culture was inoculated on a YPD plate for 2-3 days. Then, 10 clones were randomly selected and inoculated on an SC-HUT plate for verification. No yeast colonies grew, indicating the successful recycling of the CRISPR plasmid. Three transformants were selected for shake flask fermentation.

For multiple integration at RFP site, one gRNA targeting 191 bp downstream of RFP was designed and assembled into pCRCT. The donor sequence for producing GA-Ja contained CsSDR, AKR1C4, and EGFP* (synonymous mutated EGFP) expression cassettes flanked by 500 bp left and right homology arms for the CRISPR integration site. The donor sequence for producing TLTOA contained CYP512W6, CsSDR, AKR1C4, and EGFP* (synonymous mutated EGFP) expression cassettes flanked by 500 bp left and right homology arms for the CRISPR integration site. After following the same procedure as that for multiple integration at the EGFP site, colonies on the transformation plates were subjected to FACS screening. The 1% yeast cells (~10000) with strongest green fluorescence and no red fluorescence were collected in 3 mL of SC-HUT liquid medium, incubated at 30°C and 120 rpm for 4 h, and then inoculated at 5% v/v in YPD solid medium for 2-3 days of growth. Thirty single clones were randomly selected from YPD plates for fermentation in 3 mL of YPD24 medium. The transformants with the highest yield of GA-Ja or TLTOA were

selected and inoculated into YPD medium containing 1 g/L 5-FOA for subculture to recycle the CRISPR plasmid.

## Supplemental Notes

### Supplementary Note S1. General descriptions for NMR analysis

The NMR spectra (Supplementary Table S8 and Figs. S2-S7) of **1** revealed a similar structure to that of GA-Jb[74]. However, differences were observed. Specifically, a carbon with a chemical shift ($\delta_C$) 216.73 appeared in the $^{13}$C NMR spectrum (Supplementary Fig. S3), while the signal for H3 was absent in the $^1$H NMR spectrum (Supplementary Fig. S2). This carbon did not exhibit correlated hydrogen signals in the distortions enhancement by polarization transfer (DEPT)-135 (Supplementary Fig. S4) and heteronuclear single quantum coherence (HSQC, Supplementary Fig. S6) spectra. Based on these spectral data, the chemical structure of compound **1** was identified as GA-TR.

The NMR spectra (Supplementary Figs. S12-S18) of **2** were comparable to those of **1**. Notably, the $^{13}$C NMR spectrum displayed C3 at δC 76.78 (Supplementary Fig. S13), and the $^1$H NMR spectrum showed H3 at $\delta_H$ 3.38 (Supplementary Fig. S12). These data indicated that the C3 ketone group of **1** was converted to a hydroxyl group. In comparison with the spectra of GA-Jb, the disappearance of the Nuclear Overhauser Effect Spectroscopy (NOESY) correlation between H3 and H5 suggested that the hydroxyl group at C3 was in the α-configuration. Consequently, the chemical structure of **2** was determined to be 3*α*,15*α*-dihydroxylanosta-7,9(11),24-trien-26-oic acid (GA-Ja) (Supplementary Table S8 and Figs. S12-S18).

For peak **6**, the $^1$H NMR spectrum indicated the presence of seven methyl groups, which is three fewer than those of GA-T. Among these methyl groups, one with $\delta_C$ 12.77 and $\delta_H$ 1.83 (d, $J$ = 1.3

Hz, 3H)) exhibited a heteronuclear multiple bond correlation (HMBC) with the sole carbonyl C26 ($\delta_C$ 171.55) (Supplementary Figs. S20, S21 and S25). These spectral characteristics indicated that all three acetyl groups had been removed compared to GA-T (Supplementary Table S8 and Figs. S20-S25). Thus, the chemical structure of **6** was identified as 3*α*,15*α*,22*β*-trihydroxy-lanosta-7,9(11),24-trien-26-oic acid (TLTOA).

In the case of peak **7**, the $^1H$ NMR spectrum showed eight methyl groups, which is two fewer than those of GA-T. One of the eight methyl groups, with $\delta_C$ 12.78 and $\delta_H$ 1.83 (d, $J$ = 1.4 Hz, 3H)), displayed an HMBC signal with carbonyl C26 ($\delta_C$ 171.59). Additionally, another methyl group with $\delta_C$ 21.15 and $\delta_H$ 2.03 (s, 3H) had an HMBC signal with carbonyl C3 ($\delta_C$ 172.65) (Supplementary Figs. S26, S27 and S31). These results suggested that two acetyl groups at C15 and C22 had been removed compared to GA-T (Supplementary Table S8 and Figs. S26-S31). Hence, the chemical structure of **7** was 3*α*-acetoxy-15*α*,22*β*-dihydroxy-lanosta-7,9(11),24-trien26-oic-acid (GA-T2).

For peak **8**, the $^1H$ NMR spectrum showed seven methyl groups (Supplementary Figs. S32), which is two fewer than those of GA-R. One of the methyl groups, with $\delta_C$ 12.83 and $\delta_H$ 1.83 (s, 3H), exhibited an HMBC signal with the sole carbonyl C26 ($\delta_C$ 171.85) (Supplementary Figs. S32, S33 and S37). These data indicated that two acetyl groups had been removed compared to GA-R (Supplementary Table S8 and Figs. S32-S37). Peak **8** was thus identified as 3*α*,22*β*-dihydroxylanosta-7,9(11),24-trien-26-oic acid (DLTOA).

Regarding peak **9**, the $^1H$ NMR spectrum revealed eight methyl groups (Supplementary Figs. S38),

which is one fewer than those of GA-R. One of the methyl groups, with $\delta_C$ 12.79 and $\delta_H$ 1.83 (d, $J$ = 1.3 Hz, 3H), had an HMBC signal with carbonyl C26 ($\delta_C$ 171.68). Another methyl group, with $\delta_C$ 21.15 and $\delta_H$ 2.03 (s, 3H), had an HMBC signal with carbonyl C3 ($\delta_C$ 172.66) (Supplementary Figs. S38, S39 and S43). These results suggested that the acetyl group at C22 was removed compared to GA-R (Supplementary Table S8 and Figs. S38-S43). Consequently, peak **9** was identified as 3*α*-acetoxy-22*β*-hydroxy-lanosta-7,9(11),24-trien-26-oic acid (3*α*-acetoxy-22β-HLTOA).

The NMR spectra of **10** resembled those of GA-Jb. However, the methine group at C22, with $\delta_C$ 73.4 and $\delta_H$ 3.74 (m, 1H), was similar to that of compound **6**. These spectral data indicated that the chemical structure of compound **10** was the C22*β*-hydroxylated GA-Jb, 3*β*,15*α*,22*β*-trihydroxylanosta-7,9(11),24-trien-26-oic acid (3*β*-TLTOA) (Supplementary Table S8 and Figs. S45-S50).

For compound **12**, the NMR spectra were similar to those of GA-HLDOA. The methine group at C22, with $\delta_C$ 73.48 and $\delta_H$ 3.78 (m, 1H), was similar to that of compound **6**. These data suggested that the chemical structure of compound **12** was the C22*β*-hydroxylated HLDOA, 3*β*,22*β*-dihydroxylanosta-8,24-dien-26-oic acid (DLDOA) (Supplementary Table S8 and Figs. S51-S56).

The NMR spectra of **14** were similar to those of GA-Jb. Differences included C15 at $\delta_C$ 77.37 and $\delta_H$ 5.07 (m, 1H), the presence of a carbonyl C at $\delta_C$ 171.24, and an additional methyl group at $\delta_C$ 21.44 and $\delta_H$ 2.09 (s, 3H). The carbonyl C showed HMBC signals with H15 and the extra methyl group. These results indicated that an acetyl group was introduced at the OH-15 position of GA-Jb.

Therefore, the chemical structure of **14** was GA-TN (Supplementary Table S8 and Figs. S64-S69).

The NMR spectra of **26** were similar to those of GA-Ja. Differences included C3 at $\delta_C$ 79.7 and $\delta_H$ 4.65 (t, $J$ = 2.7 Hz, 1H), the presence of a carbonyl C at $\delta_C$ 172.62, and an additional methyl group at $\delta_C$ 21.16 and δH 2.03 (s, 3H). The carbonyl C exhibited HMBC signals with H3 and the extra methyl group. These data indicated that an acetyl group was introduced at the OH-3 position of GA-Ja (Supplementary Table S8 and Figs. S75-S80). Thus, the chemical structure of **26** was GA-Mf.

**Supplementary Note S2. Chemical structure speculation corresponding to peaks 20, 21, 28-33.**

Peaks **20** and **21** had earlier liquid chromatography (LC) retention times (9.4 min, 9.9 min) compared to GA-P (10.6 min), which contains two acetoxy groups and one hydroxyl group, but had later LC retention times than TLTOA (8.8 min), which contains three hydroxyl groups. These observations suggested that one hydroxyl group in peaks **20** and **21** might have been substituted with an acetoxy group. The mass spectral information of peak **20** shows a primary *m/z* at 469 $[M-HOAc+H]^+$, indicating a preference for the removal of the acetoxy group. In contrast, peak **21**, with a primary *m/z* at 511 $[M-H_2O+H]^+$, appears to retain the acetoxy group. Based on these data, the structures of **20** and **21** may be either 3*α*, 22*β*-dihydroxy-15*α*-acetoxylanosta-7,9(11),24-trien-26-oic acid, or 3*α*,15*α*-dihydroxy-22*β*-acetoxylanosta-7,9(11),24-trien-26-oic acid, with a theoretical M of 528.35.

The mass spectra information of **28** includes fragment ion peaks at 435 $[M-HOAc-H_2O+H]^+$, 453 $[M-HOAc+H]^+$, 495 $[M-H_2O+H]^+$, suggesting the presence of one hydroxyl group and one acetoxy group. Since only one acyltransferase BsAT was expressed in strain SC1-$P_{GAL1}$BsAT-r, the acetoxy group is therefore expected at C3. A total of four TIIGAs may exist in the fermentation extracts of strain SC1-$P_{GAL1}$BsAT-r, among which three known TIIGAs (GA-T-O (R1=*β*-OAc, R2=*α*-OH, R3=H, 18.1 min), GA-Mf (R1=*α*-OAc, R2=*α*-OH, R3=H, 17.0 min), and 3*α*-acetoxy-22*β*-HLTOA (R1=*α*-OAc, R2=H, R3=*β*-OH, 17.5 min)) exhibited different LC retention times with **28** (12.6 min). After excluding these TIIGAs, we speculated that peak **28** might represent 3*β*-acetoxy-22*β*-hydroxylanosta-7, 9(11), 24-trien-26-oic acid (R1=*β*-OAc, R2=H, R3=*β*-OH), with a theoretical M of 512.35.

The mass spectra information of **29** includes fragment ion peaks at at 451 [M-HOAc-$H_2O$+H]$^+$, 469 [M-HOAc+H]$^+$, and 511 [M-$H_2O$+H]$^+$, suggesting the presence of one acetoxy group and two hydroxyl groups. Given its almost identical *m/z* value to that of **20**, but an earlier retention time on HPLC (5.9 min V.S. 9.4 min), we hypothesize that peak **29** is 3*β*,22*β*-dihydroxy-15*α*-acetoxylanosta-7, 9(11), 24-trien-26-oic acid (R1=*β*-OH, R2=*α*-OAc, R3=*β*-OH), or 3*β*,15*α*-dihydroxy-22*β*-acetoxylanosta-7, 9(11), 24-trien-26-oic acid (R1=*β*-OH, R2=*α*-OH, R3=*β*-OAc) with a theoretical M of 528.35.

The mass spectra information of **30** includes fragment ion peaks at 433 [M-2HOAc-$H_2O$+H]$^+$, 451 [M-2HOAc+H]$^+$, 493 [M-HOAc-$H_2O$+H]$^+$, 511 [M-HOAc+H]$^+$, and 553 [M-$H_2O$+H]$^+$, suggesting the presence of one hydroxyl group and two acetoxy groups. With an almost identical *m/z* value to that of GA-P and GA-Mk, and neither a C3 oxidase nor a reductase was introduced in strains SC27-$P_{ADH2}$BsAT-$P_{GAL1}$GlAT-r and SC27-$P_{ADH2}$GlAT-$P_{GAL1}$BsAT-r, peak **30** might be correspond to 3*β*-hydroxy-15*α*,22*β*-diacetoxylanosta-7,9(11),24-trien-26-oic acid (R1=*β*-OH, R2=*α*-OAc, R3=*β*-OAc), or GA-M1 (R1=*β*-OAc, R2=*α*-OH, R3=*β*-OAc), with a theoretical M of 570.36.

The mass spectra information of **31** includes fragment ion peaks at 435 [M-HOAc-$H_2O$+H]$^+$, 453 [M-HOAc+H]$^+$, 495 [M-$H_2O$+H]$^+$, suggesting the presence of one hydroxyl group and one acetoxy group. A total of eight TIIGAs may exist in the fermentation extracts of strains SC27-$P_{ADH2}$BsAT-$P_{GAL1}$GlAT-r and SC27-$P_{ADH2}$GlAT-$P_{GAL1}$BsAT-r, among which six known TIIGAs exhibited later LC retention times than that of **31** (12.4 min). These six TIIGAs are GA-TN (R1=*β*-OH, R2=*β*-OAc,

R3=H, 12.9 min), GA-X (R1=$\alpha$-OH, R2=$\beta$-OAc, R3=H, 15.2 min), GA-T-O (R1=$\beta$-OAc, R2=$\alpha$-OH, R3=H, 18.1 min), GA-Mf (R1=$\alpha$-OAc, R2=$\alpha$-OH, R3=H, 17.0 min), GA-S (R1=$\alpha$-OH, R2=H, R3=$\beta$-OAc, 16.1 min), 3$\alpha$-acetoxy-22$\beta$-HLTOA (R1=$\alpha$-OAc, R2=H, R3=$\beta$-OH, 17.5 min), and 3$\beta$-acetoxy-22$\beta$-hydroxylanosta-7, 9(11), 24-trien-26-oic acid (R1=$\beta$-OAc, R2=H, R3=$\beta$-OH,12.6 min), respectively. After excluding these TIIGAs, we speculate that peak **31** might represent 3$\beta$-hydroxy-22$\beta$-acetoxylanosta-7, 9(11), 24-trien-26-oic acid (R1=$\beta$-OH, R2=H, R3=$\beta$-OAc), with a theoretical M of 512.35.

The mass spectra information of **32** shows fragment ion peaks at 433 $[M\text{-}3HOAc+H]^+$, 493 $[M\text{-}2HOAc+H]^+$, and 553 $[M\text{-}HOAc+H]^+$, suggesting the presence of three acetoxy groups. With an almost identical *m/z* to that of GA-T but a different retention time on HPLC (16.1 min v.s. 14.8 min), we speculate that peak 32 is 3$\beta$,15$\alpha$,22$\beta$-triacetoxylanosta-7,9(11),24-trien-26-oic acid (R1=$\beta$-OAc, R2=$\alpha$-OAc, R3=$\beta$-OAc), with a theoretical M of 612.37.

For peak **33**, the mass spectra information reveals fragment ion peaks at 431 $[M\text{-}2HOAc\text{-}H_2O+H]^+$, 449 $[M\text{-}2HOAc+H]^+$, and 509 $[M\text{-}HOAc+H]^+$. Considering that fragment ion peaks of 433 $[M\text{-}2HOAc\text{-}H_2O+H]^+$, 451 $[M\text{-}2HOAc+H]^+$ and 511 $[M\text{-}HOAc+H]^+$ are detected for all GAs with one hydroxyl group and two acetoxy groups on the backbone (GA-P, GA-T1, and GA-Mk), the reduced 2 *m/z* values detected in **33** implied that a hydroxyl group in the aforementioned GAs may have been dehydrogenated to a keto group. Since a C3 oxidase was introduced in strains SC27-$P_{ADH2}$BsAT-CYP512W6-$P_{GAL1}$GlAT-r-CsSDR-AKR1C4-r and SC27-$P_{ADH2}$GlAT-CYP512W6-$P_{GAL1}$BsAT-r-CsSDR-AKR1C4-r, we speculate that the keto group is at C3. Therefore, peak **33** is

likely to be 3-oxo-15$\alpha$,22$\beta$-diacetoxylanosta-7,9(11),24-trien-26-oic acid (R1=O, R2=$\alpha$-OAc, R3=$\beta$-OAc), with a theoretical M of 568.34.

## Reference


1. Morigiwa, A., Kitabatake, K., Fujimoto, Y. & Ikekawa, N. Angiotensin converting enzyme-inhibitory triterpenes from *Ganoderma lucidim*. *Chem. Pharm. Bull.* **34**, 3025–3028 (1986).
2. Lee, I. et al. Selective cholinesterase inhibition by lanostane triterpenes from fruiting bodies of *Ganoderma lucidum*. *Bioorg. Med. Chem. Lett.* **21**, 6603–6607 (2011).
3. Hajjaj, H., Macé, C., Roberts, M., Niederberger, P. & Fay Laurent, B. Effect of 26-oxygenosterols from *Ganoderma lucidum* and their activity as cholesterol synthesis inhibitors. *Appl. Environ. Microbiol.* **71**, 3653–3658 (2005).
4. Wang, K. et al. Lanostane triterpenes from the Tibetan medicinal mushroom *Ganoderma leucocontextum* and their inhibitory effects on HMG-CoA reductase and α-glucosidase. *J. Nat. Prod.* **78**, 1977–1989 (2015).
5. Dai, Y.C. et al. Anti-inflammatory application of triterpenoid compound in *Ganoderma lucidum* mycelium. *CN 117815246 A* (2024).
6. Cheng, C.R. et al. Cytotoxic triterpenoids from *Ganoderma lucidum*. *Phytochemistry* **71**, 1579–1585 (2010).
7. Kinge, T.R. & Mih, A.M. Secondary metabolites of oil palm isolates of *Ganoderma zonatum* Murill. from Cameroon and their cytotoxicity against five human tumour cell lines. *Afr. J. Biotechnol.* **10**, (2011).
8. Du, G.H., Wang, H.X., Yan, Z., Liu, L.Y. & Chen, R.Y. Anti-tumor target prediction and activity verification of *Ganoderma lucidum* triterpenoids. *China J. Chin. Mater. Med.* **42**, 517–522 (2017).
9. Zhang, W.J. et al. Antiviral effects of two *Ganoderma lucidum* triterpenoids against enterovirus 71 infection. *Biochem. Biophys. Res. Commun.* **449**, 307–312 (2014).
10. Zhang, X.Q. et al. Triterpenoids with neurotrophic activity from *Ganoderma lucidum*. *Nat. Prod. Res.* **25**, 1607–1613 (2011).
11. Amen, Y. et al. Partial contribution of Rho-kinase inhibition to the bioactivity of *Ganoderma lingzhi* and its isolated compounds: insights on discovery of natural Rho-kinase inhibitors. *J. Nat. Med.* **71**, 380–388 (2017).
12. Wang, C.N., Chen, J.S., Shiao, M.S. & Wang, C.T. Activation of human platelet phospholipases C and A2 by various oxygenated triterpenes. *Eur. J. Pharmacol., Mol. Pharmacol.* **267**, 33-42 (1994).
13. Li, C.J., Li, Y.M. & Sun, H.H. New ganoderic acids, bioactive triterpenoid metabolites from the mushroom *Ganoderma lucidum*. *Nat. Prod. Res.* **20**, 985–991 (2006).
14. Liu, J., Shimizu, K. & Kondo, R. Ganoderic acid TR, a new lanostanoid with 5α-reductase inhibitory activity from the fruiting body of *Ganoderma lucidum*. *Nat. Prod. Commun.* **1**, 1934578X0600100501 (2006).
15. Hai Bang, T. & Shimizu, K. Structure–activity relationship and inhibition pattern of reishi-

derived (*Ganoderma lingzhi*) triterpenoids against angiotensin-converting enzyme. *Phytochem. Lett.* **12**, 243–247 (2015).
16. Grienke, U. et al. Pharmacophore-based discovery of FXR-agonists. Part II: Identification of bioactive triterpenes from *Ganoderma lucidum*. *Bioorg. Med. Chem.* **19**, 6779–6791 (2011).
17. Lin, Y.X. et al. Triterpenoids from the fruiting bodies of *Ganoderma lucidum* and their inhibitory activity against FAAH. *Fitoterapia* **158**, 105161 (2022).
18. Zhang, X.Q. et al. Lanostane-type triterpenoids from the mycelial mat of *Ganoderma lucidum* and their hepatoprotective activities. *Phytochemistry* **198**, 113131 (2022).
19. Isaka, M., Chinthanom, P., Kongthong, S., Srichomthong, K. & Choeyklin, R. Lanostane triterpenes from cultures of the Basidiomycete *Ganoderma orbiforme* BCC 22324. *Phytochemistry* **87**, 133–139 (2013).
20. Isaka, M. et al. Antitubercular activity of mycelium-associated *Ganoderma* lanostanoids. *J. Nat. Prod.* **80**, 1361–1369 (2017).
21. Chen, B.S. et al. Triterpenes and meroterpenes from *Ganoderma lucidum* with inhibitory activity against HMGs reductase, aldose reductase and α-glucosidase. *Fitoterapia* **120**, 6–16 (2017).
22. Akihisa, T. et al. Anti-inflammatory and anti-tumor-promoting effects of triterpene acids and sterols from the fungus *Ganoderma lucidum*. *Chem. Biodiversity* **4**, 224–231 (2007).
23. Dai, Y.C. et al. Triterpenoids extracted from *Ganoderma sinense* fermentation mycelia and their antitumor application. *CN 115746076 A* (2023).
24. Wang, C.N., Chen, J.C., Shiao, M.S. & Wang, C.T. The inhibition of human platelet function by ganodermic acids. *Biochem. J.* **277**, 189-197 (1991).
25. Isaka, M. et al. Antitubercular lanostane triterpenes from cultures of the basidiomycete *Ganoderma* sp. BCC 16642. *J. Nat. Prod.* **79**, 161–169 (2016).
26. Teng, L.M. et al. Lanostane triterpenoids from mycelia-associated *Ganoderma sinense* and their anti-inflammatory activity. *Phytochemistry* **215**, 113870 (2023).
27. Li, C.H. et al. Ganoderic acid X, a lanostanoid triterpene, inhibits topoisomerases and induces apoptosis of cancer cells. *Life Sci.* **77**, 252–265 (2005).
28. Li, Y.B., Liu, R.M. & Zhong, J.J. A new ganoderic acid from *Ganoderma lucidum* mycelia and its stability. *Fitoterapia* **84**, 115–122 (2013).
29. Liu, R.M. & Zhong, J.J. Ganoderic acid Mf and S induce mitochondria mediated apoptosis in human cervical carcinoma HeLa cells. *Phytomedicine* **18**, 349–355 (2011).
30. Chen, N.H. & Zhong, J.J. Ganoderic acid Me induces G1 arrest in wild-type p53 human tumor cells while G1/S transition arrest in p53-null cells. *Process Biochem.* **44**, 928–933 (2009).
31. Feng, N. et al. Triterpene compound extracted from *Ganoderma lucidum* mycelia and application thereof in preparation of anti-inflammatory drug. *CN 115894591 A* (2023).
32. Wang, G. et al. Enhancement of IL-2 and IFN-γ expression and NK cells activity involved in the anti-tumor effect of ganoderic acid Me *in vivo*. *Int. Immunopharmacol.* **7**, 864–870 (2007).
33. Que, Z.J. et al. Ganoderic acid Me induces the apoptosis of competent T cells and increases the proportion of Treg cells through enhancing the expression and activation of indoleamine 2,3-dioxygenase in mouse lewis lung cancer cells. *Int. Immunopharmacol.*

**23**, 192–204 (2014).
34. Chen, N.H., Liu, J.W. & Zhong, J.J. Ganoderic acid Me inhibits tumor invasion through down-regulating matrix metalloproteinases 2/9 gene expression. *J. Pharmacol. Sci.* **108**, 212–216 (2008).
35. Zhu, X.L. et al. NMR attribution and bioactivity evaluation of a triterpene in mycelia of *Ganoderma lingzhi*. *Mycosystema* **39**, 1551–1558 (2020).
36. Ouyang, J.J., Wang, Y.Q. & Tang, W. Ganoderic acid restores the sensitivity of multidrug resistance cancer cells to doxorubicin. *Adv. Mater. Res.* **834-836**, 573–576 (2014).
37. Liu, R.M. et al. Structurally related ganoderic acids induce apoptosis in human cervical cancer HeLa cells: Involvement of oxidative stress and antioxidant protective system. *Chem.-Biol. Interact.* **240**, 134–144 (2015).
38. Yue, Y.W. et al. Active components in mycelia of sporeless *Ganoderma lingzhi*. *Mycosystema* **39**, 128–136 (2020).
39. Liu, R.M., Li, Y.B. & Zhong, J.J. Anti-proliferation and induced mitochondria-mediated apoptosis of ganoderic acid Mk from *Ganoderma lucidum* mycelia in cervical cancer HeLa cells. *Lat. Am. J. Pharm.* **31**, 43–50 (2012).
40. Xu, K., Liang, X., Gao, F., Zhong, J.J. & Liu, J.W. Antimetastatic effect of ganoderic acid T *in vitro* through inhibition of cancer cell invasion. *Process Biochem.* **45**, 1261–1267 (2010).
41. Liu, R.m., Li, Y.b. & Zhong, J.j. Cytotoxic and pro-apoptotic effects of novel ganoderic acid derivatives on human cervical cancer cells *in vitro*. *Eur. J. Pharmacol.* **681**, 23–33 (2012).
42. Tang, W., Liu, J.W., Zhao, W.M., Wei, D.Z. & Zhong, J.J. Ganoderic acid T from *Ganoderma lucidum* mycelia induces mitochondria mediated apoptosis in lung cancer cells. *Life Sci.* **80**, 205–211 (2006).
43. Yang, S.X. et al. Toxic lanostane triterpenes from the basidiomycete *Ganoderma amboinense*. *Phytochem. Lett.* **5**, 576–580 (2012).
44. Yangchum, A. et al. Lanostane triterpenoids from cultivated fruiting bodies of basidiomycete *Ganoderma mbrekobenum*. *Phytochemistry* **196**, 113075 (2022).
45. Huang, S.Z. et al. Lanostane-type triterpenoids from the fruiting body of *Ganoderma calidophilum*. *Phytochemistry* **143**, 104–110 (2017).
46. Adams, M. et al. Antiplasmodial lanostanes from the *Ganoderma lucidum* mushroom. *J. Nat. Prod.* **73**, 897–900 (2010).
47. Shiao, M.S., Lin, L.J. & Yeh, S.F. Triterpenes in *Ganoderma lucidum*. *Phytochemistry* **27**, 873-875 (1988).
48. Isaka, M. et al. Lanostane triterpenoids from cultivated fruiting bodies of the basidiomycete *Ganoderma orbiforme*. *Phytochem. Lett.* **21**, 251–255 (2017).
49. Zhang, H.J. et al. Natural anti-HIV agents. Part IV. Anti-HIV constituents from *Vatica cinerea*. *J. Nat. Prod.* **66**, 263–268 (2003).
50. Li, C.J., Yin, J.H., Guo, F.J., Zhang, D.C. & Sun, H.H. Ganoderic acid Sz, a new lanostanoid from the mushroom *Ganoderma lucidum*. *Nat. Prod. Res.* **19**, 461–465 (2005).
51. Shiao, M.S., Lin, L.J. & Yeh, S.F. Triterpenes from *Ganoderma lucidum*. *Phytochemistry* **27**, 2911-2914 (1988).
52. Lin, L.J., Shiao, M.S. & Yeh, S.F. Seven new triterpenes from *Ganoderma lucidum*. *J. Nat. Prod.* **51**, 918-924 (1988).
53. Peng, X.R. et al. Ganocochlearic acid A, a rearranged hexanorlanostane triterpenoid, and

cytotoxic triterpenoids from the fruiting bodies of *Ganoderma cochlear*. *RSC Adv.* **5**, 95212–95222 (2015).
54. Iwatsuki, K. et al. Lucidenic acids P and Q, methyl lucidenate P, and other triterpenoids from the fungus *Ganoderma lucidum* and their inhibitory effects on Epstein-Barr virus activation. *J. Nat. Prod.* **66**, 1582–1585 (2003).
55. Ma, Q.Y. et al. Lanostane triterpenoids with cytotoxic activities from the fruiting bodies of *Ganoderma hainanense*. *J. Asian Nat. Prod. Res.* **15**, 1214–1219 (2013).
56. Hu, G.S. et al. Optimization of culture condition for ganoderic acid production in *Ganoderma lucidum* liquid static culture and design of a suitable bioreactor. *Molecules* **23**, 2563 (2018).
57. Wang, J.L., Li, Y.B., Liu, R.M. & Zhong, J.J. A new ganoderic acid from *Ganoderma lucidum* mycelia. *J. Asian Nat. Prod. Res.* **12**, 727–730 (2010).
58. Xu, J.W., Xu, Y.N. & Zhong, J.J. Production of individual ganoderic acids and expression of biosynthetic genes in liquid static and shaking cultures of *Ganoderma lucidum*. *Appl. Microbiol. Biotechnol.* **85**, 941–948 (2010).
59. Zhao, W., Xu, J.W. & Zhong, J.J. Enhanced production of ganoderic acids in static liquid culture of *Ganoderma lucidum* under nitrogen-limiting conditions. *Bioresour. Technol.* **102**, 8185–8190 (2011).
60. Xu, Y.N. & Zhong, J.J. Impacts of calcium signal transduction on the fermentation production of antitumor ganoderic acids by medicinal mushroom *Ganoderma lucidum*. *Biotechnol. Adv.* **30**, 1301–1308 (2012).
61. Xu, Y.N., Xia, X.X. & Zhong, J.J. Induction of ganoderic acid biosynthesis by $Mn^{2+}$ in static liquid cultivation of *Ganoderma lucidum*. *Biotechnol. Bioeng.* **111**, 2358–2365 (2014).
62. Xu, Y.N., Xia, X.X. & Zhong, J.J. Induced effect of $Na^{+}$ on ganoderic acid biosynthesis in static liquid culture of *Ganoderma lucidum* via calcineurin signal transduction. *Biotechnol. Bioeng.* **110**, 1913–1923 (2013).
63. Zhang, D.H. et al. Overexpression of the homologous lanosterol synthase gene in ganoderic acid biosynthesis in *Ganoderma lingzhi*. *Phytochemistry* **134**, 46–53 (2017).
64. Fei, Y., Li, N., Zhang, D.H. & Xu, J.W. Increased production of ganoderic acids by overexpression of homologous farnesyl diphosphate synthase and kinetic modeling of ganoderic acid production in *Ganoderma lucidum*. *Microb. Cell Fact.* **18**, 115 (2019).
65. Li, H.J. et al. Enhancement of ganoderic acid production by constitutively expressing *Vitreoscilla* hemoglobin gene in *Ganoderma lucidum*. *J. Biotechnol.* **227**, 35–40 (2016).
66. Zhou, J.S. et al. Enhanced accumulation of individual ganoderic acids in a submerged culture of *Ganoderma lucidum* by the overexpression of squalene synthase gene. *Biochem. Eng. J.* **90**, 178–183 (2014).
67. Zhang, D.H. et al. Overexpression of the squalene epoxidase gene alone and in combination with the 3-hydroxy-3-methylglutaryl coenzyme a gene increases ganoderic acid production in *Ganoderma lingzhi*. *J. Agric. Food Chem.* **65**, 4683–4690 (2017).
68. Isaka, M. et al. Lanostane triterpenoids from cultivated fruiting bodies of the wood-rot basidiomycete *Ganoderma casuarinicola*. *Phytochemistry* **170**, 112225 (2020).
69. Chinthanom, P. et al. Chemical analysis and antitubercular activity evaluation of the dried mycelial powders of the basidiomycete *Ganoderma australe* TBRC-BCC 22314. *Fitoterapia* **169**, 105597 (2023).

70. Chinthanom, P. et al. Semisynthetic modifications of antitubercular lanostane triterpenoids from *Ganoderma*. *J. Antibiot.* **74**, 435–442 (2021).
71. Shiao, M.S., Lin, L.J., Yeh, S.F. & Chou, C.S. Two new triterpenes of the fungus *Ganoderma lucidum*. *J. Nat. Prod.* **50**, 886-890 (1987).
72. Yue, Y.W. et al. Determination and chemotaxonomic analysis of lanostane triterpenoids in the mycelia of *Ganoderma* spp. using ultra-performance liquid chromatography-tandem mass spectrometry (I). *Planta Med.* **89**, 1505–1514 (2023).
73. Katahira, S., Mizuike, A., Fukuda, H. & Kondo, A. Ethanol fermentation from lignocellulosic hydrolysate by a recombinant xylose-and cellooligosaccharide-assimilating yeast strain. *Appl. Microbiol. Biotechnol.* **72**, 1136–1143 (2006).
74. Yuan, W. et al. Biosynthesis of mushroom-derived type II ganoderic acids by engineered yeast. *Nat. Commun.* **13**, 7740 (2022).
75. Lan, X., Yuan, W., Wang, M. & Xiao, H. Efficient biosynthesis of antitumor ganoderic acid HLDOA using a dual tunable system for optimizing the expression of CYP5150L8 and a *Ganoderma* P450 reductase. *Biotechnol. Bioeng.* **116**, 3301–3311 (2019).
76. Gietz, R.D. & Schiestl, R.H. High-efficiency yeast transformation using the LiAc/SS carrier DNA/PEG method. *Nat. Protoc.* **2**, 31–34 (2007).
77. Baek, S. et al. The yeast platform engineered for synthetic gRNA-landing pads enables multiple gene integrations by a single gRNA/Cas9 system. *Metab. Eng.* **64**, 111–121 (2021).
78. Bao, Z. et al. Homology-integrated CRISPR–Cas (HI-CRISPR) system for one-step multigene disruption in *Saccharomyces cerevisiae*. *ACS Synth. Biol.* **4**, 585–594 (2015).
